# Immunomodulation by bepirovirsen may induce killing of infected hepatocytes (B-Together study)

**DOI:** 10.1007/s12072-025-10917-0

**Published:** 2025-10-03

**Authors:** Shilpy Joshi, Johannes M. Freudenberg, Jennifer M. Singh, William T. Jordan, Leigh Felton, Susan Dixon, Melanie Paff, Dickens Theodore, Jill Walker

**Affiliations:** 1 Clinical Biomarkers and Correlative Sciences, Precision Medicine, GSK, Collegeville, PA USA; 2 Translational Omics, GSK, Collegeville, PA USA; 3 Clinical Development, GSK, London, UK; 4 Medical Development, GSK, Collegeville, PA USA; 5 Clinical Development, GSK, Durham, NC USA; 6 Clinical Biomarkers, GSK, San Francisco, CA USA

**Keywords:** Phase 2b study, Peripheral longitudinal biomarker analysis, Mechanism of action, Immune system, Unconjugated antisense oligonucleotide, Alanine aminotransferase (ALT), Hepatitis B surface antigen (HBsAg), Virological response, Proteomics, Whole blood transcriptomics, Peripheral blood mononuclear cell (PBMC) immunophenotyping

## Abstract

**Background:**

Bepirovirsen is an investigational drug; its multimodal mechanism of action (MoA) is under evaluation. Observations in treated participants show transient alanine aminotransferase (ALT) increases, alongside hepatitis B surface antigen (HBsAg) declines. We investigated bepirovirsen’s MoA in relation to virological response, hepatocyte death, and ALT increases.

**Methods:**

In B-Together (NCT04676724), 108 participants on stable nucleos(t)ide analogs received bepirovirsen for 24 (Arm 1) or 12 (Arm 2) Weeks, then up to 24 Weeks of pegylated interferon-α-2a. This post hoc peripheral longitudinal biomarker exploratory analysis examined serum proteomics and whole blood transcriptomics from peripheral blood mononuclear cell samples from 82 participants. Relative expressions of immune- and disease-related biomarkers were measured, and differential expression determined across arms and response subgroups.

**Results:**

Increases from baseline in mean expression of serum proteins with immune effector and apoptotic functions (Week 3) and transcripts associated with immune cell proliferation and activation (Week 5) were observed regardless of arm or response subgroup. By Week 8, serum liver and apoptosis-specific proteins were increased; this was more pronounced in responders than non-responders, with the difference more marked in Arm 1 versus Arm 2. Increased abundance of these proteins was highly correlated with ALT levels, which were often associated with transient hepatitis B virus (HBV) DNA elevations and HBsAg decreases.

**Conclusions:**

These findings provide evidence that bepirovirsen may modulate the immune system to facilitate infected hepatocyte killing in chronic HBV infection in addition to its direct antiviral effects; therefore, ALT increases could reflect a therapeutic response to bepirovirsen.

**Clinical trial number:**

NCT04676724 and NCT04449029.

**Graphical abstract:**

B-Together peripheral longitudinal biomarker analysis provides insights into bepirovirsen’s mechanism of action

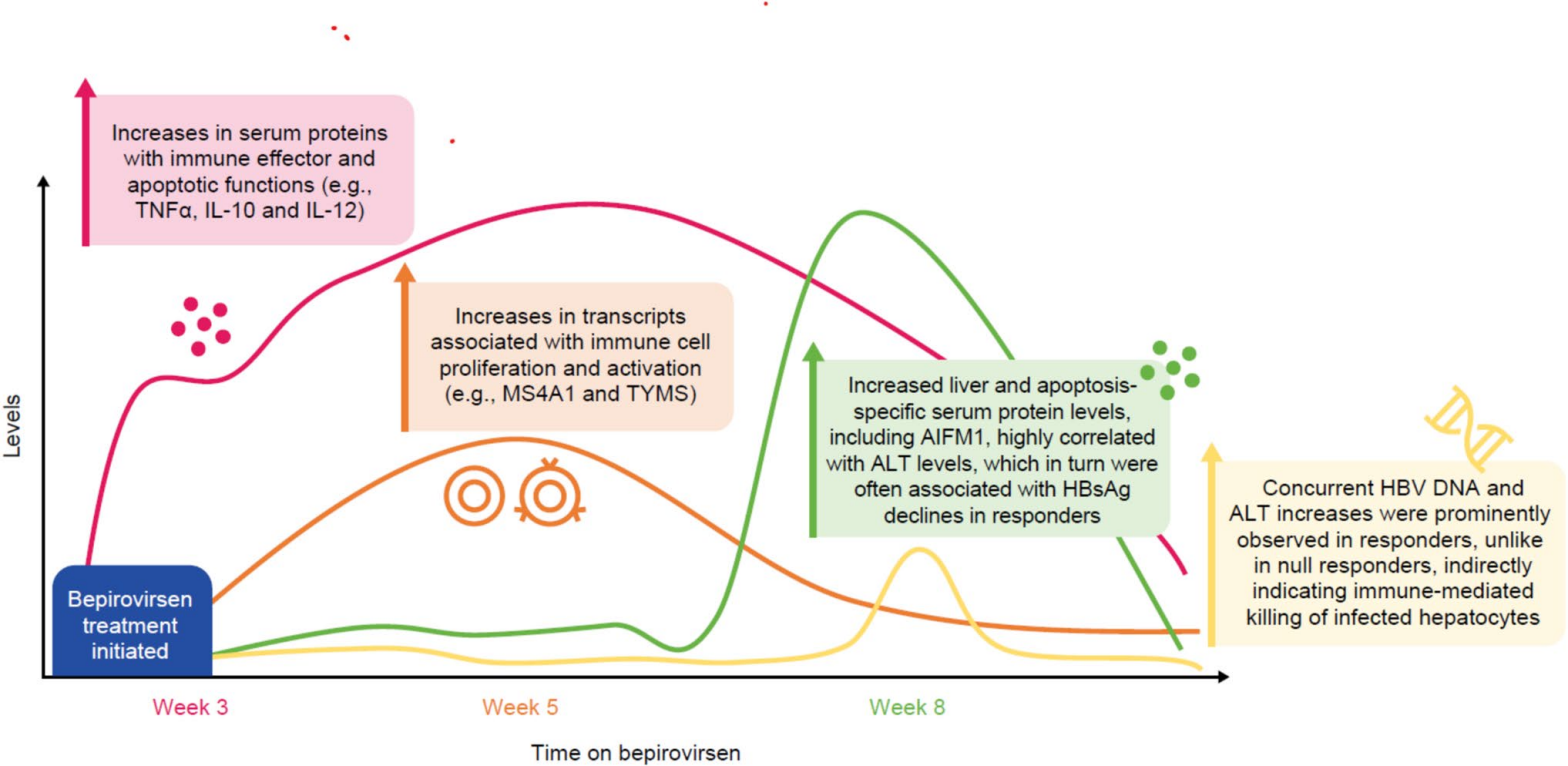

**Supplementary Information:**

The online version contains supplementary material available at 10.1007/s12072-025-10917-0.

## Introduction

Chronic hepatitis B virus (HBV) infection has a high clinical burden [1]. Bepirovirsen is a novel unconjugated antisense oligonucleotide (ASO) being evaluated in the Phase 3 B-Well studies (NCT05630820 and NCT05630807) in chronic HBV infection as a finite therapy to help achieve functional cure, defined as loss of hepatitis B surface antigen (HBsAg) and HBV DNA below the lower limit of quantification (LLOQ) at 24 Weeks off all therapy [2]. It is a single-stranded nucleic acid that binds and targets all HBV RNAs, thus reducing HBV DNA levels and viral proteins, including HBsAg [3]. Unlike investigational short interfering RNAs [4], bepirovirsen is not conjugated to a N-acetylgalactosamine for targeted hepatocyte delivery [5, 6]. This unconjugation may facilitate uptake to non-parenchymal liver cells, as shown in mice [6], where bepirovirsen is hypothesized to stimulate the immune system [5, 7]. In the Phase 2b B-Together study, treatment with bepirovirsen followed by pegylated interferon-α-2a (Peg-IFN), along with background nucleos(t)ide analog (NA) therapy, achieved HBV DNA and HBsAg loss for 24 Weeks after end of Peg-IFN treatment in 9% (24-Week bepirovirsen arm) and 15% (12-Week bepirovirsen arm) of participants [8]. Participants with lower baseline HBsAg levels were more likely to respond to bepirovirsen treatment, as previously observed in the Phase 2b B-Clear study [8, 9].

Transient alanine aminotransferase (ALT) increases ≥ 3 times the upper limit of normal (ULN) were observed in some B-Together participants during the bepirovirsen treatment period, as also previously observed in B-Clear [8, 9]. Most ALT increases were associated with concurrent HBsAg decline [8, 9], leading to the hypothesis that these increases may be therapeutic and linked with killing of infected hepatocytes.

To probe this hypothesis, this B-Together post hoc analysis investigated bepirovirsen’s immune mechanism of action and its role with respect to virological response, surrogate markers associated with hepatocyte death, and ALT increases. A peripheral longitudinal exploratory analysis of biomarker data assessed bepirovirsen’s effect on soluble biomarkers (including circulating cytokines and chemokines), markers of immune cell function, and the relationship between these biomarkers and virological biomarkers, including HBsAg.

## Materials and methods

While biomarker evaluation was included as an exploratory endpoint in B-Together (NCT04676724), analyses detailed here were performed post hoc. A set of B-Clear (NCT04449029) data was analyzed post hoc to support findings (Supplementary Methods).

### Study design

**Fig. 1:**
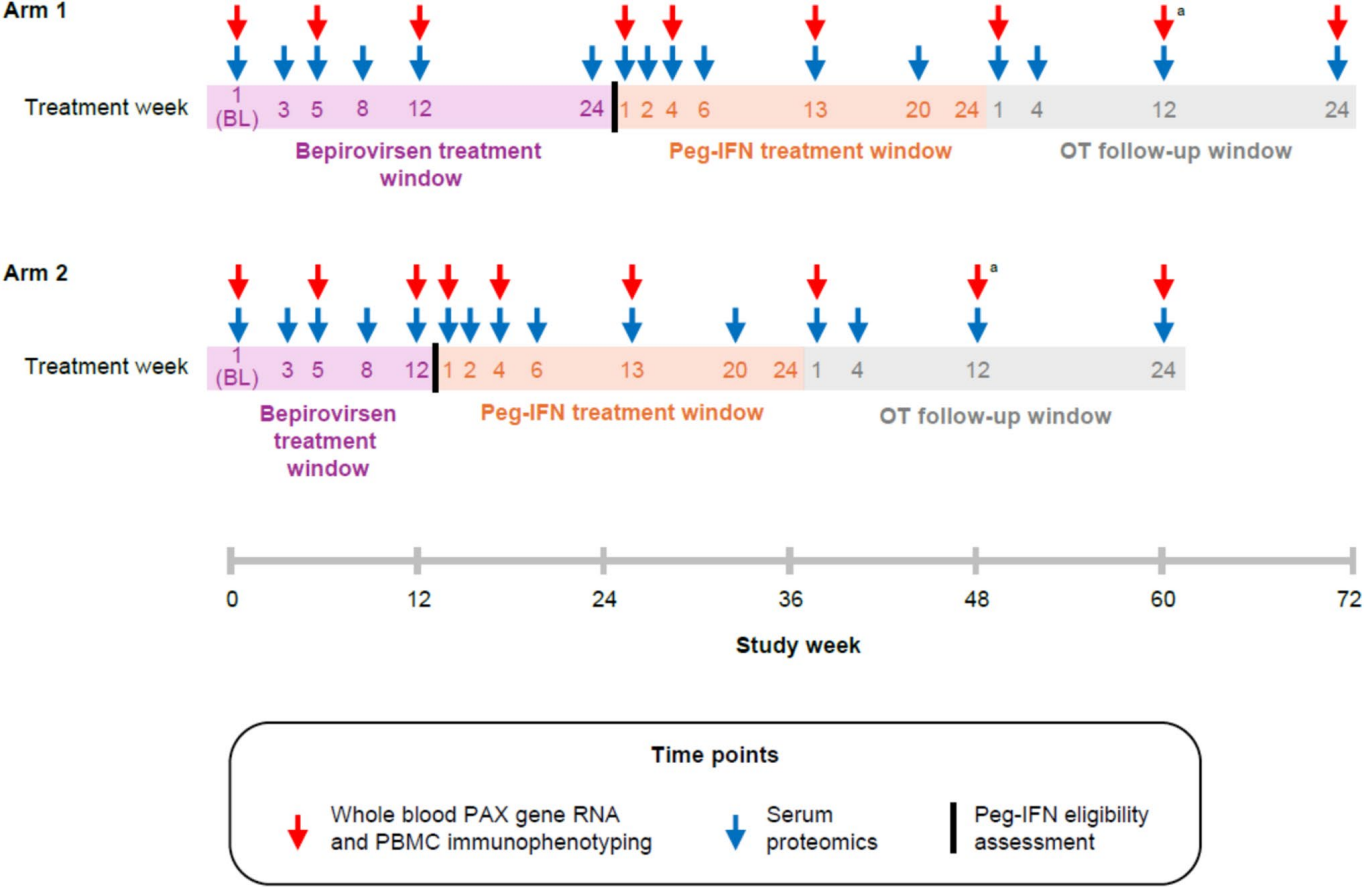
B-Together study design and sample collection logistics. All biomarker samples were collected pre-dose. ^a^Only whole blood samples, and not PBMCs, were collected at Week 12 off-treatment for both arms. *BL* baseline, *OT* off-treatment, *PAX* paired-box, *PBMC* peripheral blood mononuclear cell, *Peg-IFN* pegylated interferon-α-2a

B-Together was a Phase 2b, multicenter, randomized, open-label study in participants with chronic HBV infection, assessing efficacy and safety of sequential treatment with bepirovirsen followed by Peg-IFN [8]. Eligibility criteria (Supplementary Methods) have been described previously [8, 9]. Participants were randomized 1:1 to bepirovirsen 300 mg Weekly (plus loading dose on Days 4 and 11) for 24 (Arm 1) or 12 (Arm 2) Weeks followed by up to 24 Weeks of Peg-IFN 180 μg weekly (Fig. 1). Participants were monitored for up to 72 Weeks [8].

### Virological response subgroups

Primary outcome responders (referred to as responders throughout) were participants who achieved HBsAg and HBV DNA < LLOQ (HBsAg, < 0.05 IU/mL; HBV DNA, < 20 IU/mL) at each analysis window in the 24 Weeks after planned end of sequential treatment, without initiating new treatment to suppress viral replication. Non-responders were participants who did not meet the primary outcome or had missing data in the off-treatment period. Null responders were non-responders who did not have two or more consecutive visits with a > 0.4 log_10_ HBsAg reduction while on treatment.

### Biomarker sample collection

All B-Together randomized participants from the rest of the world population (excluding China), who had been assessed for the primary outcome and who received at least one dose of bepirovirsen treatment and ≥ 12 doses of Peg-IFN were included in biomarker analyses [8]. Proteomic analysis of longitudinal serum samples evaluated expression of soluble biomarkers, such as circulating cytokines. Whole blood transcriptomic analysis investigated messenger RNA (mRNA) expression and cell activation status. Peripheral blood mononuclear cell (PBMC) immunophenotyping assessed frequencies and activation status of immune cell subsets.

Serum samples for proteomic analysis, whole blood samples for transcriptomic analysis, and PBMCs for immunophenotyping were collected pre-dose at timepoints specified in Fig. 1. Therefore, Week 1 constitutes baseline collection, Peg-IFN Week 1 is considered bepirovirsen end of treatment, off-treatment Week 1 is considered Peg-IFN end of treatment, and off-treatment Week 24 is end of study. Due to scarcity of PBMC samples collected in Peg-IFN and off-treatment periods, only results from the bepirovirsen period were investigated. Although proteomic and whole blood transcriptomic results are displayed across all collection timepoints for completeness, this analysis focused on the bepirovirsen treatment phase. Observations during the Peg-IFN treatment phase remain out of scope for this manuscript.

Further details of biomarker analyses and methods for proteomics, whole blood transcriptomics and flow cytometry analyses, as well as assay details for HBsAg and HBV DNA quantification are described in Supplementary Methods.

### Differential expression and pathway analyses

Relative expressions of multi-omic biomarkers were measured at baseline (Week 1) and post-baseline at multiple timepoints during bepirovirsen, Peg-IFN, and off-treatment phases and included in a differential expression analysis comparing serum proteomic expression and whole blood transcriptomic profiles, respectively. To determine differential expression of proteins and transcripts, multivariate models were fit that included treatment arms and virological response subgroups. Individual proteins/transcripts had to meet the significance threshold for differential expression at Week 3/5/8 (1.5-fold absolute change; false discovery rate [FDR] of 10%) in at least one subgroup comparison for inclusion in the corresponding heatmap. Over-representation pathway analysis is further described in the Supplementary Methods. R code is provided in the Supplementary Materials.

Formal hypothesis testing was not performed on PBMC data owing to high rate of missing/failed samples.

## Results

### Patient population and demographics

In total, 108 participants were enrolled in B-Together (Arm 1, *n* = 55; Arm 2, *n* = 53) [8]. The biomarker analysis included 82 participants (Arm 1, *n* = 37; Arm 2, *n* = 45). Baseline demographics were generally comparable between Arms 1 and 2 (Table 1).

**Table 1:** Baseline demographics and disease characteristics for the analyzed B-Together subpopulation

	Arm 1 (*n* = 37)	Arm 2 (*n* = 45)	Overall (*N* = 82)
Sex, *n* (%)			
Female	8 (22)	17 (38)	25 (30)
Male	29 (78)	28 (62)	57 (70)
HBsAg, mean (SD), IU/mL	4,978.6 (9,456.5)	5,691.9 (9,005.6)	5,370.0 (9,161.2)
HBsAg ≤ 1000 IU/mL, *n* (%)	13 (35)	13 (29)	26 (32)
HBsAg > 1000 IU/mL, *n* (%)	24 (65)	32 (71)	56 (68)
HBV DNA < LLOQ^a^, *n* (%)	33 (89)	40 (89)	73 (89)
ALT, *n* (%)			
≤ ULN	33 (89)	37 (80)	70 (85)
> ULN	4 (11)	8 (18)	12 (15)

^a^LLOQ for HBV DNA = 20 IU/mL

*HBsAg* hepatitis B surface antigen, *HBV* hepatitis B virus, *LLOQ* lower limit of quantification, *ULN* upper limit of normal

### Baseline immunological heterogeneity observed between Arms 1 and 2

Proteomic analysis evaluated potential immunological and other disease-related differences at baseline (Week 1). Higher expression of 148 proteins was seen in a subset of Arm 2 versus Arm 1 participants (Fig. S1a and S1b). Subsequent pathway analysis of these proteins showed enrichment of apoptosis and immune response pathways (Fig. S1c). Figure S1d and S1e shows differences in expression levels of representative apoptosis and immune response-related proteins between Arms 1 and 2, respectively. Due to this baseline immunological heterogeneity between arms and any potential downstream effect on bepirovirsen’s pharmacodynamic response, subsequent biomarker analysis was performed and visualized within arms and virological response subgroups. Pooled analyses across arms are presented for completeness.

### Evidence of immune activation by Week 3, independent of virological response subgroup

**Fig. 2:**
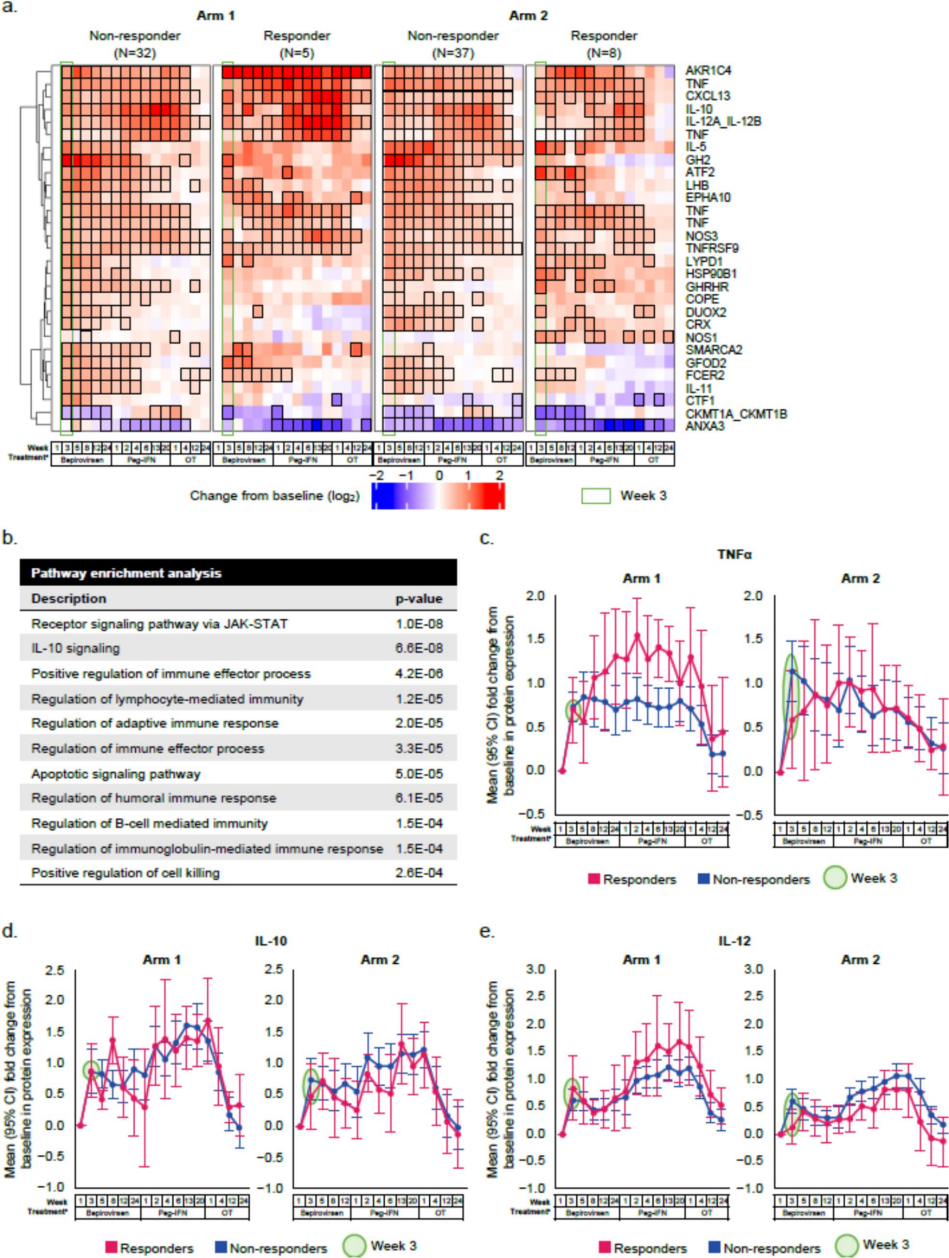
Longitudinal changes from baseline in mean relative expression of proteins found to be differentially expressed at Week 3 on bepirovirsen treatment (**a**). Top enriched pathways in the set of proteins showing differential expression (**b**). Longitudinal change from baseline in mean relative expression of TNFα (**c**), IL-10 (**d**), and IL-12 (**e**) on bepirovirsen treatment. **a** Red indicates an increase, blue a decrease. Black borders indicate statistically significant change relative to baseline (FDR ≤ 0.1; *p* values in Table S1). The four TNFs shown in the heatmap represent technical replicates. **b** Proteins differentially expressed at Week 3 in the heatmap in panel a were included in the pathway enrichment analysis. ^a^Samples taken pre-dose; Week 1 of the bepirovirsen treatment window is before the first bepirovirsen dose (baseline), Week 1 of the Peg-IFN window is before the first Peg-IFN dose, and Week 1 of the off-treatment window is after the last Peg-IFN dose. *BL* baseline, *FDR* false discovery rate, *IL* interleukin, *JAK-STAT* Janus kinase/signal transducers and activators of transcription, *OT* off-treatment, *Peg-IFN* pegylated interferon-α-2a, *TNF* tumor necrosis factor

To investigate biomarkers of bepirovirsen’s mechanism of action following treatment initiation, we focused on results at the earliest post-baseline timepoint available for the longitudinal proteomic analysis (Week 3). Bepirovirsen treatment led to a significant change from baseline (Week 1) in mean expression of 26 serum proteins, including cytokines. Increases were seen in the majority of proteins in both arms independent of virological response subgroup (Fig. 2a; Table S1). Immune effector responses (lymphocyte and humoral) and apoptotic pathways were among the top enriched pathways among the differentially expressed proteins (Fig. 2b). Within each arm, both responders and non-responders showed an increase from baseline in mean relative expression of key immune regulators, e.g., tumor necrosis factor (TNFα), interleukin (IL)-10, and IL-12, at Week 3 (Fig. 2c–e); the increase in these proteins appeared to be maintained during Peg-IFN treatment and declined in the off-treatment period. Pooled data across arms are presented in Fig. S2. These three cytokines can be a part of the innate immune response to drive activation of the adaptive immune response; TNFα and IL-12 are also involved in T-cell proliferation and survival [10, 11], and IL-10 can promote B-cell differentiation into antibody-producing plasmablasts [12].

Since B-Together did not have a placebo arm, we analyzed a subset of proteomic data from the B-Clear on-NA population comparing bepirovirsen (Arm 1) to placebo (Arm 4) [9], which confirmed that the results observed for TNFα-, IL-10-, and IL-12-related immune activation are bepirovirsen-specific (Fig. S3).

### Evidence of immune cell activation and proliferation by Week 5, independent of virological response subgroup

As ALT increases were observed between Weeks 5 and 12 post-bepirovirsen treatment initiation in some responders (Fig. S4), whole blood transcriptomic data from Week 5 explored changes in mRNA expression in peripheral immune cells prior to ALT peaks.

**Fig. 3:**
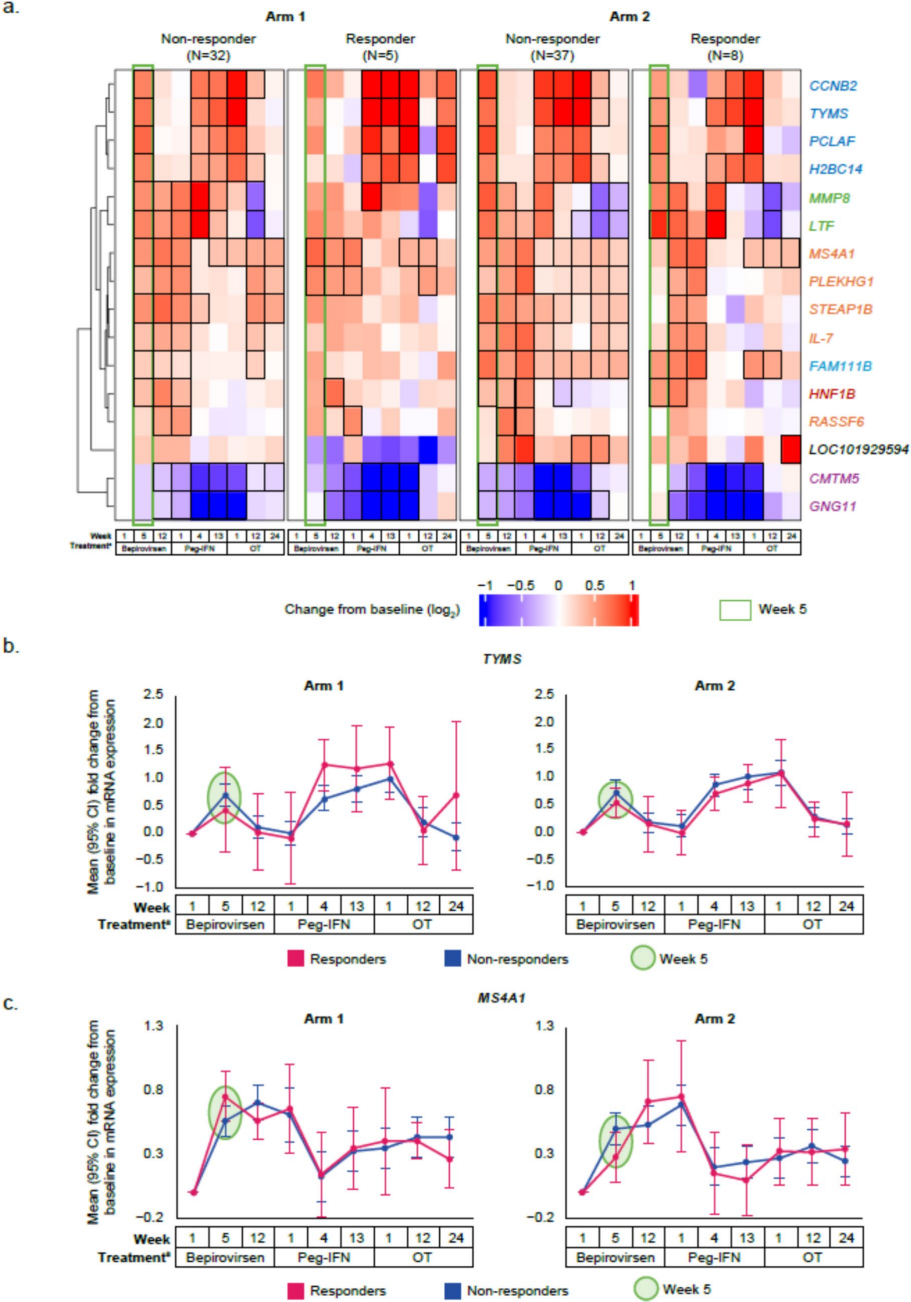
Longitudinal changes from baseline in mean expression of transcripts from whole blood found to be differentially expressed at Week 5 on bepirovirsen treatment (**a**). Longitudinal change from baseline in mean expression of *TYMS* (**b**) and *MS4A1* (**c**) on bepirovirsen treatment. **a** Red indicates an increase, blue a decrease. Black borders indicate statistically significant change. Transcripts within the green rectangle had significant changes at Week 5 relative to baseline (FDR ≤ 0.1; *p* values in Table S2). Transcript names colored in blue have proliferation functions; transcripts in green have innate immune response functions; transcripts in orange have B-cell functions; transcripts in purple have neutrophil-related functions; the transcript in dark red (HNF1B1) is a hepatocyte nuclear factor; the transcript in black has an uncharacterized function. ^a^Samples taken pre-dose; Week 1 of the bepirovirsen treatment window is before the first bepirovirsen dose (baseline), Week 1 of the Peg-IFN window is before the first Peg-IFN dose, and Week 1 of the off-treatment window is after the last Peg-IFN dose. *BL* baseline, *FDR* false discovery rate, *MS4A1* membrane spanning 4-domains A1, *OT* off-treatment, *Peg-IFN* pegylated interferon-α-2a, *TYMS* thymidylate synthetase

There was a significant increase in several transcripts associated with cell proliferation, including cyclin B2 (*CCNB2*) and H2B clustered histone 14 (*H2BC14*); DNA polymerase-associated transcripts (PCNA clamp-associated factor [*PCLAF*] and thymidylate synthetase [*TYMS*]); and the B-cell response pathway, including membrane spanning 4-domains A1 (*MS4A1*; *CD20*), IL-7, and pleckstrin homology and RhoGEF domain containing G1 (*PLEKHG1*), independent of treatment arms or virological response subgroup by Week 5 (Fig. 3a and Table S2). Increases in mean mRNA expression of *MS4A1*, a B-cell surface marker, and *TYMS*, an enzyme essential for DNA replication, are shown as illustrative examples in Fig. 3b and c. While increases in *MS4A1* expression appeared to be sustained throughout the bepirovirsen treatment phase, *TYMS* returned to baseline by Week 12. *TYMS* expression appeared to also increase during Peg-IFN treatment, followed by a return to baseline in the off-treatment phase.

In parallel with transcriptomic data, flow cytometry analysis showed upward trends in proliferation and activation of adaptive immune cells, including cytotoxic CD8^+^ T cells (CD38^+^; HLA-DR^+^) and B cells (Ki67^+^), by Week 5 independent of arm or virological response subgroup (Fig. S5). Representative flow cytometry plots illustrating the study gating strategy are shown in Fig. S6.

### Evidence of hepatocyte death associated with virological response by Week 8

**Fig. 4:**
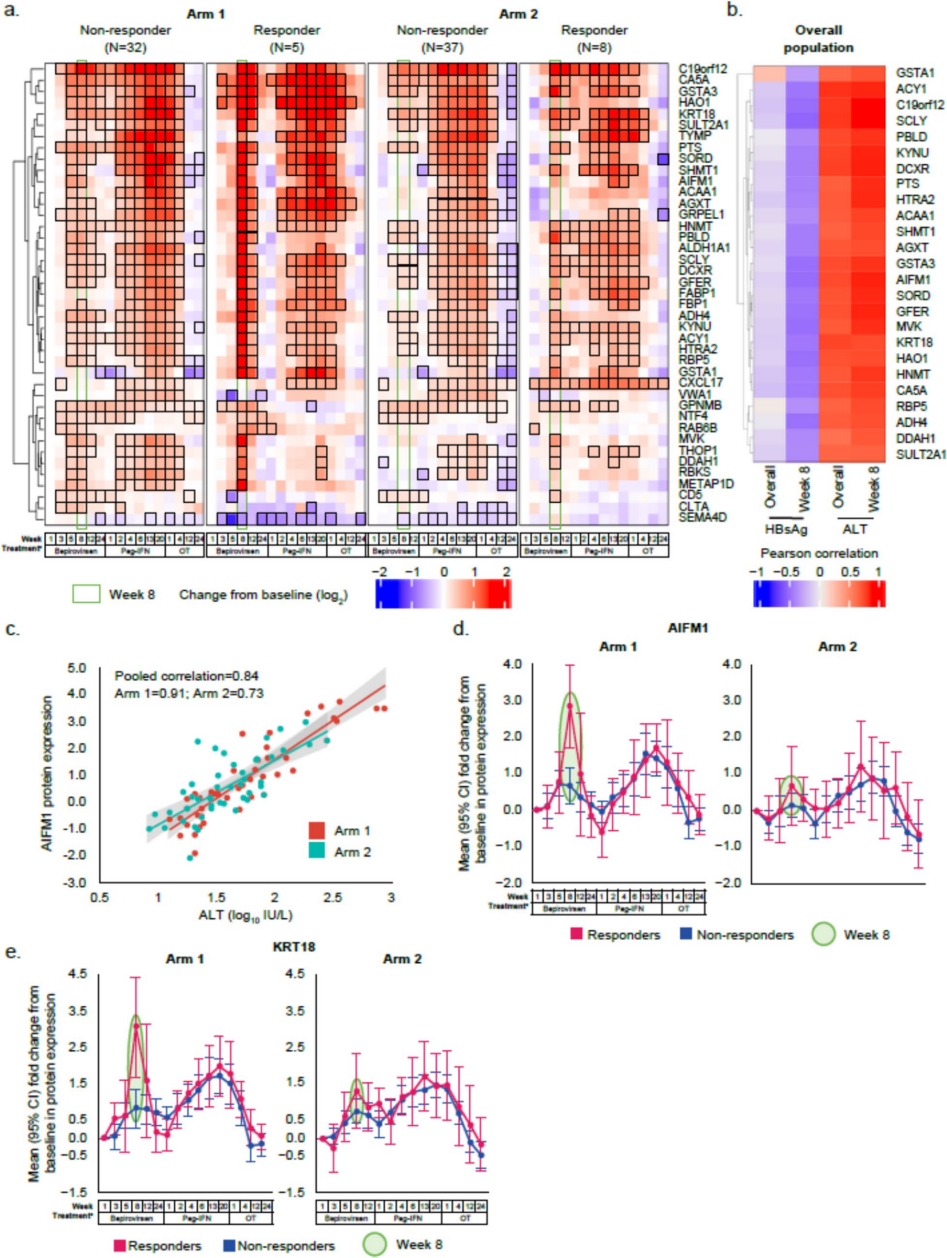
Longitudinal changes from baselines in mean relative expression of proteins found to be differentially expressed in responders versus non-responders at Week 8 on bepirovirsen treatment in Arms 1 and 2 (**a**). Correlation heatmap of apoptosis and liver-specific protein expression with ALT and HBsAg levels (**b**). Correlation between AIFM1 expression and ALT levels (**c**). Longitudinal change from baseline in mean relative expression of AIFM1 (**d**) and KRT18 (**e**) in responders versus non-responders for Arms 1 and 2. **a** Red indicates an increase, blue a decrease. Black borders indicate statistically significant change. Proteins within the green rectangle had significant changes at Week 8 relative to baseline (FDR ≤ 0.1; *p* values in Table S3). ^a^Samples taken pre-dose; Week 1 of the bepirovirsen treatment window is before the first bepirovirsen dose (baseline), Week 1 of the Peg-IFN window is before the first Peg-IFN dose, and Week 1 of the off-treatment window is after the last Peg-IFN dose. *AIFM1* apoptosis-inducing factor mitochondria associated 1, *ALT* alanine aminotransferase, *FDR* false discovery rate, *HBsAg* hepatitis B surface antigen, *KRT18* keratin type I cytoskeletal 18, *OT* off-treatment, *Peg-IFN* pegylated interferon-α-2a

To identify protein and pathway differences in responders and non-responders during bepirovirsen treatment, we examined proteomics data for significant differences at any timepoint between the two subgroups in both arms. However, the most prominent difference was observed at Week 8, when significantly higher expression of liver-specific proteins, including alcohol dehydrogenase 4 class II (ADH4), alanine-glyoxylate aminotransferase (AGXT), carbonic anhydrase 5A (CA5A), and apoptosis-specific proteins, such as apoptosis-inducing factor mitochondria associated 1 (AIFM1), keratin type I cytoskeletal 18 (KRT18), and HtrA serine peptidase 2 (HTRA2), was seen in responders versus non-responders across both treatment arms (Fig. 4a and Table S3). Next, we examined the correlation between the serum proteins most highly differentiated between responders and non-responders, with ALT and HBsAg levels. This correlation was evaluated both at Week 8 and overall (at all timepoints throughout the study). A trend in negative correlation was observed between these proteins and HBsAg at Week 8 only, and a strong positive association with ALT levels was observed both at Week 8 and overall (Fig. 4b). As an example, Fig. 4c shows a significant overall positive correlation (*r* = 0.84) between expression of the apoptosis marker AIFM1 and ALT at Week 8 in the study. Within arm correlation coefficients were 0.91 for Arm 1 and 0.73 for Arm 2. Overall, 20/25 proteins identified were mapped to the liver according to the String database (Fig. S7). Figure 4d, e, and Fig. S8 show illustrative line graphs of AIFM1, KRT18, ADH4, and CA5A, exhibiting significantly higher mean relative expression in responders than non-responders at Week 8 within each arm. This difference between responders and non-responders was less apparent when pooling AIFM1 and KRT18 data across arms, though a similar trend was observed (Fig. S9). The difference was also not apparent during Peg-IFN and off-treatment phases. Mean line graphs for the apoptosis (AIFM1, KRT18) and liver-specific markers (ADH4, CA5A) showed even greater differentiation between responders and null responders (Fig. S10). Furthermore, analysis of hematopoietic stem cells (HSC) or endothelial cells markers (CD34, THY-1, and PECAM1) showed no significant changes from baseline or between the response subgroups during bepirovirsen treatment (Fig. S11), supporting that there was no killing of HSCs or endothelial cells. Taken together, these findings suggest that observed ALT increases and surge of apoptosis and liver-specific proteins in the serum of responders at Week 8 are indicative of hepatocyte killing.

### Bepirovirsen treatment may potentiate killing of infected hepatocytes in responders

**Fig. 5:**
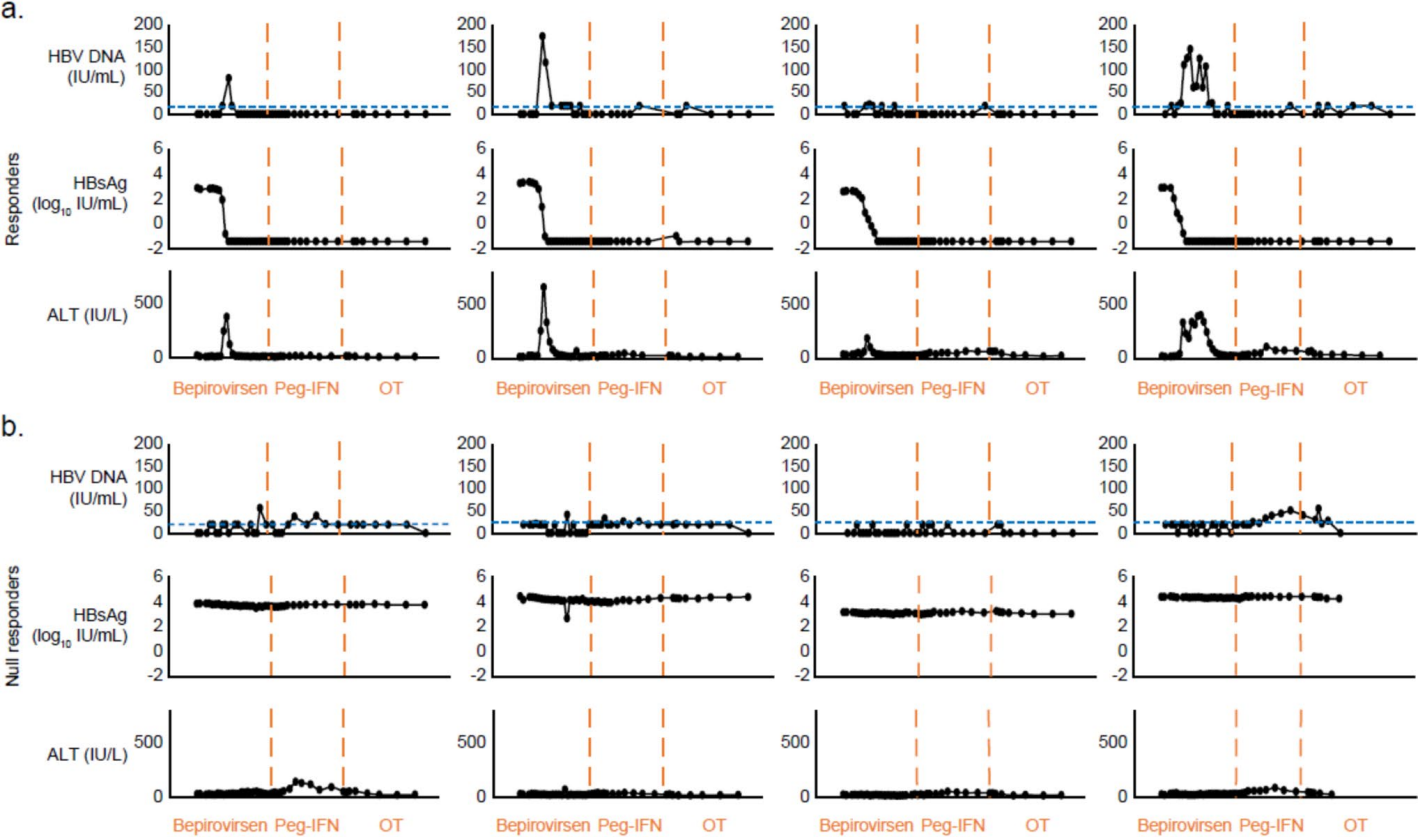
Kinetics of transient HBV DNA and ALT increases over time in responders (**a**) and null responders (**b**) from Arm 1. Responders were participants who achieved HBsAg and HBV DNA less than the LLOQ (HBsAg, < 0.05 IU/mL; HBV DNA, < 20 IU/mL) at each analysis window (end of bepirovirsen treatment; end of Peg-IFN treatment; off-treatment Weeks 8, 18, and 24) in the 24 weeks after the planned end of sequential treatment, without initiating new therapy to suppress viral replication; non-responders were participants who did not meet the primary outcome or participants who had missing data in the off-treatment period; null responders were participants who did not have two or more consecutive visits with a > 0.4 log_10_ reduction in HBsAg while on treatment. Participants remained on NA therapy throughout the B-Together study. The dotted blue line indicates the LLOQ for HBV DNA (20 IU/mL). *ALT* alanine aminotransferase, *HBsAg* hepatitis B surface antigen, *HBV* hepatitis B virus, *LLOQ* lower limit of quantification, *NA* nucleos(t)ide analog, *OT* off-treatment, *Peg-IFN* pegylated interferon-α-2a

To further probe the hypothesis that increased abundance of apoptosis markers in bepirovirsen responders is linked to productive killing of infected hepatocytes, we examined ALT, HBsAg, and HBV DNA kinetics. Figure 5 shows examples of individual HBsAg, HBV DNA, and ALT profiles from responders and null responders in Arm 1, who received the 24-week bepirovirsen dosing regimen, identical to ongoing Phase 3 studies (NCT05630820 and NCT05630807). Kinetics of HBV DNA increases in individual responder profiles appeared to mirror ALT profiles on bepirovirsen treatment, indicating that the increase in these two readouts may be the outcome of the same underlying molecular mechanism of infected hepatocyte death. Conversely, in null responders, ALT and HBV DNA increases, as well as HBsAg decreases, were not prominent (Fig. 5b), suggesting that these biomarkers are indirect indicators of therapeutic killing of infected hepatocytes leading to virological response in a subset of responders.

## Discussion

While this post hoc analysis of peripheral biomarker data from the B-Together study was not specifically designed to elucidate bepirovirsen’s innate immune mechanism of action, it provides novel insights into bepirovirsen’s immune effects using a multimodal approach. Taken together, the proteomic, transcriptomic, and PBMC biomarker findings demonstrate that bepirovirsen has an immunostimulatory pharmacodynamic effect and support the hypothesis that bepirovirsen activates immune responses to promote killing of infected hepatocytes, leading to virological response.

Bepirovirsen stimulates production of key components associated with both innate and adaptive immune responses. Bepirovirsen-treated participants, regardless of arm or virological response subgroup, showed an increase in mean relative expression of cytokines and immune-related proteins at Week 3, including TNFα, IL-10, and IL-12, all of which can function as part of the innate, as well as adaptive, immune response. This increase in cytokines and immune-related proteins expression at Week 3 was also observed in the pooled analysis across arms. Post hoc analyses from B-Clear indicated this increase in cytokine and immune-related protein expression is a bepirovirsen-induced effect, as no such increase was observed in placebo-treated participants. TNFα and IL-12 are pro-inflammatory cytokines that play a critical role in HBV infection through activation of cytotoxic CD8^+^ T cells, which are considered the main adaptive immune effectors of viral clearance in HBV infection [13–15]. In addition to the known immunosuppressive nature of the cytokine IL-10 [16], it can promote adaptive immune responses and has been shown to be important for B-cell differentiation into plasmablasts and immunoglobulin (Ig) G and IgM antibody production [12, 16]. At Week 5 of bepirovirsen treatment, there was a significant increase in transcripts associated with cell proliferation (*TYMS*, *CCNB2*, *H2BC14*) [17–19] and B-cell responses (*MS4A1*, *IL-7*, *PLEKGH1*) [20–22]. Since whole blood transcriptomic changes are largely driven by changes in immune cells [23], this suggests immune cell proliferation. In alignment with transcriptomic data, we also observed trends in proliferation and activation of adaptive CD8^+^ cytotoxic T cells and B cells. Collectively, proteomic, whole blood transcriptomic, and cellular data suggest that bepirovirsen appear to induce immune activation independent of virological response. Additionally, while *MS4A1* transcripts and B-cell levels remained high throughout the bepirovirsen treatment phase, increases in *TYMS* and CD8^+^ T cells returned to baseline by Week 12, similar to the ALT trajectory in responders. This may suggest a link between activated CD8^+^ T cells and ALT increases. However, it is apparent that immune activation is not the sole determinant of virological response to bepirovirsen. Instead, it appears to be a multifactorial effect including, but not limited to, known factors like baseline HBsAg [9], type of NA treatment [8, 9], and other yet to be determined factors, along with immune activation.

Indirect evidence that bepirovirsen treatment promotes hepatocyte apoptosis in responders was observed at Week 8. Hepatocytes are mitochondria-rich cells, and hepatic injury leads to increased mitochondrial enzymes in serum [24]. Here, the simultaneous increase in ALT expression, a marker of liver injury, alongside accumulation of apoptosis-specific markers like AIFM1, KRT18, and HTRA2, and liver-specific proteins like ADH4, CA5A, and AGXT, some of which are mitochondria-specific, in the serum of responders was suggestive of hepatocyte killing. Notably, increases in ALT, AIFM1, KRT18, ADH4, and CA5A expression were more pronounced in responders than non-responders and nearly absent in null responders, supporting the hypothesis that this is a bepirovirsen treatment effect. This differential expression between responder and non-responders at Week 8 was not as prominent in the pooled analysis, probably due to baseline heterogeneity, emphasizing the importance of arm-specific analysis. The synchronous pattern of transient HBV DNA and ALT increases alongside HBsAg decline, observed in the serum of some responders and absent from some null responders, provides additional evidence that bepirovirsen may mediate killing of HBV-infected hepatocytes, leading to virological response. Although not conclusive, these data, which align with findings from two previous studies [25, 26], do not suggest indiscriminate killing of hepatocytes. Nishio et al. demonstrated that Peg-IFN-induced apoptosis of HBV-infected hepatocytes is associated with concurrent elevations of ALT, caspase-cleaved KRT18, and HBV DNA and subsequent decline in HBsAg [26]. Furthermore, Anastasiou et al. reported that patients coinfected with HBV and hepatitis D virus who experienced transient HBV DNA increases while receiving Peg-IFN had greater ALT and KRT18 increases and were more likely to achieve HBsAg loss than patients who did not have transient HBV DNA increases [25]. These observations suggest that, during bepirovirsen treatment, transient increases in serum HBV DNA are indicative of infected hepatocyte killing; in this context, hepatocyte death and ALT increases may be therapeutic in nature and could act as markers of treatment efficacy as opposed to drug-induced liver injury or virological relapse.

One unexpected observation in this study was the more pronounced elevation of ALT, AIFM1, and KRT18 in Arm 1 than Arm 2 responders, despite both arms having received the same number of bepirovirsen doses by Week 8. However, as 148 immune response and apoptosis-related proteins were more highly expressed in Arm 2 than Arm 1 at baseline, Arm 2 responders could appear to have a lower change from baseline in apoptosis and liver-specific proteins than Arm 1 responders. This suggests that baseline immunological status may influence response to bepirovirsen.

These analyses were opportunistic and not powered. Therefore, although we believe that important insights and hypotheses can be gained and generated from these results, several limitations should be considered. First, considering heterogeneity in this disease setting, generalizability is limited, and further evidence is required to make a conclusive statement. Second, all patients in B-Together received bepirovirsen, and findings could not be validated against a placebo-treated population within this study. Third, due to limited PBMC availability, this study could not demonstrate activation of HBV-specific T and B cells in the liver or periphery. Furthermore, owing to lack of acute timepoints, bepirovirsen’s effect on innate immune mechanisms, as previously hypothesized [5, 7], may not be well captured [27]. Finally, due to operational complexities, this study did not include liver sampling, which would have been required to show direct evidence of killing of infected hepatocytes. However, peripheral biomarker data provide indirect correlative evidence of this finding, which is also supported by previous studies linking peripheral HBV DNA increases with killing of infected hepatocytes and positive virological outcome [25, 26].

The following conclusions were drawn from this analysis: bepirovirsen appears to induce an immune response, as evidenced by increased cytokine levels and activation/proliferation of immune cells; these findings continue to support the conclusion that ALT increases are more frequent in responders than non-responders, as previously observed [8], and are associated with markers of hepatocyte death; the results verify ALT increases as a marker of therapeutic response, as they are associated with HBsAg decline; hepatocyte death appears to include HBV-infected hepatocytes as evidenced by concurrent transient increase in HBV DNA and ALT, which may be a key mechanism of action of bepirovirsen. Conclusions should be confirmed in a larger study.

## Supplementary Information

Below is the link to the electronic supplementary material.

Supplementary file1 (DOCX 3491 KB)

## Data Availability

Please refer to GSK’s weblink to access GSK’s data sharing policies and as applicable seek anonymized subject level data via the link https://www.gsk-studyregister.com/en/.
