# Supplementary material for: Immunomodulation by bepirovirsen may induce killing of infected hepatocytes (B-Together study)
Source: Hepatol Int. 2025 Oct 3;20(1):46–58. doi: 10.1007/s12072-025-10917-0 (PMC12923464; doi:10.1007/s12072-025-10917-0)
Supplement: Supplementary file 1 — Supplementary file1 (DOCX 3491 KB) [file 12072_2025_10917_MOESM1_ESM.docx]

**Immunomodulation by bepirovirsen may induce killing of infected hepatocytes (B-Together study)**

Shilpy Joshi^1*^; Johannes Freudenberg^2^; Jennifer M. Singh^1^; William T. Jordan^2^; Leigh Felton^3^; Susan Dixon^3^; Melanie Paff^4^; Dickens Theodore^5^; Jill Walker^6^

*^1^Clinical Biomarkers & Correlative Sciences, Precision Medicine, GSK, Collegeville, PA, USA; ^2^Translational Omics, GSK, Collegeville, PA, USA; ^3^Clinical Development, GSK, London, UK; ^4^Clinical Development, GSK, Collegeville, PA, USA; ^5^Clinical Research, GSK, Research Triangle Park, NC, USA; ^6^Clinical Biomarkers, GSK, San Francisco, CA, USA*

***Corresponding author:**

Name: Shilpy Joshi

Address: GSK, Collegeville, PA, USA

Phone: +16109174224

Email: shilpy.s.joshi@gsk.com

**Table of Contents**

[Supplementary Methods 4](#_Toc205822774)

[B-Together study design and patient population 4](#_Toc205822775)

[B-Clear study design and patient population 4](#_Toc205822776)

[B-Clear response subgroups 5](#_Toc205822777)

[B-Together biomarker analyses 5](#_Toc205822778)

[HBV DNA and HBsAg quantification 7](#_Toc205822779)

[Pathway analysis 7](#_Toc205822780)

[B-Clear serum TNFα, IL-10, and IL-12 analysis 8](#_Toc205822781)

[Supplementary Figures 9](#_Toc205822782)

[Fig. S1. 148 proteins showed differential expression at baseline between Arms 1 and 2 (a). Volcano plot showing differential protein expression at baseline between Arms 1 and 2 (b). Top ten pathways overrepresented in the set of 148 proteins in Arm 2 (c). Examples of apoptotic signaling pathways (d) and immune response–associated proteins (e) with an imbalance in expression at baseline 9](#_Toc205822783)

[Fig. S2. Mean TNFα (a), IL-10 (b), and IL-12 (c) protein expression (pooled data across treatment arms) 11](#_Toc205822784)

[Fig. S3. Change from baseline in TNFα (a), IL-10 (b), and IL-12 (c) at Week 4 following bepirovirsen treatment in Arm 1 and placebo treatment in Arm 4 (B-Clear supportive analysis) 13](#_Toc205822785)

[Fig. S4. Change from baseline in ALT protein levels following bepirovirsen treatment in responders and null responders 15](#_Toc205822786)

[Fig. S5. Activation and proliferation of cytotoxic CD8^+^CD38^+^HLA-DR^+^ T cells at Week 5 post-bepirovirsen treatment initiation (a). Proliferation of Ki67^+^ B cells at Week 5 post-bepirovirsen treatment initiation (b) 16](#_Toc205822787)

[Fig S6. Study specific gating strategy was applied to all samples to identify HLADR+CD38+ CD8+ T cells at (a) baseline, (b) at Week 5 Day 29 post dose and Ki67+ B cells at (c) baseline, (d) at Week 5 Day 29 post dose 17](#_Toc205822788)

[Fig. S7. String analysis of the top 25 proteins most correlated with ALT levels 18](#_Toc205822789)

[Fig. S8. Change from baseline in mean ADH4 (a) and CA5A (b) protein expression in responders versus non-responders for Arms 1 and 2 20](#_Toc205822790)

[Fig. S9. Change from baseline in mean AIFM1 (a) and KRT18 (b) protein expression in responders versus non-responders (pooled data across treatment arms) 22](#_Toc205822791)

[Fig. S10. Mean AIFM1 (a), KRT18 (b), ADH4 (c), and CA5A (d) protein expression in responders versus null responders for Arms 1 and 2 at Week 8 24](#_Toc205822792)

[Fig. S11. Change from baseline in mean CD34 (a) THY-1 (b)and PECAM1 (c) protein expression in responders versus non-responders for Arms 1 and 2 26](#_Toc205822793)

[Supplementary Tables 28](#_Toc205822794)

[Table S1. P-values for the analysis of change from baseline in protein expression in Arm 1 non-responders (a), Arm 1 responders (b), Arm 2 non-responders (c) and Arm 2 responders (d) 28](#_Toc205822795)

[Table S2. P-values for the analysis of change from baseline in gene expression in Arm 1 non-responders (a), Arm 1 responders (b), Arm 2 non-responders (c) and Arm 2 responders (d) 34](#_Toc205822796)

[Table S3. P-values for the analysis of change from baseline in protein expression in Arm 1 non-responders (a), Arm 1 responders (b), Arm 2 non-responders (c) and Arm 2 responders (d) 39](#_Toc205822797)

[R code 47](#_Toc205822798)

[Load libraries and previously compiled functions & data 47](#_Toc205822799)

[Table 1 47](#_Toc205822800)

[Figure 2B 49](#_Toc205822801)

[Figure S1 c, d 50](#_Toc205822802)

[Figure 2C-E 51](#_Toc205822803)

[Figure 4B 52](#_Toc205822804)

[Figure 4C 55](#_Toc205822805)

[Figure 4D-E 55](#_Toc205822806)

[Figure 5 57](#_Toc205822807)

[Suppl Figure 4 59](#_Toc205822808)

[Suppl Figure 5 59](#_Toc205822809)

[Suppl Figure 8 61](#_Toc205822810)

[Suppl Figure 10 62](#_Toc205822811)

[Load microarray data 63](#_Toc205822812)

[Figure 3A 63](#_Toc205822813)

[Figure 3B-C 65](#_Toc205822814)

[Load data 66](#_Toc205822815)

[Figure 2A 66](#_Toc205822816)

[Figure 4A 68](#_Toc205822817)

[References 71](#_Toc205822818)

## Supplementary Methods

### **B-Together study design and patient population**

In B-Together, eligible participants were adults (18–75 years of age) who were HBsAg positive for ≥6 months prior to screening, on stable NA therapy, had HBsAg >100 IU/mL, HBV DNA <90 IU/mL, ALT ≤2 times the ULN, and were eligible at screening for Peg-IFN treatment [[1](#_ENREF_1)]. Key exclusion criteria included co-infection with hepatitis C, hepatitis D, or HIV, cirrhosis, and HCC.

### **B-Clear study design and patient population**

A set of data from B-Clear was analyzed post hoc to support findings from the B-Together study. Recruitment sites for both studies have been published previously; 73.5% of them were the same [[1](#_ENREF_1), [2](#_ENREF_2)].

B-Clear was a multicenter, randomized Phase 2b study in participants with chronic HBV infection [[2](#_ENREF_2)]. On-NA participants were randomized 3:3:3:1 to receive bepirovirsen 300 mg weekly with loading dose (LD) for 24 weeks (Arm 1), bepirovirsen 300 mg for 12 weeks with LD followed by 150 mg for 12 weeks (Arm 2), bepirovirsen 300 mg for 12 weeks with LD followed by placebo for 12 weeks (Arm 3), or placebo for 12 weeks followed by bepirovirsen 300 mg without LD for 12 weeks (Arm 4). Additional details of the study design have been published previously [[2](#_ENREF_2), [3](#_ENREF_3)].

Eligibility criteria for the B-Together and B-Clear on-NA populations were identical except the B-Together participants additionally had to meet Peg-IFN eligibility criteria.

### **B-Clear response subgroups**

In B-Clear, responders were participants who achieved the primary outcome: HBsAg and HBV DNA less than the LLOQ (HBsAg, <0.05 IU/mL; HBV DNA, <20 IU/mL) maintained for 24 weeks after the planned end of bepirovirsen treatment, without initiating new treatment to suppress viral replication. Non-responders were participants who did not meet the primary outcome.

### **B-Together biomarker analyses**

#### Sample collection

The same biomarker collection schedule applied to participants who were immediately eligible for Peg-IFN treatment at bepirovirsen EoT and to those who became eligible for Peg-IFN within 12 weeks after EoT (Peg-IFN eligibility criteria for the B-Together study have been published elsewhere [[1](#_ENREF_1)]).

#### Flow cytometry

A 30-color deep immunophenotyping spectral flow cytometry panel was designed and validated according to H62 Clinical and Laboratory Standards Institute guidelines [[4](#_ENREF_4)] to define various cell types including T cells, B cells, natural killer cells, and mucosal-associated invariant T cells in cryopreserved PBMCs from participants with chronic HBV infection. The panel was run using a Cytek Aurora (5 laser; Cytek Biosciences, Fremont, California, USA). The expression of a variety of activation/exhaustion markers, including but not limited to cluster of differentiation 38 (CD38), human leukocyte antigen – DR isotype (HLA-DR) and Ki67, was also measured for evaluation of proliferative activity. Other markers could not be analyzed due to low sample size. Gating was performed in OMIQ (Dotmatics, Boston, Massachusetts, USA). As quality of the cryopreserved PBMCs varied across sites, the threshold for reporting data was set as an event number of 10,000 CD3^+^ T cells acquired per sample.

#### Whole blood transcriptomics

Whole blood transcriptomics was performed on blood samples using the Clariom S Assay HT (Thermo Fisher Scientific; Dublin, Ireland). Epistem (Manchester, UK) provided Clariom S Assay HT raw data files (“CEL files”) for each sample (i.e., participant visit). Raw data files were pre-processed using RMA [[5](#_ENREF_5)] and Entrez gene-based probe annotation definitions [[6](#_ENREF_6)]. Principal component analysis was performed to determine if batch correction was necessary, and if it was, the ComBat R package [[7](#_ENREF_7)] was applied to correct the pre-processed data for batch effects. The resulting expression values after the pre-processing step were on the log_2_ scale and used for downstream analyses.

#### Serum proteomics

Serum proteomic profiling was conducted using the Olink 1536 Explore platform (Olink, Uppsala, Sweden; https://olink.com/). The resulting proteomic expression profiles were represented as normalized protein expression (NPX) values. NPX values were reported on the log_2_ scale together with the limit of detection (LOD) and a quality control (QC) warning flag where applicable. To identify samples with QC warnings not suitable for further analysis, principal component analysis was performed on each of the four subpanels, and the clustering of samples with QC warnings was compared with that of samples which passed QC [[8](#_ENREF_8)]. Samples with QC warnings that consistently (i.e., in all four subpanels) clustered separately from the samples that passed QC were removed from further analysis [[8](#_ENREF_8)]. Proteins with NPX >LOD for 20% or less of the samples were removed unless the samples with NPX <LOD were clearly associated with a specific sample subset (e.g., treatment group, time point, response type, etc.).

### **HBV DNA and HBsAg quantification**

The COBAS HBsAg II quant II (Roche) and COBAS AmpliPrep/COBAS TaqMan HBV Test, v2.0 (Roche) were used as per manufacturer’s instructions to quantify HBsAg (IU/mL) and HBV DNA (IU/mL).

### **Pathway analysis**

Pathway analysis was conducted using a method based on the hypergeometric test [[9](#_ENREF_9)], and the entire genome was used as the background for the analysis. The packages used were GO.db R package version 3.8.2 (Bioconductor) [[10](#_ENREF_10)] and reactome.db R package version 1.68.0 (Bioconductor) [[11](#_ENREF_11)]. String analysis was performed using default settings [[12](#_ENREF_12)]. The Benjamini–Hochberg method to estimate the false discovery rate (FDR) was used to account for testing thousands of hypotheses simultaneously. FDR is also frequently referred to as “q-value” or “adjusted p-value” [[13](#_ENREF_13)]. Unless otherwise stated, a significance threshold of adjusted p-value ≤0.1 was used. For differential protein and transcript expression, an absolute fold change >1.5 and FDR ≤0.1 was used in at least one of the subgroups within the arms. Time points were treated independently.

### **B-Clear serum TNFα, IL-10, and IL-12 analysis**

Serum samples from participants on NA therapy in B-Clear Arm 1 (bepirovirsen 300 mg weekly for 24 weeks) and Arm 4 (placebo for 12 weeks followed by bepirovirsen 300 mg for 12 weeks) were assessed for TNFα, IL-10, and IL-12 proteins at baseline and Week 4. These arms were selected to investigate specificity of immune activation by bepirovirsen in comparison to placebo treatment on a background of NA therapy, as observed in B-Together at Week 3.

## Supplementary Figures

### **Fig. S1. 148 proteins showed differential expression at baseline between Arms 1 and 2 (a). Volcano plot showing differential protein expression at baseline between Arms 1 and 2 (b). Top ten pathways overrepresented in the set of 148 proteins in Arm 2 (c). Examples of apoptotic signaling pathways (d) and immune response–associated proteins (e) with an imbalance in expression at baseline**


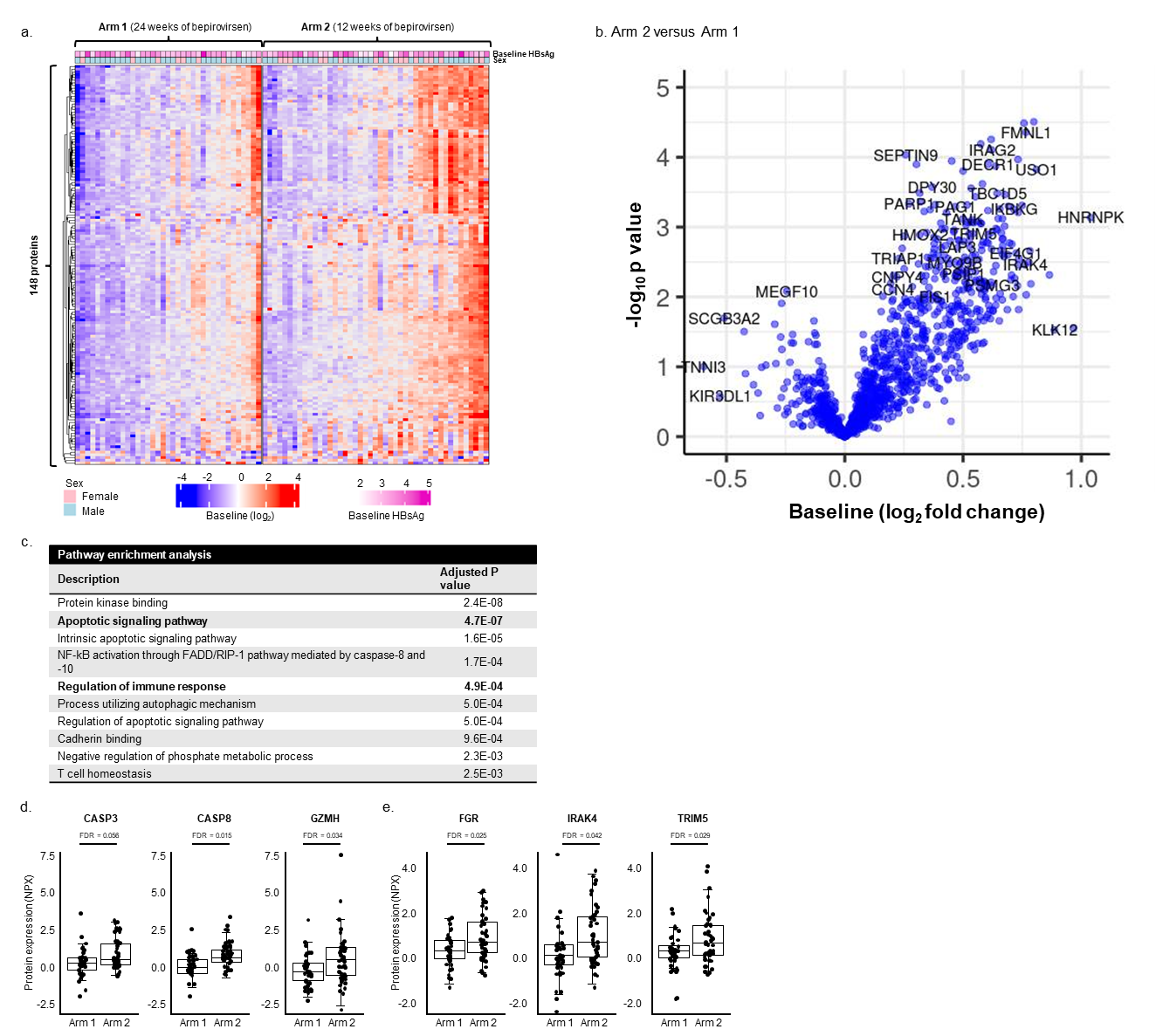


List of proteins shown in Figure 2A heatmap (FDR ≤0.1, from top to bottom): GYS1, STX6, NFATC1, AXIN1, GRAP2, CDKN1A, LAT2, ERBIN, CALCOCO1, EIF4G1, TBCB, CLIP2, BANK1, ITGB1BP2, CNST, INPPL1, GOPC, KIFBP, MAP4K5, CASP3, EBAG9, MITD1, RHOC, PPP1R9B, CD69, SKAP2, CORO1A, FYB1, PTPN1, DNAJA2, ARHGEF12, CA13, DOK2, SRC, MAVS, TACC3, SNAP29, SCAMP3, CRKL, DBNL, CDKN2D, MAP2K6, FKBP1B, ATP5IF1, DIABLO, TBC1D5, IRAG2, CRADD, CIAPIN1, CC2D1A, STX4, TRIM5, IQGAP2, HSPB1, BAX, AKR1B1, PDP1, SORD, DECR1, FGR, FMNL1, NPM1, SRP14, MAD1L1, ELOA, SKAP1, FOXO3, SUGT1, TRAF2, TBC1D17, PDCD5, SOD1, PPCDC, ANKRD54, DNAJB1, AKT1S1, RABGAP1L, CD2AP, GMPR, CARHSP1, AARSD1, EIF4EBP1, PSMG3, DNPH1, PSMD9, AK1, PPME1, CA2, ATG4A, RILP, HAGH, FHIT, NSFL1C, IRAK4, NT5C3A, SIRT2, PEBP1, PARK7, MIF, VTA1, PLPBP, RWDD1, STAMBP, TBCC, HEXIM1, METAP2, DCTN1, APRT, DARS1, PPP1R12A, ICA1, FADD, BCR, USP8, IKBKG, COMT, TDRKH, FABP5, RNF41, DFFA, NUB1, CASP8, ABHD14B, ENO1, DCTN2, MGMT, TXLNA, USO1, DDX58, HNRNPK, STAT5B, TPMT, HDGF, SERPINB1, LBR, ANXA3, S100A12, PADI4, NCF2, MNDA, APEX1, IPCEF1, RASSF2, HAO1, ACAA1, LEP, GZMH, PSPN. Panel c: Pathways of interest have been highlighted in bold, and examples of these pathways are shown in panels d and e. *The p-values resulting from Fisher’s test were adjusted for multiple testing using the Benjamini–Hochberg method.

CASP, caspase; FADD, FAS-associated death domain protein; FDR, false discover rate; FGR, Gardner-Rasheed feline sarcoma; GZMH, granzyme H; IRAK4, interleukin-1 receptor-associated kinase 4; RIP-1, receptor interacting protein 1; TRIM5, tripartite motif-containing protein 5.

### **Fig. S2. Mean TNFα (a), IL-10 (b), and IL-12 (c) protein expression (pooled data across treatment arms)**


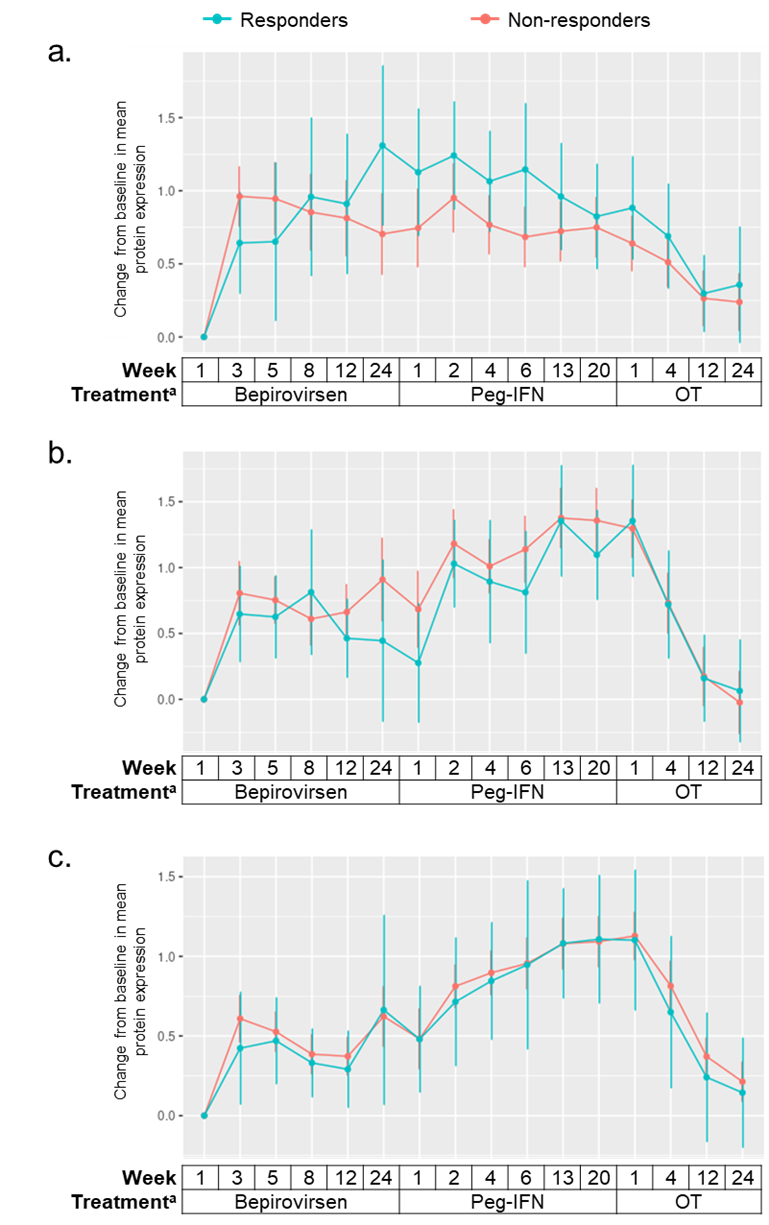


^a^Samples taken pre-dose; Week 1 of the bepirovirsen treatment window is before the first bepirovirsen dose (baseline), Week 1 of the Peg-IFN window is before the first Peg-IFN dose, and Week 1 of the off-treatment window is after the last Peg-IFN dose.

IL, interleukin; OT, off-treatment; Peg-IFN, pegylated interferon-α-2a; TNF, tumor necrosis factor.

### **Fig. S3. Change from baseline in TNFα (a), IL-10 (b), and IL-12 (c) at Week 4 following bepirovirsen treatment in Arm 1 and placebo treatment in Arm 4 (B-Clear supportive analysis)**


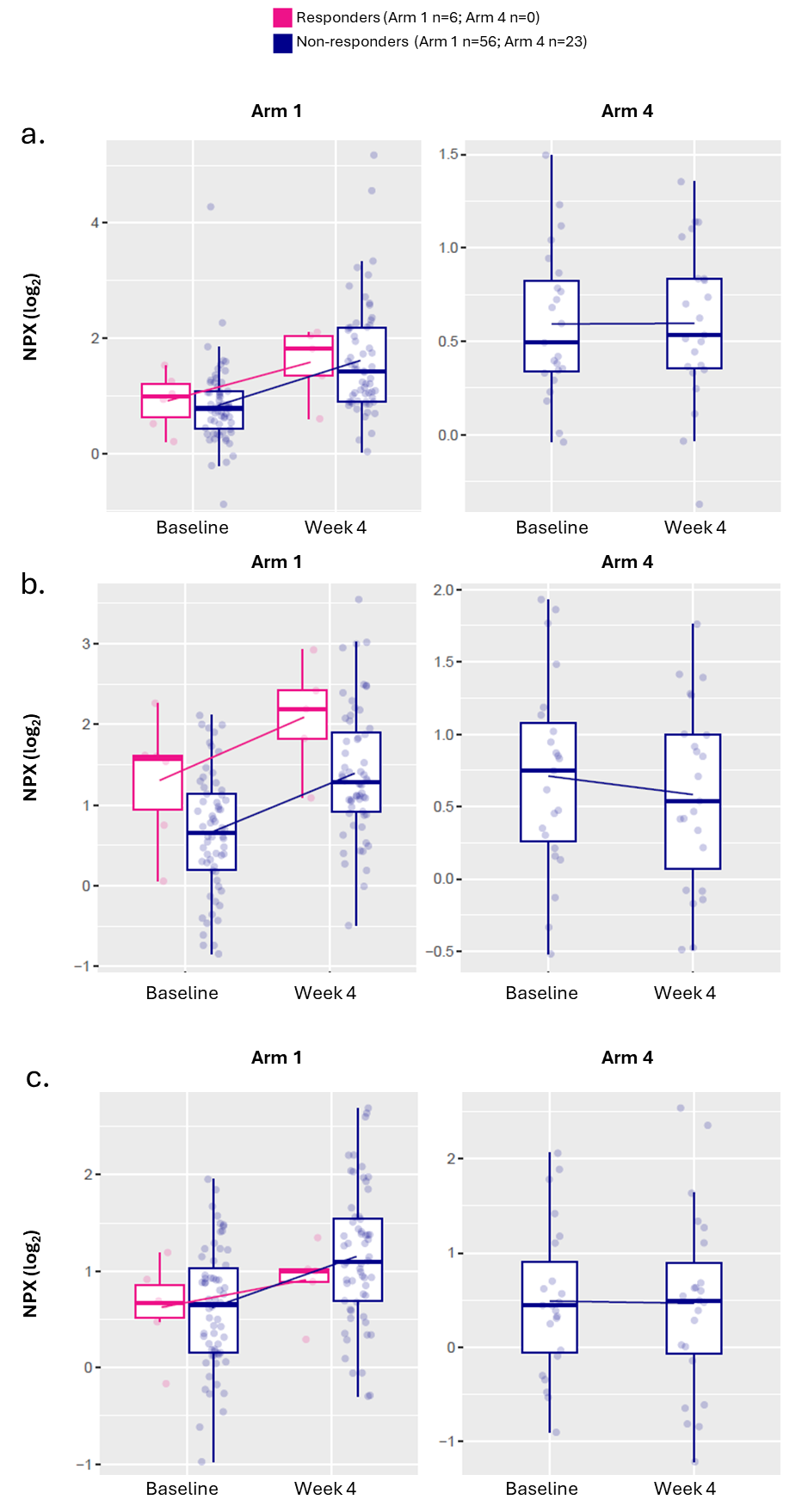


Panels show participants from Arm 1 (bepirovirsen 300 mg weekly with LD for 24 weeks) and Arm 4 (placebo for 12 weeks followed by bepirovirsen 300 mg weekly without LD for 12 weeks) in B-Clear.

LD, loading dose**.**

### **Fig. S4. Change from baseline in ALT protein levels following bepirovirsen treatment in responders and null responders**


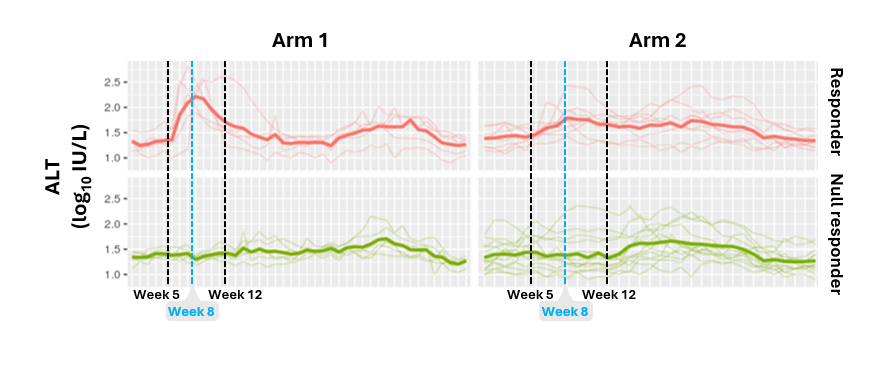


Panels show participants from Arm 1 (bepirovirsen 300 mg weekly + LD for 24 weeks followed by up to 24 weeks of Peg-IFN) and Arm 2 (bepirovirsen 300 mg weekly + LD for 12 weeks followed by up to 24 weeks of Peg-IFN) with proteomic data in B-Together.

ALT, alanine aminotransferase; LD, loading dose; Peg-IFN, pegylated interferon-α-2a.

### **Fig. S5. Activation and proliferation of cytotoxic CD8^+^CD38^+^HLA-DR^+^ T cells at Week 5 post-bepirovirsen treatment initiation (a). Proliferation of Ki67^+^ B cells at Week 5 post-bepirovirsen treatment initiation (b)**


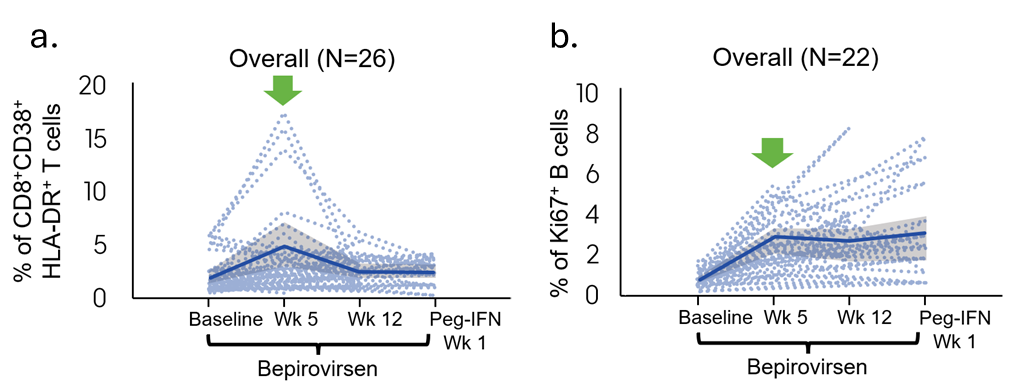


Overall contains both arms. The dark blue line is the mean, the dotted lines represent individual participant data, and the shaded area represents the 95% CI. The green arrow indicates Week 5.

HLA-DR, human leukocyte antigen – DR isotype; CD, cluster of differentiation; Peg-IFN, pegylated interferon-α-2a; wk, week.

### **Fig S6. Study-specific gating strategy was applied to all samples to identify** **HLADR+CD38+ CD8+ T cells at (a) baseline, (b) at Week 5 Day 29 post dose and Ki67+ B cells at (c) baseline, (d) at Week 5 Day 29 post dose**


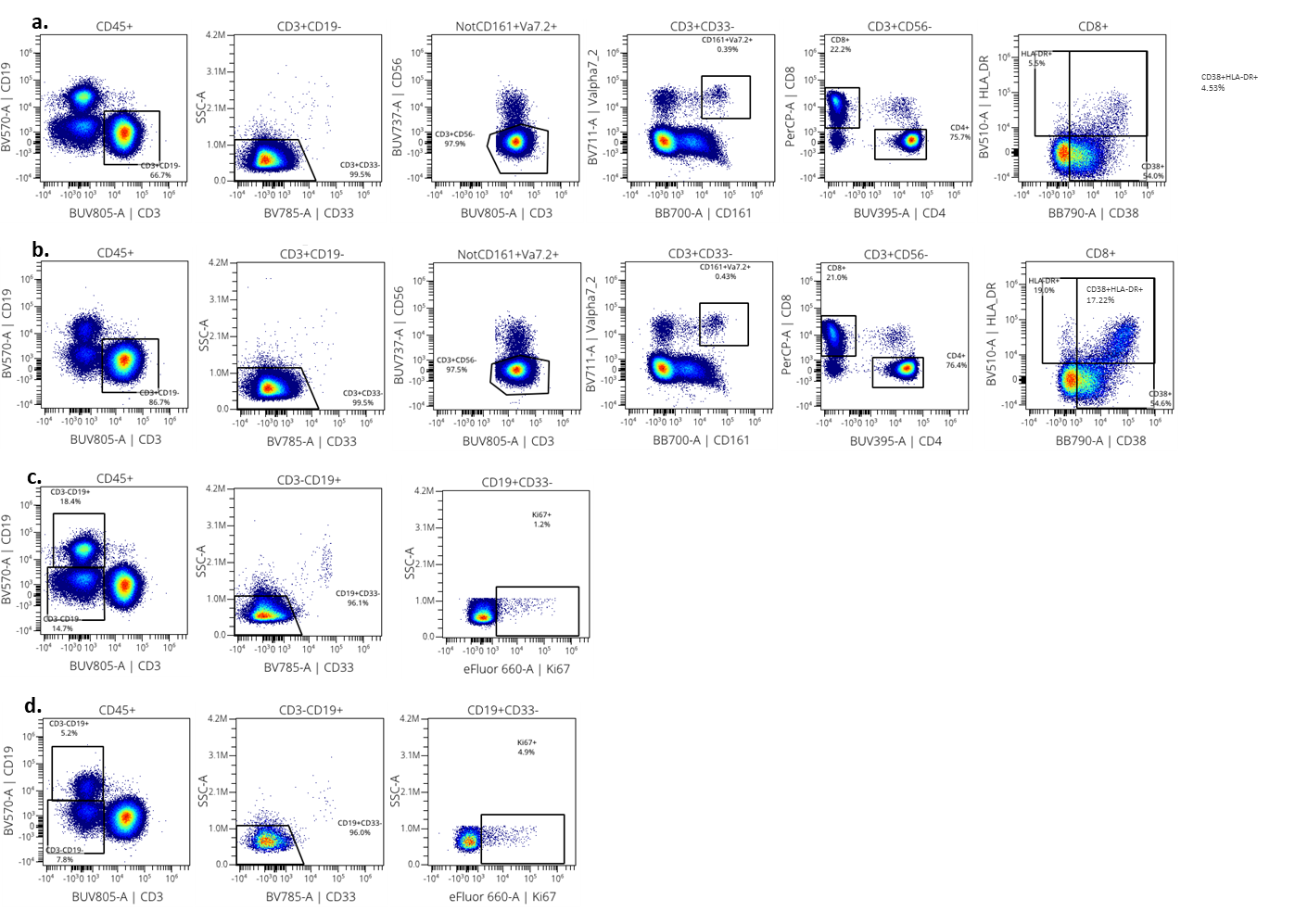


Representative graphs to illustrate the identification of A: HLADR+CD38+ CD8+ T cells at baseline, B: HLADR+CD38+ CD8+ T cells at Week 5 Day 29 post dose, C: Ki67+ B cells at baseline, D: Ki67+ B cells at Week 5 Day 29 post dose. The following clean-up gates were applied immediately before the CD3 v CD19 bivariate dot plot - Time/Cells/Singlets/Live/CD45+. The Cells gate was determined based on the FSC/SSC profile. Singlets were defined using FSC-A v FSC-H; Live cells were defined as such due to their negativity for a fixable viability probe.

### **Fig. S7.** **String analysis of the top 25 proteins most correlated with ALT levels**


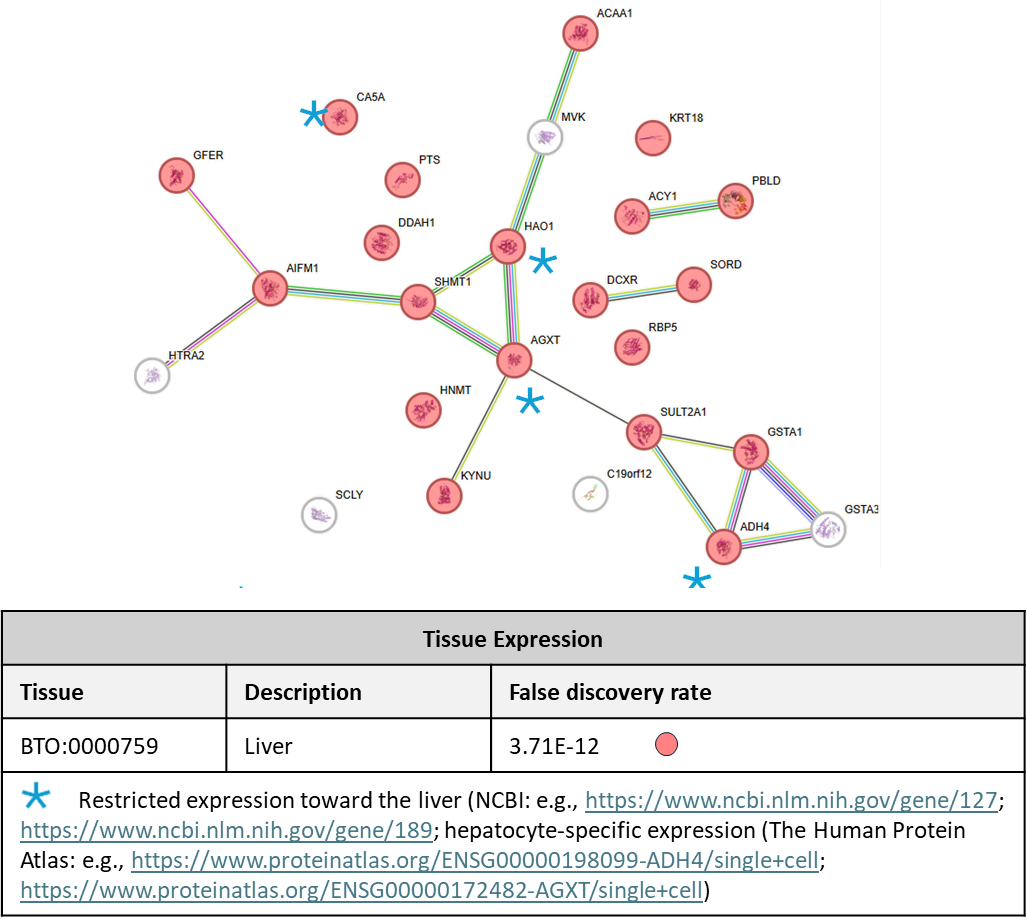


The following proteins are known to be expressed in the liver according to the String database: ACAA1, ACY1, ADH4, AGXT, AIFM1, CA5A, DCXR, DDAH1, GFER, GSTA1, HAO1, HNMT, KRT18, KYNU, PBLD, PTS, RBP5, SHMT1, SORD, SULT2A1. The edges (connecting lines) indicate that the linked proteins are part of the same physical complex; however, this may not signify that they are directly binding to each other. Known interactions are represented by purple (experimentally determined) and cyan (from curated databases) edges; predicted interactions are represented by green (gene neighborhood), red (gene fusions), and dark blue (gene co-occurrence) edges; others are represented by olive green (textmining), black (co-expression), and light blue (protein homology) edges.

ACAA1, acetyl-coenzyme A acyltransferase 1; ACY1, aminoacylase 1; ADH4, alcohol dehydrogenase 4 class II; AGXT, alanine-glyoxylate aminotransferase; AIFM1, apoptosis-inducing factor mitochondria associated 1; C19orf12, chromosome 19 open reading frame 12; CA5A, carbonic anhydrase 5A; DCXR, dicarbonyl and L-xylulose reductase; DDAH1, dimethylarginine dimethylaminohydrolase 1; GFER, growth factor, augmenter of liver regeneration; GSTA1, glutathione S-transferase alpha 1; GSTA3, glutathione S-transferase alpha 3; HAO1, hydroxyacid oxidase 1; HNMT, histamine *N*-methyltransferase; HTRA2, HtrA serine peptidase 2; KRT18, keratin type I cytoskeletal 18; KYNU, kynureninase; MVK, mevalonate kinase; PBLD, phenazine biosynthesis-like domain-containing; PTS, 6-pyruvoyl-tetrahydropterin synthase; RBP5, retinol-binding protein 5; SCLY, selenocysteine lyase; SHMT1, serine hydroxymethyltransferase; SORD, sorbitol dehydrogenase; SULT2A1, sulfotransferase family 2A member 1.

### **Fig. S8. Change from baseline in mean ADH4 (a) and CA5A (b) protein expression in responders versus non-responders for Arms 1 and 2**


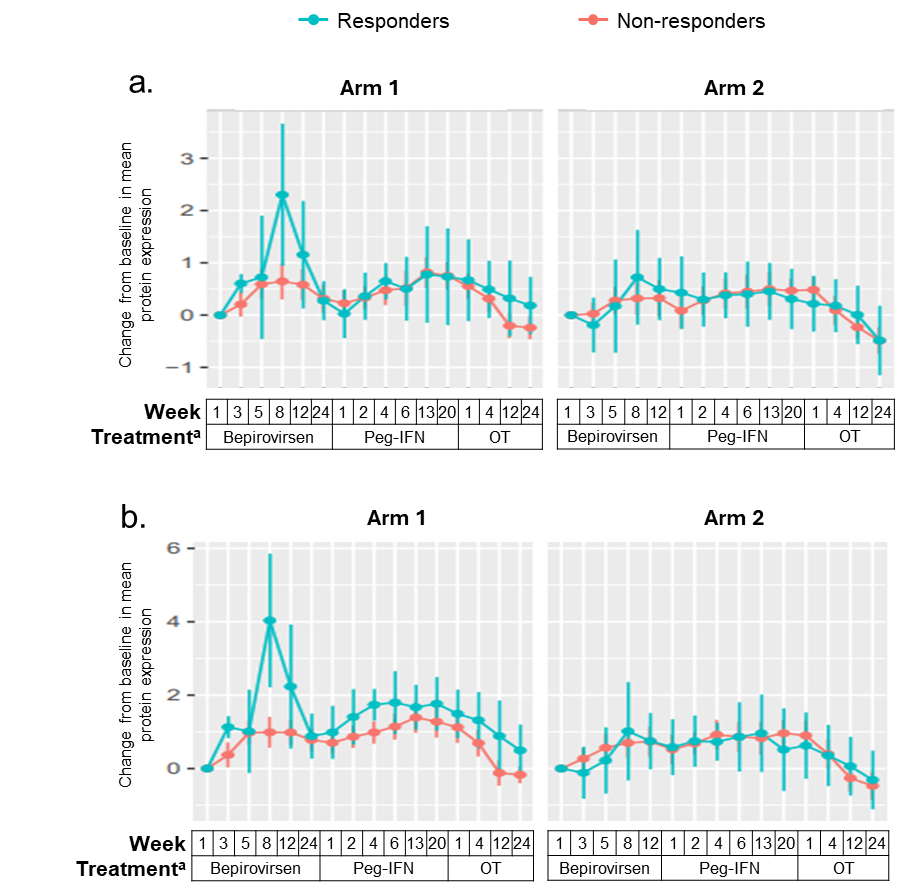


Panels show participants from Arm 1 (bepirovirsen 300 mg weekly + LD for 24 weeks followed by up to 24 weeks of Peg-IFN) and Arm 2 (bepirovirsen 300 mg weekly + LD for 12 weeks followed by up to 24 weeks of Peg-IFN) in B-Together. Data represent mean (95% CI) fold change from baseline in protein expression.

^a^Samples taken pre-dose; Week 1 of the bepirovirsen treatment window is before the first bepirovirsen dose (baseline), Week 1 of the Peg-IFN window is before the first Peg-IFN dose and Week 1 of the off-treatment window is after the last Peg-IFN dose.

ADH4, alcohol dehydrogenase 4 class II; CA5A, carbonic anhydrase 5A; LD, loading dose; OT, off-treatment; Peg-IFN, pegylated interferon-α-2a; wk, week.

### **Fig. S9. Change from baseline in mean AIFM1 (a) and KRT18 (b) protein expression in responders versus non-responders (pooled data across treatment arms)**


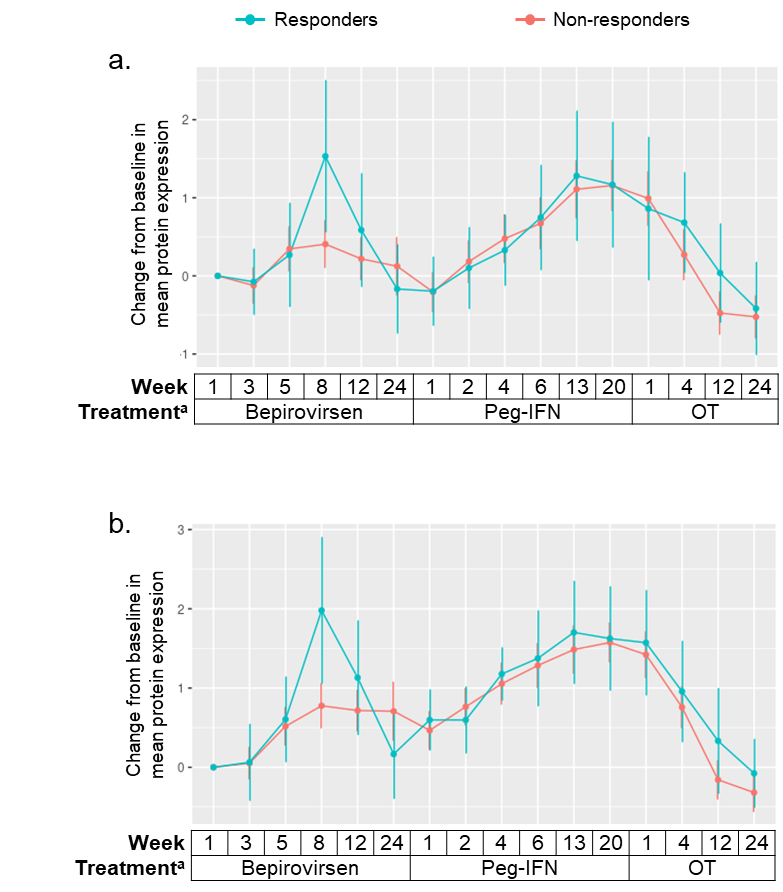


^a^Samples taken pre-dose; Week 1 of the bepirovirsen treatment window is before the first bepirovirsen dose (baseline), Week 1 of the Peg-IFN window is before the first Peg-IFN dose, and Week 1 of the off-treatment window is after the last Peg-IFN dose.

AIFM1, apoptosis-inducing factor mitochondria associated 1; KRT18, keratin type I cytoskeletal 18; OT, off-treatment; Peg-IFN, pegylated interferon-α-2a.

### **Fig. S10. Mean AIFM1 (a), KRT18 (b), ADH4 (c), and CA5A (d) protein expression in responders versus null responders for Arms 1 and 2 at Week 8**


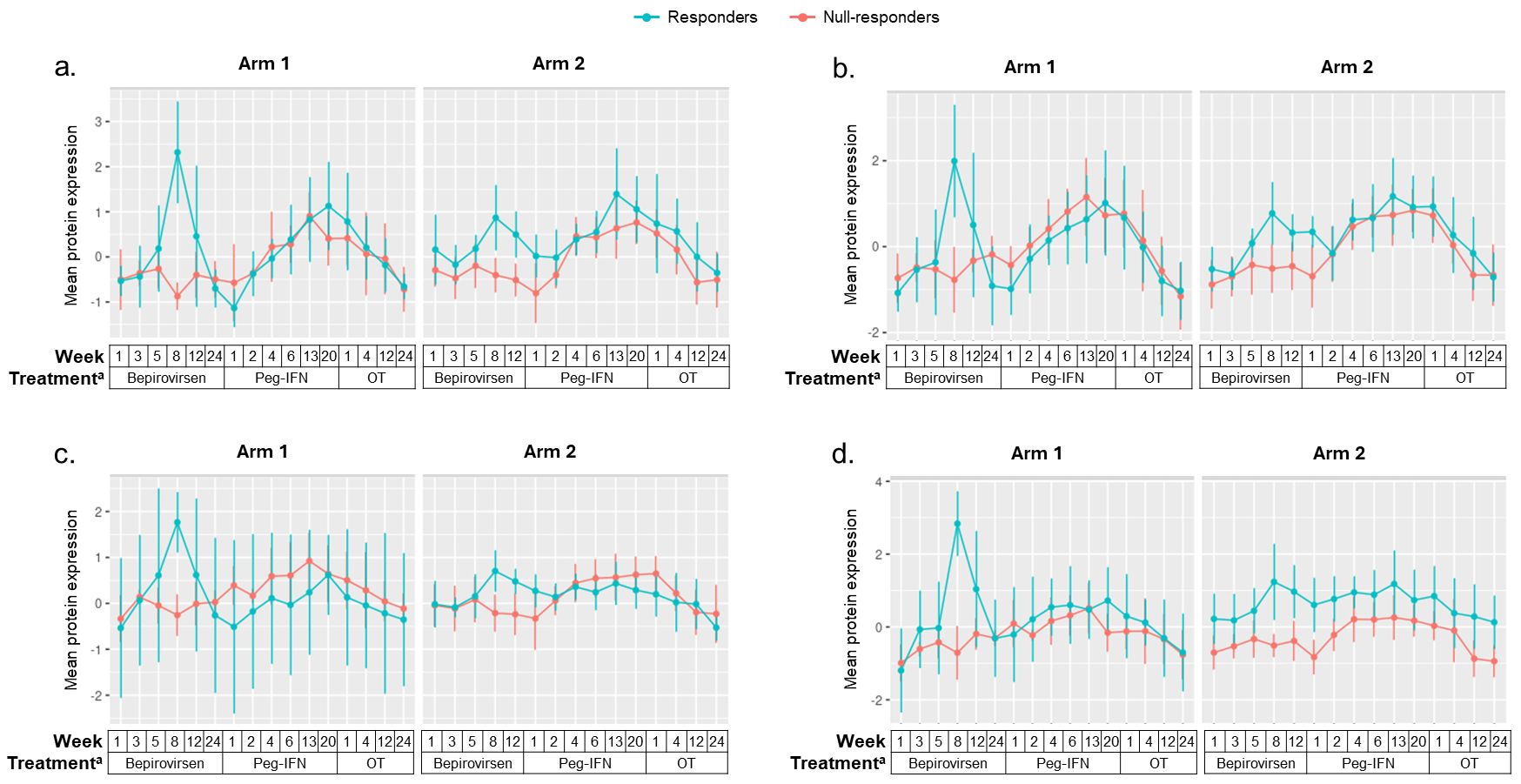


Panels show participants from Arm 1 (bepirovirsen 300 mg weekly + LD for 24 weeks followed by up to 24 weeks of Peg-IFN) and Arm 2 (bepirovirsen 300 mg weekly + LD for 12 weeks followed by up to 24 weeks of Peg-IFN) in B-Together. Data represent mean (95% CI) protein expression.

^a^Samples taken pre-dose; Week 1 of the bepirovirsen treatment window is before the first bepirovirsen dose (baseline), Week 1 of the Peg-IFN window is before the first Peg-IFN dose, and Week 1 of the off-treatment window is after the last Peg-IFN dose.

ADH4, alcohol dehydrogenase 4 class II; AIFM1, apoptosis-inducing factor mitochondria associated 1; CA5A, carbonic anhydrase 5A; CI, confidence interval; KRT18, keratin type I cytoskeletal 18; OT, off-treatment; Peg-IFN, pegylated interferon-α-2a; wk, week.

### **Fig. S11. Change from baseline in mean CD34 (a) THY-1 (b) and PECAM1 (c) protein expression in responders versus non-responders for Arms 1 and 2**


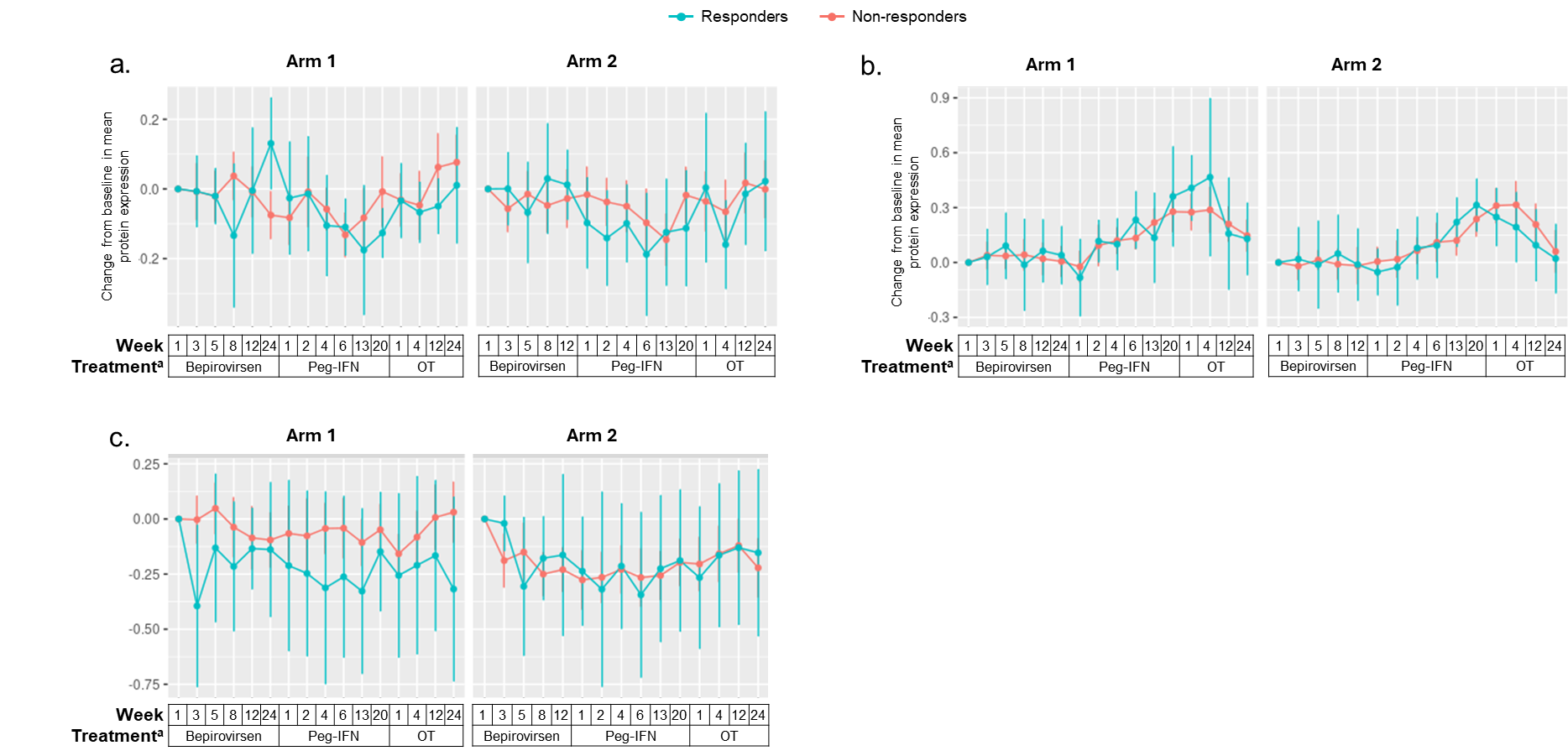


Panels show participants from Arm 1 (bepirovirsen 300 mg weekly + LD for 24 weeks followed by up to 24 weeks of Peg-IFN) and Arm 2 (bepirovirsen 300 mg weekly + LD for 12 weeks followed by up to 24 weeks of Peg-IFN) in B-Together. Data represent mean (95% CI) protein expression.

^a^Samples taken pre-dose; Week 1 of the bepirovirsen treatment window is before the first bepirovirsen dose (baseline), Week 1 of the Peg-IFN window is before the first Peg-IFN dose, and Week 1 of the off-treatment window is after the last Peg-IFN dose.

CD, cluster of differentiation; CI, confidence interval; LD, loading dose; OT, off-treatment; PECAM, platelet endothelial cell adhesion molecule; Peg-IFN, pegylated interferon-α-2a; wk, week.

## Supplementary Tables

### **Table S1. P-values for the analysis of change from baseline in protein expression in Arm 1 non-responders (a), Arm 1 responders (b), Arm 2 non-responders (c) and Arm 2 responders (d)**

**a.**

|  | **Arm 1 non-responder (N=32)** | | | | | | | | | | | | | | | | |
| --- | --- | --- | --- | --- | --- | --- | --- | --- | --- | --- | --- | --- | --- | --- | --- | --- | --- |
| **Treatment^a^** | **Bepirovirsen** | | | | | | **Peg-IFN** | | | | | | **Off-treatment** | | | |  |
| **Week** | **Baseline** | **Week 3** | **Week 5** | **Week 8** | **Week 12** | **Week 24** | **Week 1** | **Week 2** | **Week 4** | **Week 6** | **Week 13** | **Week 20** | **Week 1** | **Week 4** | **Week 12** | **Week 24** |  |
| **AKR1C4** | 1.00E+00 | 1.16E-03 | 2.50E-08 | 6.16E-06 | 9.45E-04 | 7.01E-03 | 2.54E-02 | 4.64E-03 | 1.39E-03 | 2.91E-02 | 9.36E-03 | 4.43E-05 | 5.16E-04 | 7.97E-03 | 8.40E-01 | 8.68E-01 |  |
| **TNF** | 1.00E+00 | 8.63E-07 | 5.06E-07 | 7.01E-08 | 1.93E-07 | 1.19E-06 | 5.75E-07 | 1.04E-10 | 1.97E-10 | 8.99E-10 | 6.82E-12 | 1.22E-11 | 1.43E-10 | 1.54E-05 | 2.15E-01 | 3.73E-01 |  |
| **CXCL13** | 1.00E+00 | 3.53E-09 | 2.79E-07 | 5.10E-04 | 8.77E-05 | 1.27E-07 | 1.62E-05 | 5.59E-10 | 3.55E-08 | 1.24E-07 | 2.11E-12 | 2.33E-15 | 7.46E-13 | 1.40E-05 | 1.33E-02 | 9.88E-02 |  |
| **IL10** | 1.00E+00 | 9.31E-07 | 3.82E-07 | 1.02E-05 | 3.14E-05 | 5.55E-09 | 1.18E-07 | 3.09E-13 | 4.81E-10 | 1.63E-15 | 3.57E-23 | 1.44E-20 | 1.29E-16 | 1.66E-07 | 2.52E-01 | 8.48E-01 |  |
| **IL12A_IL12B** | 1.00E+00 | 7.05E-08 | 1.53E-07 | 3.14E-05 | 1.32E-05 | 8.79E-09 | 4.41E-09 | 1.71E-17 | 2.99E-20 | 1.13E-22 | 1.83E-30 | 4.57E-24 | 2.92E-28 | 4.55E-16 | 3.16E-04 | 2.01E-02 |  |
| **TNF** | 1.00E+00 | 8.63E-07 | 5.06E-07 | 7.01E-08 | 1.93E-07 | 1.19E-06 | 5.75E-07 | 1.04E-10 | 1.97E-10 | 8.99E-10 | 6.82E-12 | 1.22E-11 | 1.43E-10 | 1.54E-05 | 2.15E-01 | 3.73E-01 |  |
| **IL5** | 1.00E+00 | 1.63E-05 | 6.53E-04 | 1.77E-03 | 7.32E-03 | 2.32E-03 | 5.25E-02 | 1.75E-03 | 3.76E-01 | 5.41E-01 | 2.39E-01 | 6.24E-01 | 1.71E-01 | 1.96E-01 | 5.97E-02 | 5.39E-01 |  |
| **GH2** | 1.00E+00 | 5.98E-11 | 1.26E-09 | 4.83E-08 | 2.80E-08 | 1.70E-05 | 3.48E-04 | 1.00E-05 | 2.71E-03 | 6.27E-02 | 4.67E-01 | 1.23E-01 | 5.36E-02 | 4.35E-02 | 1.36E-01 | 3.87E-01 |  |
| **ATF2** | 1.00E+00 | 6.69E-05 | 1.22E-07 | 4.44E-08 | 3.79E-08 | 7.69E-04 | 3.36E-04 | 7.79E-06 | 3.85E-04 | 8.87E-03 | 1.42E-02 | 2.02E-02 | 3.30E-01 | 6.20E-01 | 4.65E-01 | 6.72E-01 |  |
| **LHB** | 1.00E+00 | 6.45E-11 | 2.40E-15 | 2.07E-11 | 2.17E-09 | 9.58E-06 | 3.17E-04 | 2.07E-07 | 4.65E-05 | 1.54E-02 | 1.02E-02 | 3.80E-02 | 2.84E-01 | 8.78E-01 | 3.59E-02 | 5.64E-02 |  |
| **EPHA10** | 1.00E+00 | 2.37E-06 | 2.40E-05 | 1.37E-05 | 2.54E-04 | 6.19E-02 | 1.88E-02 | 5.15E-04 | 6.74E-03 | 1.55E-01 | 2.95E-01 | 4.42E-01 | 1.45E-01 | 9.77E-01 | 4.91E-01 | 8.36E-01 |  |
| **TNF** | 1.00E+00 | 8.63E-07 | 5.06E-07 | 7.01E-08 | 1.93E-07 | 1.19E-06 | 5.75E-07 | 1.04E-10 | 1.97E-10 | 8.99E-10 | 6.82E-12 | 1.22E-11 | 1.43E-10 | 1.54E-05 | 2.15E-01 | 3.73E-01 |  |
| **TNF** | 1.00E+00 | 8.63E-07 | 5.06E-07 | 7.01E-08 | 1.93E-07 | 1.19E-06 | 5.75E-07 | 1.04E-10 | 1.97E-10 | 8.99E-10 | 6.82E-12 | 1.22E-11 | 1.43E-10 | 1.54E-05 | 2.15E-01 | 3.73E-01 |  |
| **NOS3** | 1.00E+00 | 1.86E-08 | 5.25E-07 | 3.54E-05 | 1.67E-04 | 2.69E-07 | 2.16E-06 | 3.30E-08 | 2.40E-07 | 2.73E-08 | 1.25E-13 | 2.48E-13 | 2.52E-11 | 6.50E-05 | 2.49E-03 | 2.62E-02 |  |
| **TNFRSF9** | 1.00E+00 | 1.65E-14 | 5.87E-13 | 1.35E-11 | 1.78E-12 | 2.29E-19 | 7.02E-18 | 1.81E-11 | 2.51E-07 | 6.89E-08 | 4.84E-13 | 3.17E-12 | 7.22E-10 | 6.76E-10 | 1.35E-04 | 2.50E-02 |  |
| **LYPD1** | 1.00E+00 | 5.99E-08 | 2.36E-10 | 2.23E-07 | 7.65E-07 | 5.74E-02 | 1.16E-01 | 7.15E-03 | 3.09E-01 | 4.28E-01 | 3.36E-01 | 8.72E-01 | 3.34E-01 | 4.80E-01 | 5.85E-01 | 3.30E-01 |  |
| **HSP90B1** | 1.00E+00 | 1.27E-03 | 1.17E-02 | 4.84E-04 | 1.34E-02 | 6.58E-02 | 4.66E-03 | 3.98E-02 | 5.95E-01 | 8.80E-01 | 3.55E-01 | 6.57E-01 | 6.82E-01 | 5.59E-01 | 4.78E-01 | 8.48E-01 |  |
| **GHRHR** | 1.00E+00 | 3.77E-04 | 1.22E-03 | 1.80E-05 | 4.70E-04 | 1.97E-04 | 7.96E-05 | 3.31E-03 | 2.84E-03 | 1.36E-02 | 4.00E-02 | 9.43E-03 | 2.09E-01 | 9.02E-01 | 1.94E-02 | 4.24E-01 |  |
| **COPE** | 1.00E+00 | 1.69E-04 | 1.37E-02 | 4.11E-02 | 5.35E-02 | 1.23E-01 | 4.65E-01 | 4.42E-02 | 1.43E-01 | 4.20E-02 | 2.29E-01 | 3.80E-01 | 4.95E-01 | 5.35E-01 | 8.53E-01 | 7.97E-01 |  |
| **DUOX2** | 1.00E+00 | 1.77E-06 | 6.80E-06 | 4.49E-06 | 2.65E-05 | 6.89E-03 | 1.85E-02 | 5.01E-02 | 5.87E-02 | 3.04E-01 | 6.66E-01 | 1.84E-01 | 7.74E-01 | 4.52E-01 | 2.74E-01 | 9.85E-01 |  |
| **CRX** | 1.00E+00 | 3.31E-05 | 1.10E-03 | 2.33E-02 | 1.27E-02 | 6.65E-02 | 5.20E-01 | 3.52E-02 | 7.15E-01 | 7.46E-01 | 6.71E-01 | 3.73E-01 | 9.29E-01 | 7.89E-01 | 6.59E-01 | 7.90E-01 |  |
| **NOS1** | 1.00E+00 | 1.82E-01 | 2.84E-01 | 4.81E-02 | 6.21E-01 | 7.71E-01 | 8.14E-01 | 9.54E-01 | 2.50E-01 | 4.78E-01 | 2.61E-01 | 1.34E-01 | 1.05E-02 | 6.68E-01 | 3.48E-01 | 2.58E-01 |  |
| **SMARCA2** | 1.00E+00 | 2.51E-04 | 9.82E-07 | 2.40E-03 | 7.57E-03 | 2.63E-02 | 8.72E-03 | 1.29E-03 | 1.10E-02 | 2.66E-02 | 3.70E-01 | 6.65E-02 | 5.49E-01 | 2.99E-01 | 3.58E-02 | 3.81E-01 |  |
| **GFOD2** | 1.00E+00 | 2.03E-05 | 1.19E-05 | 5.42E-05 | 9.30E-04 | 5.11E-03 | 7.52E-03 | 1.83E-03 | 5.89E-02 | 2.03E-01 | 3.94E-01 | 4.52E-01 | 8.86E-01 | 5.12E-01 | 4.47E-01 | 3.68E-01 |  |
| **FCER2** | 1.00E+00 | 3.15E-16 | 3.43E-20 | 2.50E-19 | 1.99E-16 | 8.41E-17 | 7.20E-14 | 8.95E-10 | 5.86E-03 | 2.63E-01 | 8.99E-01 | 1.77E-01 | 6.29E-01 | 3.76E-03 | 1.53E-04 | 3.33E-03 |  |
| **IL11** | 1.00E+00 | 1.05E-06 | 1.08E-04 | 5.26E-06 | 1.63E-03 | 1.76E-04 | 4.31E-03 | 3.98E-03 | 3.57E-03 | 1.11E-01 | 7.70E-02 | 6.75E-01 | 2.59E-01 | 1.28E-01 | 4.24E-02 | 5.64E-01 |  |
| **CTF1** | 1.00E+00 | 2.57E-05 | 2.00E-07 | 3.31E-04 | 1.75E-01 | 2.34E-01 | 5.19E-01 | 1.48E-01 | 5.59E-01 | 7.93E-01 | 9.01E-01 | 9.15E-01 | 5.51E-01 | 7.43E-01 | 2.84E-01 | 7.44E-01 |  |
| **CKMT1A_CKMT1B** | 1.00E+00 | 1.73E-04 | 1.33E-03 | 4.49E-05 | 2.58E-06 | 7.59E-06 | 5.01E-01 | 1.61E-01 | 7.84E-02 | 7.71E-02 | 6.86E-03 | 2.36E-03 | 5.24E-06 | 2.64E-01 | 5.19E-01 | 8.53E-01 |  |
| **ANXA3** | 1.00E+00 | 7.34E-01 | 2.02E-02 | 6.36E-01 | 2.46E-01 | 3.97E-02 | 5.41E-02 | 5.51E-03 | 6.47E-04 | 9.54E-04 | 7.93E-04 | 2.05E-03 | 3.30E-05 | 2.56E-02 | 9.63E-01 | 8.75E-01 |  |

**b.**

|  | **Arm 1 responder (N=5)** | | | | | | | | | | | | | | | |
| --- | --- | --- | --- | --- | --- | --- | --- | --- | --- | --- | --- | --- | --- | --- | --- | --- |
| **Treatment^a^** | **Bepirovirsen** | | | | | | **Peg-IFN** | | | | | | **Off-treatment** | | | |
| **Week** | **Baseline** | **Week 3** | **Week 5** | **Week 8** | **Week 12** | **Week 24** | **Week 1** | **Week 2** | **Week 4** | **Week 6** | **Week 13** | **Week 20** | **Week 1** | **Week 4** | **Week 12** | **Week 24** |
| **AKR1C4** | 1.00E+00 | 1.34E-03 | 1.36E-02 | 3.15E-10 | 1.97E-05 | 2.92E-03 | 7.74E-03 | 3.19E-03 | 3.69E-03 | 6.70E-04 | 5.37E-05 | 2.92E-02 | 8.35E-04 | 5.77E-04 | 5.54E-04 | 3.48E-03 |
| **TNF** | 1.00E+00 | 1.05E-01 | 2.25E-01 | 7.73E-03 | 1.32E-02 | 1.88E-03 | 4.36E-03 | 1.93E-05 | 5.73E-04 | 1.22E-04 | 2.14E-04 | 4.53E-03 | 6.86E-05 | 1.24E-02 | 4.12E-01 | 4.85E-01 |
| **CXCL13** | 1.00E+00 | 3.72E-04 | 6.11E-02 | 2.73E-03 | 3.27E-02 | 2.13E-04 | 8.66E-04 | 2.23E-04 | 8.26E-04 | 2.90E-06 | 7.96E-09 | 1.79E-06 | 7.48E-08 | 1.33E-04 | 1.42E-02 | 2.67E-02 |
| **IL10** | 1.00E+00 | 2.48E-02 | 3.36E-01 | 5.16E-04 | 1.16E-01 | 2.61E-01 | 4.54E-01 | 1.13E-03 | 4.43E-04 | 2.26E-03 | 3.61E-04 | 2.02E-03 | 2.01E-05 | 1.56E-02 | 4.53E-01 | 3.95E-01 |
| **IL12A_IL12B** | 1.00E+00 | 1.15E-03 | 3.36E-02 | 1.20E-01 | 7.43E-02 | 9.47E-03 | 2.36E-03 | 3.53E-07 | 1.01E-07 | 3.63E-10 | 4.81E-09 | 4.76E-09 | 5.61E-10 | 1.43E-06 | 4.32E-03 | 3.88E-02 |
| **TNF** | 1.00E+00 | 1.05E-01 | 2.25E-01 | 7.73E-03 | 1.32E-02 | 1.88E-03 | 4.36E-03 | 1.93E-05 | 5.73E-04 | 1.22E-04 | 2.14E-04 | 4.53E-03 | 6.86E-05 | 1.24E-02 | 4.12E-01 | 4.85E-01 |
| **IL5** | 1.00E+00 | 1.62E-01 | 3.18E-01 | 5.10E-02 | 1.59E-01 | 3.89E-01 | 1.76E-01 | 1.71E-01 | 3.49E-01 | 6.89E-01 | 7.36E-01 | 6.44E-01 | 2.20E-01 | 3.06E-01 | 5.78E-01 | 5.45E-01 |
| **GH2** | 1.00E+00 | 4.86E-02 | 1.96E-01 | 7.66E-02 | 1.80E-01 | 1.81E-01 | 4.38E-01 | 1.92E-01 | 6.52E-01 | 8.44E-01 | 7.10E-01 | 8.60E-01 | 3.02E-01 | 2.91E-01 | 3.56E-01 | 1.30E-01 |
| **ATF2** | 1.00E+00 | 4.17E-02 | 6.70E-01 | 3.28E-01 | 1.51E-01 | 6.95E-02 | 1.41E-01 | 7.50E-03 | 5.85E-02 | 1.17E-01 | 5.92E-01 | 2.82E-01 | 1.71E-01 | 2.90E-01 | 1.83E-01 | 2.12E-01 |
| **LHB** | 1.00E+00 | 6.93E-03 | 7.04E-02 | 6.64E-03 | 4.66E-02 | 4.32E-01 | 3.33E-01 | 1.14E-01 | 5.20E-01 | 1.77E-01 | 3.45E-01 | 8.05E-01 | 2.29E-01 | 8.37E-02 | 6.71E-01 | 9.14E-01 |
| **EPHA10** | 1.00E+00 | 8.64E-02 | 3.59E-02 | 1.85E-02 | 3.63E-03 | 4.93E-02 | 5.83E-01 | 1.63E-02 | 1.11E-02 | 5.78E-02 | 4.58E-01 | 2.10E-01 | 2.10E-01 | 2.65E-01 | 4.49E-03 | 5.37E-02 |
| **TNF** | 1.00E+00 | 1.05E-01 | 2.25E-01 | 7.73E-03 | 1.32E-02 | 1.88E-03 | 4.36E-03 | 1.93E-05 | 5.73E-04 | 1.22E-04 | 2.14E-04 | 4.53E-03 | 6.86E-05 | 1.24E-02 | 4.12E-01 | 4.85E-01 |
| **TNF** | 1.00E+00 | 1.05E-01 | 2.25E-01 | 7.73E-03 | 1.32E-02 | 1.88E-03 | 4.36E-03 | 1.93E-05 | 5.73E-04 | 1.22E-04 | 2.14E-04 | 4.53E-03 | 6.86E-05 | 1.24E-02 | 4.12E-01 | 4.85E-01 |
| **NOS3** | 1.00E+00 | 1.55E-02 | 8.92E-01 | 1.18E-01 | 2.01E-01 | 4.65E-03 | 1.57E-01 | 3.62E-02 | 6.44E-02 | 1.64E-04 | 1.65E-06 | 3.52E-04 | 1.05E-03 | 6.39E-03 | 9.14E-03 | 1.36E-01 |
| **TNFRSF9** | 1.00E+00 | 1.63E-04 | 1.78E-02 | 5.38E-04 | 2.06E-03 | 9.38E-03 | 1.83E-02 | 1.47E-02 | 2.19E-02 | 5.24E-03 | 2.75E-03 | 7.64E-04 | 3.08E-03 | 4.97E-03 | 9.31E-02 | 6.57E-02 |
| **LYPD1** | 1.00E+00 | 7.89E-04 | 5.34E-01 | 5.34E-03 | 1.63E-01 | 1.09E-01 | 2.65E-01 | 3.32E-01 | 3.39E-01 | 2.32E-01 | 7.44E-01 | 1.39E-01 | 5.29E-01 | 1.56E-01 | 2.89E-01 | 5.21E-01 |
| **HSP90B1** | 1.00E+00 | 1.23E-01 | 3.71E-01 | 1.72E-01 | 3.24E-01 | 2.94E-01 | 2.00E-01 | 3.54E-01 | 8.22E-01 | 2.74E-01 | 9.90E-01 | 7.88E-01 | 7.44E-01 | 9.31E-01 | 5.78E-01 | 9.45E-01 |
| **GHRHR** | 1.00E+00 | 3.19E-01 | 4.30E-01 | 2.57E-01 | 5.44E-01 | 9.82E-01 | 6.68E-01 | 9.22E-01 | 8.97E-01 | 8.14E-01 | 7.58E-01 | 8.80E-01 | 9.91E-01 | 5.55E-01 | 7.41E-01 | 1.77E-01 |
| **COPE** | 1.00E+00 | 2.65E-01 | 6.20E-01 | 3.64E-01 | 1.81E-01 | 6.04E-01 | 7.38E-01 | 3.69E-01 | 6.85E-01 | 3.42E-01 | 5.48E-01 | 5.87E-01 | 5.61E-02 | 1.11E-01 | 8.08E-02 | 1.87E-01 |
| **DUOX2** | 1.00E+00 | 6.40E-01 | 8.50E-01 | 7.31E-01 | 2.38E-02 | 6.26E-01 | 3.70E-01 | 3.55E-01 | 9.91E-01 | 5.87E-01 | 1.68E-01 | 8.82E-01 | 5.59E-01 | 3.78E-01 | 3.76E-01 | 3.04E-01 |
| **CRX** | 1.00E+00 | 2.71E-01 | 9.26E-01 | 1.84E-01 | 8.06E-01 | 5.57E-01 | 8.58E-01 | 6.80E-01 | 9.92E-01 | 9.04E-01 | 2.05E-01 | 4.42E-01 | 4.98E-01 | 8.05E-01 | 7.24E-01 | 3.13E-01 |
| **NOS1** | 1.00E+00 | 3.40E-01 | 5.73E-01 | 2.02E-01 | 4.20E-01 | 4.89E-01 | 1.19E-01 | 6.74E-01 | 3.20E-01 | 7.43E-01 | 9.38E-01 | 3.24E-01 | 4.34E-01 | 3.96E-01 | 3.77E-01 | 3.75E-01 |
| **SMARCA2** | 1.00E+00 | 2.24E-01 | 1.08E-02 | 1.76E-02 | 1.60E-01 | 1.14E-01 | 5.39E-02 | 1.25E-01 | 1.08E-01 | 8.06E-01 | 8.42E-01 | 5.63E-02 | 8.16E-01 | 2.95E-01 | 5.90E-03 | 7.16E-02 |
| **GFOD2** | 1.00E+00 | 3.83E-03 | 4.65E-02 | 4.21E-03 | 8.58E-02 | 1.15E-01 | 2.13E-01 | 1.98E-01 | 4.44E-01 | 2.06E-01 | 1.66E-01 | 9.06E-01 | 3.72E-01 | 6.44E-01 | 4.91E-01 | 5.70E-01 |
| **FCER2** | 1.00E+00 | 2.88E-04 | 1.40E-03 | 5.63E-04 | 2.95E-03 | 1.05E-03 | 7.95E-03 | 4.64E-02 | 5.50E-01 | 3.60E-01 | 9.92E-01 | 7.56E-01 | 8.93E-01 | 4.51E-01 | 1.96E-01 | 5.29E-01 |
| **IL11** | 1.00E+00 | 2.78E-01 | 8.08E-01 | 3.17E-01 | 1.20E-01 | 5.93E-01 | 9.95E-01 | 6.94E-01 | 9.49E-01 | 4.66E-01 | 6.34E-01 | 9.55E-01 | 8.12E-01 | 8.12E-01 | 5.67E-01 | 8.03E-01 |
| **CTF1** | 1.00E+00 | 4.80E-01 | 3.14E-01 | 6.26E-01 | 2.81E-01 | 9.99E-01 | 7.42E-01 | 9.13E-01 | 3.66E-01 | 2.88E-01 | 2.61E-01 | 7.36E-01 | 3.04E-02 | 4.80E-01 | 7.00E-01 | 3.24E-01 |
| **CKMT1A_CKMT1B** | 1.00E+00 | 8.67E-03 | 8.44E-02 | 1.18E-01 | 8.54E-02 | 1.46E-01 | 5.08E-03 | 7.49E-01 | 9.84E-01 | 7.87E-01 | 2.97E-01 | 7.17E-01 | 7.40E-01 | 2.72E-01 | 3.38E-01 | 1.49E-01 |
| **ANXA3** | 1.00E+00 | 3.72E-01 | 9.29E-01 | 2.06E-01 | 2.68E-01 | 8.86E-02 | 2.59E-02 | 1.97E-02 | 1.68E-02 | 6.16E-03 | 1.53E-03 | 2.58E-02 | 6.89E-03 | 7.58E-02 | 6.00E-01 | 2.85E-02 |

**c.**

|  | **Arm 2 non-responder (N=37)** | | | | | | | | | | | | | | |
| --- | --- | --- | --- | --- | --- | --- | --- | --- | --- | --- | --- | --- | --- | --- | --- |
| **Treatment^a^** | **Bepirovirsen** | | | | | **Peg-IFN** | | | | | | **Off-treatment** | | | |
| **Week** | **Baseline** | **Week 3** | **Week 5** | **Week 8** | **Week 12** | **Week 1** | **Week 2** | **Week 4** | **Week 6** | **Week 13** | **Week 20** | **Week 1** | **Week 4** | **Week 12** | **Week 24** |
| **AKR1C4** | 1.00E+00 | 1.03E-04 | 1.26E-05 | 8.00E-04 | 4.63E-05 | 2.20E-02 | 2.56E-04 | 3.77E-03 | 1.78E-02 | 4.41E-03 | 1.69E-03 | 7.94E-03 | 2.72E-02 | 5.49E-01 | 5.16E-01 |
| **TNF** | 1.00E+00 | 5.79E-15 | 1.11E-11 | 4.97E-09 | 1.80E-08 | 9.34E-06 | 2.09E-15 | 1.80E-10 | 6.68E-09 | 2.08E-10 | 1.10E-09 | 4.04E-08 | 3.04E-05 | 2.59E-02 | 2.23E-01 |
| **CXCL13** | 1.00E+00 | 1.18E-14 | 9.76E-08 | 1.07E-03 | 2.59E-04 | 3.92E-04 | 1.47E-05 | 2.57E-04 | 7.30E-05 | 1.42E-04 | 2.72E-06 | 3.36E-08 | 8.55E-04 | 1.06E-02 | 7.42E-01 |
| **IL10** | 1.00E+00 | 3.05E-07 | 4.15E-06 | 1.93E-04 | 5.54E-06 | 4.18E-04 | 1.52E-13 | 1.33E-10 | 9.86E-11 | 6.72E-15 | 5.76E-13 | 1.57E-15 | 2.78E-05 | 2.61E-01 | 8.89E-01 |
| **IL12A_IL12B** | 1.00E+00 | 2.55E-10 | 1.59E-06 | 7.05E-04 | 2.32E-03 | 2.94E-03 | 1.48E-12 | 9.37E-16 | 4.33E-18 | 9.37E-23 | 5.00E-25 | 4.52E-26 | 5.04E-16 | 1.31E-04 | 7.26E-02 |
| **TNF** | 1.00E+00 | 5.79E-15 | 1.11E-11 | 4.97E-09 | 1.80E-08 | 9.34E-06 | 2.09E-15 | 1.80E-10 | 6.68E-09 | 2.08E-10 | 1.10E-09 | 4.04E-08 | 3.04E-05 | 2.59E-02 | 2.23E-01 |
| **IL5** | 1.00E+00 | 1.41E-07 | 8.83E-07 | 1.08E-06 | 4.23E-07 | 9.57E-09 | 6.41E-05 | 1.26E-04 | 2.91E-02 | 2.22E-02 | 8.77E-03 | 2.19E-02 | 1.61E-03 | 2.14E-04 | 1.75E-02 |
| **GH2** | 1.00E+00 | 4.57E-11 | 7.14E-12 | 1.67E-09 | 4.05E-08 | 8.53E-04 | 3.70E-06 | 5.10E-04 | 7.60E-03 | 4.68E-02 | 1.64E-02 | 5.40E-02 | 5.70E-02 | 2.83E-02 | 1.49E-01 |
| **ATF2** | 1.00E+00 | 4.66E-09 | 2.45E-06 | 1.78E-05 | 9.41E-07 | 1.97E-05 | 6.09E-05 | 1.90E-04 | 3.90E-03 | 2.52E-01 | 9.30E-02 | 4.96E-01 | 9.57E-01 | 8.70E-01 | 9.73E-01 |
| **LHB** | 1.00E+00 | 1.58E-13 | 3.55E-11 | 4.85E-11 | 8.41E-14 | 3.29E-09 | 7.91E-12 | 8.43E-07 | 6.59E-05 | 2.04E-03 | 7.19E-03 | 4.53E-02 | 7.42E-02 | 3.82E-02 | 1.38E-01 |
| **EPHA10** | 1.00E+00 | 1.26E-08 | 3.14E-09 | 5.21E-07 | 3.35E-09 | 1.22E-06 | 2.91E-06 | 3.91E-03 | 3.91E-04 | 8.80E-02 | 2.31E-01 | 2.12E-01 | 3.40E-01 | 1.06E-01 | 3.96E-01 |
| **TNF** | 1.00E+00 | 5.79E-15 | 1.11E-11 | 4.97E-09 | 1.80E-08 | 9.34E-06 | 2.09E-15 | 1.80E-10 | 6.68E-09 | 2.08E-10 | 1.10E-09 | 4.04E-08 | 3.04E-05 | 2.59E-02 | 2.23E-01 |
| **TNF** | 1.00E+00 | 5.79E-15 | 1.11E-11 | 4.97E-09 | 1.80E-08 | 9.34E-06 | 2.09E-15 | 1.80E-10 | 6.68E-09 | 2.08E-10 | 1.10E-09 | 4.04E-08 | 3.04E-05 | 2.59E-02 | 2.23E-01 |
| **NOS3** | 1.00E+00 | 8.37E-14 | 1.38E-07 | 9.50E-04 | 1.54E-04 | 7.41E-02 | 4.90E-06 | 6.61E-04 | 8.21E-06 | 1.25E-04 | 1.01E-07 | 1.95E-08 | 4.05E-05 | 7.94E-03 | 2.44E-01 |
| **TNFRSF9** | 1.00E+00 | 9.17E-27 | 3.21E-16 | 6.95E-14 | 1.77E-15 | 1.03E-10 | 1.76E-08 | 2.54E-05 | 1.51E-05 | 1.02E-05 | 8.15E-07 | 1.57E-07 | 2.36E-08 | 2.34E-03 | 3.48E-02 |
| **LYPD1** | 1.00E+00 | 1.10E-10 | 3.20E-08 | 1.43E-05 | 2.55E-05 | 3.66E-04 | 1.44E-03 | 5.48E-03 | 6.88E-02 | 4.53E-01 | 2.74E-01 | 2.97E-01 | 1.35E-01 | 1.39E-01 | 5.72E-01 |
| **HSP90B1** | 1.00E+00 | 1.53E-07 | 6.09E-04 | 4.26E-04 | 3.04E-05 | 6.72E-02 | 3.95E-03 | 2.30E-03 | 1.98E-02 | 1.14E-01 | 1.35E-02 | 1.87E-01 | 4.44E-01 | 4.96E-01 | 9.72E-01 |
| **GHRHR** | 1.00E+00 | 3.78E-02 | 1.99E-02 | 2.22E-01 | 1.82E-01 | 7.15E-02 | 5.07E-01 | 5.35E-01 | 7.98E-01 | 3.85E-01 | 6.34E-01 | 4.36E-01 | 7.91E-01 | 7.46E-01 | 8.95E-01 |
| **COPE** | 1.00E+00 | 2.79E-02 | 2.03E-02 | 1.06E-01 | 6.99E-02 | 3.71E-02 | 6.58E-02 | 1.45E-02 | 3.02E-01 | 4.50E-01 | 3.02E-02 | 5.72E-02 | 2.30E-01 | 7.82E-01 | 7.44E-01 |
| **DUOX2** | 1.00E+00 | 5.50E-11 | 3.49E-07 | 5.97E-07 | 6.20E-08 | 8.46E-06 | 1.17E-04 | 6.10E-04 | 3.43E-02 | 3.91E-01 | 9.91E-01 | 6.74E-01 | 2.00E-01 | 5.16E-02 | 3.34E-01 |
| **CRX** | 1.00E+00 | 3.43E-08 | 3.91E-11 | 2.07E-09 | 5.41E-06 | 4.14E-03 | 2.78E-03 | 4.04E-05 | 1.57E-01 | 5.31E-01 | 1.81E-03 | 5.83E-01 | 9.90E-02 | 4.37E-01 | 2.48E-01 |
| **NOS1** | 1.00E+00 | 2.60E-01 | 4.81E-01 | 8.02E-01 | 1.24E-01 | 1.12E-01 | 7.88E-01 | 7.56E-01 | 3.69E-01 | 5.76E-01 | 3.05E-01 | 1.60E-01 | 5.23E-01 | 2.47E-01 | 5.24E-01 |
| **SMARCA2** | 1.00E+00 | 1.47E-01 | 2.94E-01 | 1.03E-01 | 5.30E-02 | 4.14E-02 | 3.70E-01 | 3.84E-01 | 3.46E-01 | 9.30E-01 | 2.25E-01 | 1.41E-01 | 5.29E-01 | 3.66E-01 | 8.28E-02 |
| **GFOD2** | 1.00E+00 | 5.23E-05 | 1.62E-04 | 5.37E-04 | 3.95E-03 | 2.87E-01 | 4.81E-02 | 1.60E-01 | 9.46E-01 | 7.70E-01 | 6.78E-01 | 9.00E-01 | 7.85E-01 | 4.89E-01 | 4.34E-01 |
| **FCER2** | 1.00E+00 | 7.69E-27 | 3.35E-18 | 3.10E-16 | 1.98E-17 | 8.65E-13 | 1.82E-07 | 8.12E-03 | 5.07E-01 | 6.05E-01 | 8.43E-01 | 6.03E-01 | 4.76E-02 | 8.39E-03 | 1.36E-01 |
| **IL11** | 1.00E+00 | 2.17E-02 | 4.07E-05 | 1.85E-04 | 2.04E-02 | 1.02E-01 | 3.95E-02 | 1.99E-01 | 3.77E-01 | 5.55E-01 | 1.70E-01 | 6.78E-01 | 4.39E-01 | 4.54E-01 | 2.24E-01 |
| **CTF1** | 1.00E+00 | 1.37E-01 | 1.43E-01 | 7.92E-01 | 1.28E-01 | 1.67E-01 | 5.37E-01 | 7.81E-02 | 5.66E-02 | 1.43E-02 | 8.44E-02 | 4.69E-01 | 5.23E-01 | 1.47E-01 | 2.53E-02 |
| **CKMT1A_CKMT1B** | 1.00E+00 | 1.29E-04 | 3.65E-04 | 7.37E-07 | 2.46E-05 | 5.05E-04 | 8.33E-01 | 1.79E-01 | 8.02E-01 | 4.72E-05 | 1.82E-03 | 1.52E-02 | 4.66E-01 | 4.98E-01 | 6.20E-03 |
| **ANXA3** | 1.00E+00 | 1.30E-03 | 1.22E-02 | 3.28E-03 | 1.31E-03 | 7.22E-03 | 1.85E-10 | 3.50E-08 | 3.46E-07 | 2.16E-09 | 5.15E-07 | 5.13E-10 | 9.36E-06 | 1.97E-04 | 4.95E-04 |

**d.**

|  | **Arm 2 responder (N=8)** | | | | | | | | | | | | | | |
| --- | --- | --- | --- | --- | --- | --- | --- | --- | --- | --- | --- | --- | --- | --- | --- |
| **Treatment^a^** | **Bepirovirsen** | | | | | **Peg-IFN** | | | | | | **Off-treatment** | | | |
| **Week** | **Baseline** | **Week 3** | **Week 5** | **Week 8** | **Week 12** | **Week 1** | **Week 2** | **Week 4** | **Week 6** | **Week 13** | **Week 20** | **Week 1** | **Week 4** | **Week 12** | **Week 24** |
| **AKR1C4** | 1.00E+00 | 3.97E-01 | 2.69E-02 | 3.69E-03 | 4.28E-03 | 2.49E-03 | 9.68E-03 | 7.73E-02 | 9.02E-02 | 4.44E-02 | 1.98E-01 | 5.42E-01 | 1.19E-01 | 5.53E-01 | 2.79E-01 |
| **TNF** | 1.00E+00 | 4.49E-02 | 1.80E-02 | 1.78E-03 | 2.02E-02 | 1.49E-03 | 1.33E-04 | 3.06E-04 | 4.28E-04 | 6.91E-04 | 1.25E-03 | 3.86E-03 | 9.87E-02 | 4.57E-01 | 2.90E-01 |
| **CXCL13** | 1.00E+00 | 5.79E-03 | 3.92E-03 | 5.17E-02 | 3.33E-02 | 9.28E-02 | 8.10E-03 | 3.51E-02 | 1.55E-01 | 1.89E-03 | 1.29E-03 | 1.67E-03 | 7.89E-02 | 3.65E-01 | 3.36E-01 |
| **IL10** | 1.00E+00 | 1.56E-01 | 2.04E-02 | 1.41E-01 | 2.44E-01 | 4.33E-01 | 1.19E-02 | 6.35E-02 | 1.15E-01 | 2.75E-05 | 2.19E-03 | 2.71E-04 | 1.00E-01 | 8.12E-01 | 6.98E-01 |
| **IL12A_IL12B** | 1.00E+00 | 5.43E-01 | 4.72E-02 | 1.52E-01 | 3.52E-01 | 2.16E-01 | 1.78E-01 | 1.03E-02 | 2.99E-02 | 5.62E-05 | 5.27E-05 | 9.19E-05 | 2.87E-01 | 7.47E-01 | 5.45E-01 |
| **TNF** | 1.00E+00 | 4.49E-02 | 1.80E-02 | 1.78E-03 | 2.02E-02 | 1.49E-03 | 1.33E-04 | 3.06E-04 | 4.28E-04 | 6.91E-04 | 1.25E-03 | 3.86E-03 | 9.87E-02 | 4.57E-01 | 2.90E-01 |
| **IL5** | 1.00E+00 | 4.31E-03 | 4.14E-02 | 3.58E-02 | 6.24E-01 | 3.67E-02 | 5.21E-02 | 1.26E-01 | 6.43E-01 | 8.44E-02 | 3.14E-01 | 3.03E-01 | 5.26E-01 | 4.32E-01 | 1.56E-01 |
| **GH2** | 1.00E+00 | 2.23E-01 | 1.99E-01 | 3.95E-01 | 9.26E-01 | 2.75E-01 | 5.48E-01 | 7.62E-01 | 5.62E-01 | 8.31E-01 | 4.73E-01 | 6.69E-01 | 8.77E-01 | 6.12E-01 | 6.01E-01 |
| **ATF2** | 1.00E+00 | 2.89E-04 | 2.70E-02 | 2.71E-02 | 1.26E-04 | 2.46E-02 | 5.65E-02 | 1.35E-01 | 2.16E-01 | 8.96E-01 | 5.02E-01 | 6.59E-01 | 9.29E-01 | 6.32E-01 | 8.96E-01 |
| **LHB** | 1.00E+00 | 1.67E-01 | 6.69E-02 | 1.27E-01 | 4.05E-02 | 6.31E-03 | 7.84E-02 | 1.52E-01 | 1.68E-01 | 8.97E-01 | 3.93E-01 | 7.21E-01 | 3.05E-01 | 5.10E-01 | 5.94E-01 |
| **EPHA10** | 1.00E+00 | 1.71E-01 | 2.60E-01 | 8.09E-02 | 1.27E-01 | 3.30E-02 | 7.03E-01 | 9.57E-02 | 3.74E-01 | 1.18E-01 | 4.80E-01 | 6.96E-01 | 6.88E-01 | 8.96E-01 | 8.75E-01 |
| **TNF** | 1.00E+00 | 4.49E-02 | 1.80E-02 | 1.78E-03 | 2.02E-02 | 1.49E-03 | 1.33E-04 | 3.06E-04 | 4.28E-04 | 6.91E-04 | 1.25E-03 | 3.86E-03 | 9.87E-02 | 4.57E-01 | 2.90E-01 |
| **TNF** | 1.00E+00 | 4.49E-02 | 1.80E-02 | 1.78E-03 | 2.02E-02 | 1.49E-03 | 1.33E-04 | 3.06E-04 | 4.28E-04 | 6.91E-04 | 1.25E-03 | 3.86E-03 | 9.87E-02 | 4.57E-01 | 2.90E-01 |
| **NOS3** | 1.00E+00 | 3.47E-04 | 1.79E-02 | 1.56E-01 | 2.91E-01 | 2.77E-01 | 1.70E-02 | 7.09E-01 | 3.54E-01 | 9.88E-03 | 1.63E-02 | 2.20E-02 | 3.49E-02 | 3.70E-01 | 3.73E-01 |
| **TNFRSF9** | 1.00E+00 | 3.16E-03 | 2.36E-04 | 2.08E-03 | 4.17E-02 | 1.36E-03 | 1.12E-01 | 2.46E-01 | 1.28E-01 | 6.22E-03 | 8.22E-03 | 3.97E-03 | 2.38E-01 | 6.68E-01 | 7.23E-01 |
| **LYPD1** | 1.00E+00 | 3.01E-03 | 1.12E-01 | 7.42E-03 | 1.32E-02 | 2.50E-02 | 4.24E-02 | 7.60E-03 | 1.52E-01 | 8.98E-02 | 6.51E-02 | 9.77E-02 | 4.71E-02 | 6.58E-01 | 3.04E-01 |
| **HSP90B1** | 1.00E+00 | 1.69E-03 | 4.49E-03 | 2.13E-02 | 4.01E-01 | 6.42E-03 | 7.70E-02 | 9.00E-02 | 2.30E-01 | 1.21E-01 | 4.19E-01 | 1.01E-01 | 1.89E-01 | 1.46E-01 | 1.23E-01 |
| **GHRHR** | 1.00E+00 | 1.60E-03 | 7.28E-02 | 5.78E-02 | 5.22E-01 | 1.32E-02 | 9.83E-02 | 1.76E-02 | 3.57E-02 | 5.18E-02 | 1.05E-01 | 3.89E-01 | 6.03E-02 | 4.12E-01 | 1.65E-01 |
| **COPE** | 1.00E+00 | 1.86E-01 | 3.52E-01 | 6.69E-02 | 1.79E-01 | 2.38E-01 | 6.88E-01 | 4.26E-01 | 7.76E-01 | 2.60E-01 | 2.75E-01 | 1.20E-01 | 2.06E-01 | 3.25E-01 | 3.04E-01 |
| **DUOX2** | 1.00E+00 | 5.82E-03 | 2.18E-01 | 1.27E-01 | 5.19E-02 | 8.04E-03 | 1.82E-01 | 4.17E-01 | 4.75E-01 | 7.62E-01 | 5.08E-01 | 1.32E-02 | 2.03E-02 | 2.53E-01 | 7.34E-01 |
| **CRX** | 1.00E+00 | 3.00E-01 | 6.67E-01 | 7.26E-02 | 9.46E-03 | 7.02E-02 | 3.16E-02 | 5.19E-02 | 1.88E-01 | 1.45E-01 | 5.53E-01 | 2.36E-01 | 2.58E-01 | 1.51E-01 | 5.74E-01 |
| **NOS1** | 1.00E+00 | 1.22E-03 | 4.76E-03 | 4.06E-03 | 1.02E-01 | 1.78E-01 | 2.88E-02 | 7.25E-03 | 8.36E-02 | 9.53E-02 | 3.00E-03 | 5.92E-02 | 1.96E-02 | 6.32E-02 | 1.61E-02 |
| **SMARCA2** | 1.00E+00 | 8.17E-01 | 7.97E-01 | 2.07E-01 | 9.08E-01 | 9.87E-01 | 3.23E-01 | 3.14E-01 | 3.77E-01 | 4.05E-01 | 5.88E-01 | 3.41E-01 | 8.74E-01 | 6.68E-01 | 3.17E-01 |
| **GFOD2** | 1.00E+00 | 5.12E-01 | 3.87E-01 | 5.55E-01 | 5.60E-01 | 4.82E-01 | 1.48E-01 | 1.44E-01 | 9.54E-01 | 7.81E-01 | 7.13E-01 | 3.38E-01 | 3.43E-01 | 4.59E-01 | 9.74E-02 |
| **FCER2** | 1.00E+00 | 1.65E-02 | 2.74E-03 | 5.84E-03 | 2.30E-02 | 5.83E-02 | 9.50E-01 | 7.19E-01 | 1.17E-01 | 4.24E-01 | 3.88E-01 | 5.57E-01 | 7.17E-01 | 9.78E-01 | 4.56E-01 |
| **IL11** | 1.00E+00 | 1.41E-01 | 1.34E-01 | 2.12E-01 | 1.75E-01 | 4.83E-01 | 7.17E-01 | 6.05E-01 | 8.44E-01 | 8.29E-01 | 1.18E-01 | 6.95E-01 | 1.67E-01 | 4.86E-01 | 3.67E-01 |
| **CTF1** | 1.00E+00 | 6.06E-01 | 3.81E-01 | 9.34E-01 | 3.73E-01 | 6.18E-01 | 8.31E-01 | 9.51E-02 | 7.76E-01 | 1.21E-01 | 1.29E-01 | 7.90E-03 | 1.95E-01 | 1.63E-02 | 1.89E-01 |
| **CKMT1A_CKMT1B** | 1.00E+00 | 2.40E-04 | 2.96E-04 | 2.24E-03 | 1.47E-04 | 1.51E-03 | 1.99E-01 | 8.34E-01 | 4.79E-01 | 9.28E-01 | 8.97E-01 | 1.73E-01 | 9.65E-01 | 7.69E-01 | 7.68E-01 |
| **ANXA3** | 1.00E+00 | 5.78E-02 | 1.78E-01 | 6.70E-02 | 2.29E-02 | 4.05E-02 | 2.60E-02 | 8.68E-04 | 2.47E-04 | 1.20E-04 | 3.71E-05 | 1.01E-04 | 4.00E-02 | 7.06E-03 | 6.51E-02 |

Table cells color coding: purple = p<0.0001; blue = p<0.001; orange = p<0.01; green = p<0.05; no color = non-statistically significant. Red text indicates significantly increased protein expression, and blue text indicates significantly decreased protein expression (see also corresponding heatmap in **Figure 2a**). The four TNF measurements shown in the table represent technical replicates on the Olink Explore 1536 platform.
Peg-IFN, pegylated interferon-α-2a. ^a^Samples taken pre-dose; Week 1 of the bepirovirsen treatment window is before the first bepirovirsen dose (baseline), Week 1 of the Peg-IFN window is before the first Peg-IFN dose, and Week 1 of the off-treatment window is after the last Peg-IFN dose.

### **Table S2. P-values for the analysis of change from baseline in gene expression in Arm 1 non-responders (a), Arm 1 responders (b), Arm 2 non-responders (c) and Arm 2 responders (d)**

**a.**

|  | **Arm 1 non-responder (N=32)** | | | | | | | | |
| --- | --- | --- | --- | --- | --- | --- | --- | --- | --- |
| **Treatment^a^** | **Bepirovirsen** | | | **Peg-IFN** | | | **Off-treatment** | | |
| **Week** | **Baseline** | **Week 5** | **Week 12** | **Week 1** | **Week 4** | **Week 13** | **Week 1** | **Week 12** | **Week 24** |
| **CCNB2** | 1.00E+00 | 1.55E-03 | 5.54E-01 | 9.97E-01 | 1.73E-04 | 4.64E-05 | 1.46E-11 | 4.37E-02 | 4.85E-01 |
| **TYMS** | 1.00E+00 | 4.77E-08 | 4.70E-01 | 7.67E-01 | 3.47E-07 | 3.74E-12 | 1.37E-20 | 9.70E-02 | 4.93E-01 |
| **PCLAF** | 1.00E+00 | 2.17E-05 | 8.51E-01 | 5.58E-01 | 1.05E-02 | 2.85E-04 | 1.63E-07 | 1.59E-01 | 5.17E-01 |
| **H2BC14** | 1.00E+00 | 6.99E-08 | 4.03E-01 | 4.08E-01 | 2.16E-05 | 3.25E-06 | 5.52E-13 | 2.43E-02 | 7.47E-01 |
| **MMP8** | 1.00E+00 | 8.25E-04 | 7.01E-03 | 4.26E-04 | 4.31E-08 | 1.15E-02 | 2.29E-01 | 1.22E-04 | 8.14E-01 |
| **LTF** | 1.00E+00 | 9.85E-06 | 4.16E-05 | 3.12E-05 | 1.60E-09 | 3.08E-02 | 7.79E-01 | 1.72E-06 | 5.17E-01 |
| **MS4A1** | 1.00E+00 | 1.41E-13 | 6.13E-20 | 8.67E-17 | 7.61E-02 | 3.74E-05 | 1.32E-06 | 3.45E-09 | 1.01E-09 |
| **PLEKHG1** | 1.00E+00 | 2.81E-04 | 6.52E-09 | 3.17E-10 | 2.31E-01 | 1.01E-01 | 3.59E-01 | 2.22E-09 | 4.84E-04 |
| **STEAP1B** | 1.00E+00 | 3.28E-06 | 3.26E-08 | 9.78E-06 | 1.40E-02 | 2.75E-01 | 4.83E-01 | 2.46E-05 | 4.77E-04 |
| **IL7** | 1.00E+00 | 2.42E-02 | 7.71E-05 | 1.12E-04 | 9.61E-02 | 6.65E-01 | 1.98E-01 | 8.25E-03 | 2.31E-01 |
| **FAM111B** | 1.00E+00 | 6.40E-03 | 1.98E-02 | 3.85E-02 | 4.71E-01 | 4.73E-01 | 3.32E-01 | 8.80E-03 | 6.60E-01 |
| **HNF1B** | 1.00E+00 | 6.58E-03 | 2.41E-07 | 1.54E-03 | 2.31E-01 | 7.44E-01 | 5.81E-01 | 4.07E-01 | 1.76E-01 |
| **RASSF6** | 1.00E+00 | 1.11E-01 | 2.33E-04 | 8.61E-06 | 9.39E-01 | 3.55E-01 | 3.29E-01 | 6.74E-01 | 9.25E-01 |
| **LOC101929594** | 1.00E+00 | 4.47E-01 | 5.52E-02 | 5.10E-02 | 9.52E-01 | 2.51E-01 | 8.98E-01 | 8.04E-01 | 3.24E-01 |
| **CMTM5** | 1.00E+00 | 7.98E-02 | 9.07E-03 | 6.48E-05 | 3.89E-14 | 2.04E-13 | 6.40E-14 | 4.79E-02 | 4.27E-02 |
| **GNG11** | 1.00E+00 | 3.38E-01 | 1.18E-01 | 4.35E-03 | 1.78E-16 | 3.60E-17 | 1.06E-17 | 6.41E-02 | 2.41E-01 |

**b.**

|  | **Arm 1 responder (N=5)** | | | | | | | | |
| --- | --- | --- | --- | --- | --- | --- | --- | --- | --- |
| **Treatment^a^** | **Bepirovirsen** | | | **Peg-IFN** | | | **Off-treatment** | | |
| **Week** | **Baseline** | **Week 5** | **Week 12** | **Week 1** | **Week 4** | **Week 13** | **Week 1** | **Week 12** | **Week 24** |
| **CCNB2** | 1.00E+00 | 1.12E-01 | 2.88E-01 | 8.58E-01 | 1.71E-03 | 1.95E-03 | 3.66E-03 | 1.70E-01 | 4.70E-02 |
| **TYMS** | 1.00E+00 | 1.80E-01 | 9.45E-01 | 7.29E-01 | 7.26E-06 | 2.13E-05 | 4.91E-06 | 8.53E-01 | 1.29E-02 |
| **PCLAF** | 1.00E+00 | 1.88E-01 | 9.83E-01 | 4.64E-01 | 1.37E-02 | 7.99E-02 | 5.56E-03 | 2.22E-01 | 2.18E-02 |
| **H2BC14** | 1.00E+00 | 5.93E-01 | 4.98E-01 | 3.43E-01 | 6.51E-03 | 5.87E-04 | 1.37E-03 | 9.72E-01 | 5.46E-04 |
| **MMP8** | 1.00E+00 | 3.21E-01 | 2.19E-01 | 7.49E-01 | 7.66E-03 | 1.61E-01 | 1.79E-01 | 1.12E-01 | 5.37E-01 |
| **LTF** | 1.00E+00 | 1.61E-01 | 1.88E-01 | 4.44E-01 | 1.66E-01 | 4.72E-01 | 6.62E-01 | 5.68E-02 | 9.96E-01 |
| **MS4A1** | 1.00E+00 | 2.92E-04 | 2.57E-03 | 4.20E-04 | 4.44E-01 | 6.17E-02 | 3.08E-02 | 2.83E-02 | 1.53E-01 |
| **PLEKHG1** | 1.00E+00 | 1.80E-02 | 2.45E-02 | 2.60E-02 | 9.78E-02 | 4.86E-01 | 1.38E-01 | 2.77E-02 | 8.76E-02 |
| **STEAP1B** | 1.00E+00 | 1.29E-01 | 1.70E-01 | 1.82E-01 | 7.34E-01 | 4.43E-01 | 2.59E-01 | 6.57E-01 | 9.45E-01 |
| **IL7** | 1.00E+00 | 3.71E-01 | 6.65E-02 | 1.85E-01 | 1.65E-01 | 9.30E-01 | 5.57E-01 | 4.12E-01 | 9.04E-01 |
| **FAM111B** | 1.00E+00 | 1.07E-01 | 4.62E-01 | 6.93E-01 | 1.82E-01 | 2.19E-01 | 7.81E-01 | 6.92E-01 | 5.37E-02 |
| **HNF1B** | 1.00E+00 | 2.29E-01 | 2.29E-02 | 4.42E-01 | 6.14E-01 | 8.18E-01 | 5.50E-01 | 5.29E-01 | 5.95E-01 |
| **RASSF6** | 1.00E+00 | 1.75E-01 | 5.20E-01 | 4.40E-02 | 8.17E-01 | 3.92E-01 | 9.42E-01 | 9.38E-01 | 1.92E-01 |
| **LOC101929594** | 1.00E+00 | 4.82E-01 | 2.41E-01 | 5.84E-01 | 2.05E-01 | 1.52E-01 | 2.25E-01 | 6.16E-02 | 3.62E-01 |
| **CMTM5** | 1.00E+00 | 4.00E-01 | 5.71E-02 | 6.84E-03 | 1.05E-12 | 8.85E-05 | 2.22E-06 | 4.07E-01 | 8.73E-01 |
| **GNG11** | 1.00E+00 | 9.10E-01 | 4.44E-02 | 9.16E-03 | 1.39E-07 | 2.49E-04 | 9.15E-06 | 4.61E-01 | 5.20E-01 |

**c.**

|  | **Arm 2 non-responder (N=37)** | | | | | | | | |
| --- | --- | --- | --- | --- | --- | --- | --- | --- | --- |
| **Treatment^a^** | **Bepirovirsen** | | | **Peg-IFN** | | | **Off-treatment** | | |
| **Week** | **Baseline** | **Week 5** | **Week 12** | **Week 1** | **Week 4** | **Week 13** | **Week 1** | **Week 12** | **Week 24** |
| **CCNB2** | 1.00E+00 | 1.46E-09 | 7.74E-02 | 3.74E-01 | 9.94E-11 | 3.12E-12 | 2.04E-15 | 4.50E-04 | 5.27E-01 |
| **TYMS** | 1.00E+00 | 1.15E-11 | 5.10E-02 | 1.57E-01 | 1.04E-16 | 3.37E-24 | 1.71E-24 | 5.61E-03 | 2.96E-01 |
| **PCLAF** | 1.00E+00 | 6.73E-09 | 2.31E-01 | 8.10E-01 | 2.45E-05 | 1.61E-06 | 2.06E-07 | 4.33E-01 | 1.97E-01 |
| **H2BC14** | 1.00E+00 | 5.08E-11 | 7.47E-02 | 1.18E-01 | 3.62E-10 | 7.95E-13 | 2.20E-20 | 9.41E-04 | 5.96E-01 |
| **MMP8** | 1.00E+00 | 1.22E-06 | 1.18E-02 | 2.67E-01 | 1.33E-05 | 2.38E-01 | 6.66E-01 | 9.17E-06 | 3.13E-02 |
| **LTF** | 1.00E+00 | 2.67E-06 | 6.54E-03 | 3.90E-02 | 5.07E-06 | 3.21E-01 | 4.38E-02 | 3.36E-07 | 9.28E-03 |
| **MS4A1** | 1.00E+00 | 1.76E-11 | 6.15E-15 | 2.20E-18 | 5.97E-03 | 3.65E-03 | 1.11E-04 | 6.42E-08 | 1.03E-03 |
| **PLEKHG1** | 1.00E+00 | 7.82E-03 | 4.27E-09 | 1.90E-15 | 2.04E-01 | 1.32E-01 | 7.12E-03 | 3.45E-04 | 5.47E-03 |
| **STEAP1B** | 1.00E+00 | 3.04E-07 | 5.63E-06 | 2.41E-04 | 1.91E-03 | 4.47E-02 | 3.81E-01 | 1.02E-02 | 8.97E-03 |
| **IL7** | 1.00E+00 | 5.43E-06 | 3.17E-11 | 8.61E-14 | 8.37E-04 | 8.51E-02 | 7.81E-01 | 1.92E-03 | 2.28E-02 |
| **FAM111B** | 1.00E+00 | 5.00E-13 | 1.06E-08 | 9.62E-12 | 4.01E-04 | 2.89E-04 | 9.12E-05 | 6.37E-06 | 9.69E-06 |
| **HNF1B** | 1.00E+00 | 1.17E-02 | 3.64E-04 | 1.19E-05 | 5.76E-01 | 3.87E-02 | 1.08E-01 | 4.61E-01 | 5.59E-01 |
| **RASSF6** | 1.00E+00 | 1.76E-01 | 6.09E-10 | 4.25E-07 | 2.78E-01 | 9.24E-01 | 6.19E-01 | 3.18E-01 | 9.97E-01 |
| **LOC101929594** | 1.00E+00 | 3.44E-01 | 1.54E-03 | 5.20E-05 | 1.87E-01 | 1.53E-01 | 4.58E-03 | 1.31E-03 | 1.42E-01 |
| **CMTM5** | 1.00E+00 | 3.87E-05 | 2.06E-05 | 1.88E-07 | 4.62E-23 | 1.33E-22 | 1.26E-12 | 8.25E-02 | 4.82E-01 |
| **GNG11** | 1.00E+00 | 1.93E-03 | 3.83E-05 | 6.62E-06 | 4.86E-25 | 1.16E-25 | 3.73E-20 | 1.72E-02 | 9.28E-01 |

**d.**

|  | **Arm 2 responder (N=8)** | | | | | | | | |
| --- | --- | --- | --- | --- | --- | --- | --- | --- | --- |
| **Treatment^a^** | **Bepirovirsen** | | | **Peg-IFN** | | | **Off-treatment** | | |
| **Week** | **Baseline** | **Week 5** | **Week 12** | **Week 1** | **Week 4** | **Week 13** | **Week 1** | **Week 12** | **Week 24** |
| **CCNB2** | 1.00E+00 | 9.60E-02 | 4.71E-01 | 6.51E-02 | 1.52E-01 | 9.94E-03 | 1.35E-03 | 3.35E-01 | 8.83E-01 |
| **TYMS** | 1.00E+00 | 1.40E-02 | 5.06E-01 | 9.49E-01 | 2.62E-03 | 4.74E-05 | 8.82E-07 | 2.75E-01 | 5.60E-01 |
| **PCLAF** | 1.00E+00 | 3.11E-02 | 7.26E-01 | 6.11E-01 | 1.22E-01 | 2.64E-01 | 1.15E-03 | 7.44E-01 | 4.41E-01 |
| **H2BC14** | 1.00E+00 | 3.03E-03 | 4.90E-01 | 5.04E-01 | 2.49E-02 | 1.65E-04 | 3.20E-05 | 4.11E-01 | 4.18E-01 |
| **MMP8** | 1.00E+00 | 7.22E-02 | 3.69E-02 | 5.95E-01 | 2.11E-02 | 8.40E-01 | 5.42E-01 | 2.89E-02 | 4.21E-01 |
| **LTF** | 1.00E+00 | 1.72E-03 | 1.15E-02 | 1.03E-01 | 1.07E-03 | 6.87E-01 | 2.99E-01 | 9.67E-03 | 8.34E-01 |
| **MS4A1** | 1.00E+00 | 5.80E-02 | 1.07E-06 | 1.46E-06 | 3.33E-01 | 5.21E-01 | 2.60E-02 | 3.06E-02 | 4.36E-02 |
| **PLEKHG1** | 1.00E+00 | 5.27E-01 | 1.52E-02 | 5.68E-04 | 7.53E-01 | 9.38E-01 | 9.42E-01 | 3.01E-01 | 1.88E-01 |
| **STEAP1B** | 1.00E+00 | 9.23E-01 | 4.20E-02 | 6.98E-02 | 9.78E-01 | 1.17E-01 | 2.38E-01 | 5.70E-01 | 8.68E-01 |
| **IL7** | 1.00E+00 | 3.04E-01 | 3.39E-03 | 5.08E-03 | 3.70E-01 | 6.44E-01 | 3.29E-01 | 2.31E-01 | 6.85E-01 |
| **FAM111B** | 1.00E+00 | 8.48E-03 | 1.75E-03 | 3.20E-04 | 8.06E-01 | 9.92E-01 | 5.76E-03 | 2.43E-02 | 2.32E-01 |
| **HNF1B** | 1.00E+00 | 3.94E-02 | 9.48E-03 | 1.14E-01 | 8.24E-01 | 3.95E-01 | 2.02E-01 | 8.50E-01 | 5.61E-01 |
| **RASSF6** | 1.00E+00 | 9.65E-01 | 6.54E-02 | 1.77E-01 | 9.60E-01 | 9.95E-01 | 8.28E-01 | 7.39E-01 | 8.85E-01 |
| **LOC101929594** | 1.00E+00 | 3.99E-01 | 4.76E-01 | 2.27E-01 | 8.96E-01 | 6.87E-01 | 1.40E-01 | 8.61E-01 | 1.01E-04 |
| **CMTM5** | 1.00E+00 | 9.53E-01 | 1.68E-01 | 1.36E-03 | 1.35E-06 | 1.48E-05 | 2.96E-05 | 7.64E-01 | 7.99E-01 |
| **GNG11** | 1.00E+00 | 6.65E-01 | 1.05E-02 | 5.53E-04 | 4.30E-11 | 2.00E-08 | 7.77E-12 | 1.02E-01 | 7.29E-01 |

Table cells color coding: purple = p<0.0001; blue = p<0.001; orange = p<0.01; green = p<0.05; no color = non-statistically significant. Red text indicates significantly increased protein expression, and blue text indicates significantly decreased protein expression (see also corresponding heatmap in **Figure 3a**). ^a^Samples taken pre-dose; Week 1 of the bepirovirsen treatment window is before the first bepirovirsen dose (baseline), Week 1 of the Peg-IFN window is before the first Peg-IFN dose, and Week 1 of the off-treatment window is after the last Peg-IFN dose.
Peg-IFN, pegylated interferon-α-2a.

### **Table S3. P-values for the analysis of change from baseline in protein expression in Arm 1 non-responders (a), Arm 1 responders (b), Arm 2 non-responders (c) and Arm 2 responders (d)**

**a.**

|  | **Non-responder (N=32)** | | | | | | | | | | | | | | | |
| --- | --- | --- | --- | --- | --- | --- | --- | --- | --- | --- | --- | --- | --- | --- | --- | --- |
| **Treatment^a^** | **Bepirovirsen** | | | | | | **Peg-IFN** | | | | | | **Off-treatment** | | | |
| **Week** | **Baseline** | **Week 3** | **Week 5** | **Week 8** | **Week 12** | **Week 24** | **Week 1** | **Week 2** | **Week 4** | **Week 6** | **Week 13** | **Week 20** | **Week 1** | **Week 4** | **Week 12** | **Week 24** |
| **C19orf12** | 1.00E+00 | 4.20E-02 | 1.94E-06 | 3.65E-12 | 7.75E-12 | 1.29E-06 | 2.51E-04 | 8.62E-08 | 1.58E-10 | 3.56E-12 | 1.85E-16 | 9.44E-16 | 5.52E-10 | 2.55E-04 | 5.72E-01 | 4.04E-01 |
| **CA5A** | 1.00E+00 | 1.29E-01 | 6.23E-07 | 1.62E-07 | 1.34E-07 | 1.20E-05 | 2.21E-04 | 9.81E-06 | 8.48E-07 | 4.18E-09 | 7.87E-12 | 4.45E-09 | 2.77E-07 | 1.56E-03 | 3.15E-01 | 2.12E-01 |
| **GSTA3** | 1.00E+00 | 3.39E-01 | 8.24E-05 | 1.95E-05 | 5.70E-04 | 2.05E-02 | 1.48E-01 | 2.40E-02 | 8.24E-04 | 2.52E-07 | 2.31E-09 | 3.90E-09 | 8.45E-06 | 4.44E-02 | 1.76E-02 | 1.24E-01 |
| **HAO1** | 1.00E+00 | 4.31E-01 | 1.61E-03 | 6.26E-04 | 1.01E-03 | 2.69E-02 | 1.53E-01 | 3.41E-03 | 3.60E-04 | 8.15E-07 | 2.31E-12 | 1.87E-12 | 3.94E-09 | 9.42E-04 | 3.48E-01 | 1.55E-01 |
| **KRT18** | 1.00E+00 | 7.22E-01 | 6.49E-04 | 2.72E-06 | 6.11E-06 | 6.67E-05 | 1.88E-03 | 9.95E-06 | 3.03E-08 | 8.68E-12 | 1.23E-16 | 1.24E-16 | 3.11E-13 | 1.24E-04 | 1.40E-01 | 2.59E-01 |
| **SULT2A1** | 1.00E+00 | 9.80E-01 | 5.16E-02 | 1.27E-02 | 8.45E-02 | 1.82E-02 | 5.81E-01 | 3.32E-03 | 2.77E-05 | 4.51E-08 | 2.27E-11 | 1.89E-11 | 4.15E-09 | 8.15E-04 | 2.05E-01 | 1.49E-01 |
| **TYMP** | 1.00E+00 | 7.92E-01 | 1.86E-02 | 1.04E-02 | 1.04E-01 | 2.66E-01 | 4.63E-02 | 3.87E-18 | 4.95E-30 | 1.69E-36 | 6.14E-52 | 6.22E-45 | 2.27E-44 | 5.60E-15 | 3.26E-01 | 3.79E-01 |
| **PTS** | 1.00E+00 | 3.67E-03 | 1.49E-05 | 2.35E-07 | 2.89E-05 | 2.35E-03 | 5.04E-04 | 4.35E-09 | 4.49E-12 | 3.27E-12 | 5.60E-19 | 1.40E-11 | 9.22E-09 | 4.27E-04 | 7.58E-01 | 2.81E-01 |
| **SORD** | 1.00E+00 | 5.54E-01 | 1.43E-03 | 4.95E-04 | 2.51E-02 | 3.61E-01 | 5.12E-01 | 1.79E-07 | 3.45E-08 | 2.30E-11 | 1.04E-17 | 1.40E-15 | 6.61E-13 | 6.35E-04 | 2.21E-01 | 3.60E-02 |
| **SHMT1** | 1.00E+00 | 9.17E-01 | 1.53E-03 | 1.84E-02 | 5.59E-01 | 4.60E-01 | 2.81E-01 | 1.14E-05 | 7.52E-08 | 1.78E-09 | 3.10E-15 | 8.89E-13 | 1.99E-08 | 7.93E-03 | 4.70E-02 | 2.85E-02 |
| **AIFM1** | 1.00E+00 | 6.07E-01 | 3.13E-04 | 5.87E-04 | 5.94E-02 | 3.96E-01 | 8.81E-01 | 8.14E-02 | 1.12E-02 | 8.39E-05 | 6.64E-12 | 5.59E-09 | 3.19E-06 | 3.83E-02 | 3.48E-02 | 1.03E-01 |
| **ACAA1** | 1.00E+00 | 8.39E-01 | 2.79E-02 | 5.79E-02 | 1.22E-01 | 4.72E-01 | 1.75E-01 | 9.10E-03 | 8.48E-05 | 1.76E-04 | 4.12E-10 | 4.08E-09 | 8.16E-07 | 2.36E-02 | 8.20E-01 | 6.26E-01 |
| **AGXT** | 1.00E+00 | 6.56E-01 | 1.30E-02 | 3.80E-03 | 2.91E-02 | 1.41E-01 | 5.47E-01 | 1.50E-02 | 4.22E-04 | 2.34E-06 | 2.30E-13 | 1.44E-14 | 4.75E-11 | 1.63E-03 | 6.26E-01 | 1.86E-01 |
| **GRPEL1** | 1.00E+00 | 4.41E-02 | 1.50E-03 | 5.08E-05 | 1.89E-02 | 1.47E-01 | 2.59E-01 | 2.20E-05 | 2.43E-07 | 1.05E-08 | 2.17E-20 | 4.20E-17 | 6.01E-17 | 3.72E-05 | 5.23E-01 | 4.48E-01 |
| **HNMT** | 1.00E+00 | 2.92E-01 | 1.84E-05 | 1.29E-07 | 4.26E-05 | 1.87E-04 | 3.07E-03 | 6.56E-06 | 2.25E-06 | 2.37E-09 | 3.00E-13 | 3.82E-11 | 6.71E-08 | 7.27E-04 | 5.17E-01 | 3.56E-01 |
| **PBLD** | 1.00E+00 | 4.40E-01 | 1.41E-05 | 3.70E-07 | 6.80E-03 | 8.47E-02 | 8.06E-01 | 7.07E-02 | 1.66E-02 | 4.83E-05 | 1.72E-09 | 1.36E-06 | 2.02E-02 | 7.93E-02 | 5.30E-02 | 3.25E-02 |
| **ALDH1A1** | 1.00E+00 | 4.44E-01 | 3.35E-04 | 1.69E-05 | 1.16E-02 | 1.99E-01 | 4.94E-01 | 3.21E-05 | 3.86E-05 | 1.02E-06 | 1.91E-13 | 5.06E-11 | 1.58E-08 | 3.33E-05 | 9.06E-01 | 6.80E-01 |
| **SCLY** | 1.00E+00 | 7.05E-02 | 2.64E-05 | 1.03E-07 | 1.58E-05 | 1.92E-02 | 1.13E-01 | 1.23E-04 | 2.45E-07 | 1.20E-09 | 2.87E-23 | 3.02E-20 | 1.84E-15 | 6.04E-05 | 1.70E-01 | 3.35E-02 |
| **DCXR** | 1.00E+00 | 5.31E-01 | 6.95E-04 | 1.29E-06 | 6.62E-05 | 2.28E-02 | 9.67E-02 | 1.28E-05 | 1.96E-06 | 2.82E-07 | 2.18E-15 | 8.98E-12 | 5.98E-07 | 7.02E-03 | 2.09E-01 | 5.53E-02 |
| **GFER** | 1.00E+00 | 7.95E-01 | 1.21E-03 | 3.33E-06 | 4.98E-03 | 1.32E-01 | 5.57E-01 | 5.73E-05 | 5.30E-05 | 2.10E-07 | 2.23E-17 | 1.20E-14 | 1.84E-11 | 4.33E-03 | 3.76E-01 | 1.76E-01 |
| **FABP1** | 1.00E+00 | 8.88E-01 | 1.34E-01 | 2.93E-01 | 6.54E-01 | 7.60E-01 | 7.85E-01 | 1.14E-01 | 7.26E-03 | 1.35E-05 | 2.62E-05 | 1.51E-06 | 1.42E-06 | 8.17E-03 | 2.35E-01 | 2.22E-01 |
| **FBP1** | 1.00E+00 | 5.96E-01 | 7.26E-02 | 1.17E-02 | 1.06E-01 | 3.55E-01 | 5.90E-01 | 5.09E-02 | 2.17E-03 | 1.79E-03 | 1.91E-08 | 5.58E-07 | 4.57E-06 | 8.12E-02 | 8.10E-01 | 3.69E-01 |
| **ADH4** | 1.00E+00 | 1.77E-01 | 3.85E-05 | 2.04E-06 | 2.04E-05 | 1.33E-02 | 6.49E-02 | 1.49E-02 | 1.05E-03 | 2.53E-04 | 2.33E-08 | 1.00E-06 | 2.63E-04 | 6.17E-02 | 8.27E-02 | 5.41E-02 |
| **KYNU** | 1.00E+00 | 2.52E-01 | 7.91E-04 | 4.53E-05 | 1.77E-03 | 7.32E-02 | 6.54E-01 | 2.09E-04 | 4.77E-06 | 1.83E-08 | 3.72E-18 | 2.67E-16 | 1.87E-10 | 2.10E-03 | 2.11E-01 | 3.98E-02 |
| **ACY1** | 1.00E+00 | 2.03E-01 | 3.58E-04 | 2.87E-05 | 1.23E-05 | 1.16E-02 | 2.02E-02 | 1.56E-04 | 6.19E-06 | 4.42E-07 | 2.53E-14 | 2.89E-11 | 7.31E-09 | 4.93E-03 | 1.82E-01 | 9.43E-02 |
| **HTRA2** | 1.00E+00 | 8.07E-01 | 5.78E-03 | 1.81E-05 | 1.60E-02 | 2.65E-01 | 3.48E-01 | 6.96E-04 | 6.92E-07 | 5.87E-08 | 2.33E-23 | 8.47E-19 | 3.35E-14 | 2.41E-03 | 4.28E-01 | 5.94E-01 |
| **RBP5** | 1.00E+00 | 1.50E-01 | 1.48E-04 | 3.23E-04 | 3.61E-02 | 2.46E-01 | 8.81E-01 | 3.35E-01 | 1.43E-01 | 2.31E-02 | 3.49E-06 | 2.04E-06 | 1.66E-04 | 2.96E-02 | 3.34E-02 | 2.92E-02 |
| **GSTA1** | 1.00E+00 | 4.07E-01 | 4.04E-01 | 5.99E-01 | 3.41E-01 | 2.04E-02 | 1.13E-04 | 4.54E-02 | 3.86E-01 | 4.35E-01 | 7.06E-03 | 1.23E-02 | 2.76E-01 | 4.99E-01 | 8.39E-07 | 1.22E-04 |
| **CXCL17** | 1.00E+00 | 2.86E-02 | 3.74E-01 | 4.49E-01 | 2.32E-01 | 1.11E-01 | 9.30E-01 | 4.00E-04 | 1.25E-04 | 2.63E-04 | 7.58E-04 | 3.53E-06 | 5.35E-06 | 2.45E-02 | 2.36E-01 | 3.37E-01 |
| **VWA1** | 1.00E+00 | 5.58E-01 | 8.33E-01 | 9.91E-01 | 5.16E-01 | 7.39E-01 | 7.88E-01 | 8.34E-04 | 2.10E-06 | 5.79E-08 | 6.87E-08 | 2.13E-10 | 1.52E-11 | 1.90E-04 | 1.24E-01 | 9.19E-01 |
| **GPNMB** | 1.00E+00 | 3.82E-07 | 2.23E-12 | 1.87E-15 | 1.00E-17 | 3.71E-23 | 4.76E-21 | 1.26E-14 | 1.12E-15 | 3.35E-14 | 1.94E-07 | 4.35E-06 | 5.33E-04 | 7.47E-04 | 1.89E-03 | 4.34E-02 |
| **NTF4** | 1.00E+00 | 1.31E-03 | 1.45E-03 | 5.54E-03 | 2.81E-03 | 2.71E-03 | 3.34E-04 | 1.16E-05 | 2.79E-02 | 1.08E-01 | 9.39E-01 | 1.75E-01 | 1.87E-01 | 6.08E-01 | 1.10E-02 | 4.57E-02 |
| **RAB6B** | 1.00E+00 | 2.14E-03 | 9.52E-05 | 2.59E-03 | 7.21E-02 | 2.89E-01 | 1.36E-01 | 5.36E-01 | 9.65E-01 | 8.37E-01 | 9.99E-01 | 6.05E-01 | 6.87E-01 | 4.86E-01 | 3.30E-01 | 5.04E-01 |
| **MVK** | 1.00E+00 | 9.32E-01 | 5.01E-04 | 1.57E-04 | 1.16E-02 | 7.37E-02 | 7.71E-01 | 2.29E-02 | 4.79E-02 | 7.92E-03 | 1.31E-06 | 6.22E-04 | 1.90E-02 | 1.39E-01 | 3.02E-01 | 5.64E-02 |
| **THOP1** | 1.00E+00 | 3.08E-01 | 2.98E-03 | 1.44E-04 | 3.62E-02 | 4.70E-01 | 6.11E-01 | 1.87E-03 | 1.82E-03 | 4.15E-04 | 2.95E-11 | 9.78E-09 | 9.15E-07 | 6.36E-05 | 1.42E-01 | 9.52E-01 |
| **DDAH1** | 1.00E+00 | 8.30E-02 | 1.88E-04 | 1.62E-06 | 6.79E-02 | 8.32E-02 | 8.91E-01 | 1.29E-02 | 5.20E-03 | 1.74E-05 | 5.61E-07 | 6.83E-07 | 3.00E-06 | 2.36E-02 | 8.54E-01 | 7.38E-01 |
| **RBKS** | 1.00E+00 | 6.19E-01 | 8.92E-04 | 3.77E-04 | 1.49E-01 | 3.82E-01 | 9.89E-01 | 9.60E-05 | 3.57E-03 | 9.48E-05 | 2.34E-10 | 2.84E-06 | 1.20E-04 | 5.53E-04 | 6.67E-01 | 7.86E-01 |
| **METAP1D** | 1.00E+00 | 6.45E-02 | 4.07E-04 | 4.85E-04 | 6.51E-02 | 1.52E-01 | 4.75E-01 | 2.13E-02 | 1.31E-01 | 3.64E-02 | 3.10E-04 | 7.29E-03 | 3.60E-02 | 1.43E-01 | 5.97E-01 | 5.72E-01 |
| **CD5** | 1.00E+00 | 1.57E-03 | 8.11E-04 | 1.84E-04 | 7.33E-04 | 1.54E-02 | 1.65E-03 | 1.36E-01 | 3.70E-01 | 3.07E-01 | 4.57E-01 | 3.26E-01 | 7.29E-01 | 1.64E-01 | 4.68E-02 | 3.89E-01 |
| **CLTA** | 1.00E+00 | 1.32E-02 | 3.09E-03 | 2.88E-04 | 1.13E-01 | 3.00E-01 | 9.17E-01 | 3.85E-03 | 7.53E-01 | 6.21E-01 | 8.11E-01 | 5.80E-01 | 5.05E-01 | 8.17E-01 | 8.48E-02 | 1.12E-01 |
| **SEMA4D** | 1.00E+00 | 6.25E-01 | 6.04E-01 | 9.87E-01 | 1.80E-01 | 4.48E-03 | 1.78E-01 | 5.78E-03 | 2.37E-01 | 3.37E-01 | 3.26E-02 | 3.27E-01 | 4.46E-02 | 1.16E-01 | 4.68E-02 | 7.30E-02 |

**b.**

|  | **Responder (N=5)** | | | | | | | | | | | | | | | |
| --- | --- | --- | --- | --- | --- | --- | --- | --- | --- | --- | --- | --- | --- | --- | --- | --- |
| **Treatment^a^** | **Bepirovirsen** | | | | | | **Peg-IFN** | | | | | | **Off-treatment** | | | |
| **Week** | **Baseline** | **Week 3** | **Week 5** | **Week 8** | **Week 12** | **Week 24** | **Week 1** | **Week 2** | **Week 4** | **Week 6** | **Week 13** | **Week 20** | **Week 1** | **Week 4** | **Week 12** | **Week 24** |
| **C19orf12** | 1.00E+00 | 2.78E-01 | 2.71E-01 | 1.04E-14 | 1.11E-05 | 3.58E-01 | 6.84E-01 | 1.65E-01 | 2.28E-02 | 2.68E-03 | 8.48E-03 | 1.51E-02 | 4.55E-02 | 1.11E-01 | 4.71E-01 | 8.95E-01 |
| **CA5A** | 1.00E+00 | 1.59E-02 | 5.29E-02 | 2.04E-17 | 1.98E-06 | 5.88E-02 | 3.50E-02 | 2.67E-03 | 2.09E-04 | 1.27E-04 | 3.64E-04 | 7.55E-04 | 1.45E-03 | 4.96E-03 | 5.85E-02 | 2.90E-01 |
| **GSTA3** | 1.00E+00 | 1.01E-01 | 4.98E-02 | 4.66E-13 | 1.98E-04 | 6.22E-02 | 2.43E-01 | 2.37E-02 | 6.03E-03 | 6.12E-04 | 6.76E-05 | 1.64E-04 | 3.14E-04 | 1.38E-02 | 2.03E-01 | 3.42E-01 |
| **HAO1** | 1.00E+00 | 3.50E-02 | 1.15E-01 | 2.31E-12 | 5.05E-04 | 2.76E-01 | 6.00E-01 | 1.81E-03 | 9.27E-05 | 6.18E-04 | 2.41E-05 | 2.86E-05 | 1.93E-05 | 1.27E-03 | 3.85E-02 | 2.23E-01 |
| **KRT18** | 1.00E+00 | 2.37E-01 | 2.39E-01 | 3.34E-11 | 5.80E-04 | 7.17E-01 | 8.36E-01 | 8.41E-02 | 7.75E-03 | 1.03E-03 | 1.94E-04 | 1.14E-04 | 1.38E-04 | 2.07E-02 | 5.43E-01 | 9.14E-01 |
| **SULT2A1** | 1.00E+00 | 6.69E-01 | 8.35E-01 | 7.70E-09 | 1.09E-02 | 7.20E-01 | 6.42E-01 | 2.17E-01 | 6.73E-02 | 6.15E-03 | 2.56E-04 | 8.36E-03 | 6.84E-04 | 2.92E-02 | 6.81E-01 | 8.11E-01 |
| **TYMP** | 1.00E+00 | 5.03E-01 | 8.98E-01 | 1.71E-03 | 2.55E-01 | 5.31E-01 | 6.28E-01 | 8.79E-04 | 6.37E-07 | 3.79E-06 | 4.69E-07 | 3.64E-08 | 8.87E-06 | 4.97E-03 | 4.38E-01 | 2.46E-01 |
| **PTS** | 1.00E+00 | 6.14E-02 | 2.62E-01 | 5.82E-11 | 2.15E-03 | 5.73E-01 | 5.41E-01 | 2.71E-01 | 4.77E-03 | 4.66E-03 | 4.59E-03 | 4.99E-03 | 2.44E-02 | 4.63E-01 | 6.99E-01 | 7.15E-01 |
| **SORD** | 1.00E+00 | 5.67E-01 | 3.68E-01 | 4.93E-11 | 3.99E-02 | 8.28E-01 | 9.91E-01 | 5.51E-02 | 6.51E-03 | 4.83E-03 | 5.78E-04 | 3.26E-04 | 2.25E-03 | 1.42E-01 | 8.31E-01 | 7.12E-01 |
| **SHMT1** | 1.00E+00 | 8.73E-01 | 1.68E-01 | 1.39E-06 | 1.48E-01 | 6.98E-01 | 3.16E-01 | 2.32E-01 | 1.71E-02 | 1.71E-02 | 2.04E-03 | 2.15E-04 | 1.14E-02 | 1.81E-01 | 6.02E-01 | 2.19E-01 |
| **AIFM1** | 1.00E+00 | 8.56E-01 | 1.86E-01 | 4.86E-08 | 5.68E-02 | 7.46E-01 | 2.47E-01 | 7.59E-01 | 3.39E-01 | 7.76E-02 | 8.90E-03 | 3.24E-03 | 1.13E-02 | 1.56E-01 | 4.98E-01 | 8.08E-01 |
| **ACAA1** | 1.00E+00 | 5.62E-01 | 5.11E-01 | 3.76E-07 | 3.08E-02 | 8.96E-01 | 9.49E-01 | 7.41E-02 | 1.14E-02 | 4.72E-02 | 1.02E-02 | 4.15E-02 | 8.01E-04 | 3.02E-02 | 1.31E-01 | 6.53E-01 |
| **AGXT** | 1.00E+00 | 9.38E-02 | 2.12E-01 | 5.35E-10 | 1.25E-03 | 4.84E-01 | 7.80E-01 | 5.00E-03 | 1.19E-04 | 2.17E-04 | 1.31E-06 | 5.12E-06 | 2.36E-07 | 7.80E-04 | 3.23E-02 | 2.90E-01 |
| **GRPEL1** | 1.00E+00 | 2.70E-01 | 2.50E-01 | 5.31E-10 | 1.72E-02 | 8.35E-01 | 6.36E-01 | 4.60E-02 | 3.08E-03 | 4.44E-04 | 1.89E-04 | 1.15E-04 | 8.42E-05 | 1.26E-02 | 1.47E-01 | 6.51E-01 |
| **HNMT** | 1.00E+00 | 2.39E-01 | 5.06E-01 | 3.87E-13 | 1.53E-04 | 6.30E-01 | 5.53E-01 | 2.58E-02 | 1.23E-02 | 3.13E-03 | 4.19E-03 | 1.41E-03 | 1.82E-02 | 1.14E-01 | 6.67E-01 | 7.39E-01 |
| **PBLD** | 1.00E+00 | 7.86E-01 | 3.77E-01 | 6.49E-11 | 1.33E-02 | 9.12E-01 | 1.47E-01 | 5.61E-01 | 1.78E-01 | 1.39E-01 | 8.87E-02 | 6.39E-02 | 2.84E-01 | 2.64E-01 | 7.85E-01 | 4.74E-01 |
| **ALDH1A1** | 1.00E+00 | 8.73E-01 | 5.03E-01 | 1.23E-05 | 2.32E-01 | 3.66E-01 | 5.81E-01 | 7.51E-01 | 2.53E-01 | 2.44E-01 | 1.17E-01 | 6.78E-03 | 7.62E-02 | 6.13E-01 | 9.98E-01 | 1.64E-01 |
| **SCLY** | 1.00E+00 | 3.35E-01 | 1.14E-01 | 2.15E-17 | 3.24E-04 | 8.51E-01 | 4.46E-01 | 2.07E-01 | 1.81E-03 | 1.23E-04 | 5.82E-06 | 4.74E-06 | 3.90E-05 | 6.29E-03 | 3.43E-01 | 8.79E-01 |
| **DCXR** | 1.00E+00 | 5.53E-01 | 1.54E-01 | 1.63E-15 | 1.29E-03 | 5.22E-01 | 7.96E-01 | 1.35E-01 | 1.25E-02 | 6.50E-04 | 2.07E-04 | 5.56E-04 | 1.19E-03 | 6.81E-02 | 6.98E-01 | 5.16E-01 |
| **GFER** | 1.00E+00 | 7.18E-01 | 8.27E-01 | 1.76E-10 | 1.32E-02 | 4.20E-01 | 2.87E-01 | 2.78E-01 | 6.96E-02 | 1.27E-02 | 1.70E-03 | 1.46E-02 | 2.80E-03 | 7.90E-02 | 8.04E-01 | 5.24E-01 |
| **FABP1** | 1.00E+00 | 3.33E-01 | 9.81E-01 | 2.29E-05 | 2.70E-02 | 6.74E-01 | 9.67E-01 | 5.10E-01 | 8.43E-02 | 1.72E-02 | 1.09E-02 | 3.71E-02 | 1.10E-02 | 2.90E-01 | 6.08E-01 | 7.91E-01 |
| **FBP1** | 1.00E+00 | 4.46E-01 | 5.50E-01 | 4.20E-06 | 8.29E-04 | 5.37E-01 | 4.89E-01 | 7.78E-02 | 1.81E-02 | 1.57E-02 | 6.99E-02 | 1.60E-02 | 1.03E-02 | 1.37E-02 | 4.58E-01 | 9.66E-01 |
| **ADH4** | 1.00E+00 | 8.71E-02 | 6.67E-02 | 1.03E-10 | 1.10E-03 | 4.34E-01 | 9.40E-01 | 3.07E-01 | 6.64E-02 | 1.54E-01 | 2.78E-02 | 6.30E-02 | 5.84E-02 | 1.64E-01 | 3.64E-01 | 6.03E-01 |
| **KYNU** | 1.00E+00 | 5.17E-01 | 5.85E-01 | 5.02E-12 | 7.85E-03 | 9.43E-01 | 7.23E-01 | 8.99E-02 | 4.78E-03 | 1.53E-03 | 5.49E-04 | 8.57E-04 | 7.52E-04 | 6.11E-02 | 9.96E-01 | 8.57E-01 |
| **ACY1** | 1.00E+00 | 9.81E-01 | 7.52E-01 | 4.75E-10 | 1.21E-02 | 5.19E-01 | 3.88E-01 | 2.96E-01 | 8.88E-02 | 2.63E-02 | 1.47E-02 | 2.89E-02 | 6.15E-02 | 4.48E-01 | 9.26E-01 | 8.05E-01 |
| **HTRA2** | 1.00E+00 | 9.96E-01 | 2.14E-01 | 4.21E-12 | 1.26E-03 | 4.76E-01 | 9.37E-01 | 1.79E-01 | 1.64E-02 | 3.20E-03 | 5.18E-04 | 8.32E-04 | 6.94E-03 | 8.13E-02 | 9.23E-01 | 4.43E-01 |
| **RBP5** | 1.00E+00 | 3.32E-01 | 2.46E-01 | 9.29E-13 | 6.84E-03 | 8.65E-01 | 8.59E-01 | 2.28E-01 | 6.40E-01 | 7.03E-02 | 1.40E-02 | 2.52E-03 | 1.49E-02 | 1.34E-01 | 8.67E-01 | 9.33E-01 |
| **GSTA1** | 1.00E+00 | 4.13E-01 | 2.88E-01 | 9.24E-11 | 9.87E-03 | 5.87E-01 | 3.67E-01 | 5.19E-01 | 6.46E-02 | 7.86E-03 | 8.04E-04 | 1.46E-03 | 1.51E-02 | 1.71E-01 | 7.81E-01 | 4.85E-01 |
| **CXCL17** | 1.00E+00 | 6.53E-01 | 8.70E-01 | 8.75E-01 | 3.60E-01 | 6.92E-02 | 8.89E-01 | 9.60E-02 | 2.99E-02 | 1.17E-02 | 1.43E-02 | 9.57E-03 | 3.21E-03 | 5.30E-02 | 4.46E-01 | 1.41E-01 |
| **VWA1** | 1.00E+00 | 6.32E-02 | 2.51E-06 | 7.32E-01 | 1.30E-01 | 4.13E-02 | 3.14E-01 | 8.83E-01 | 5.11E-02 | 5.10E-02 | 5.73E-02 | 9.30E-02 | 2.32E-01 | 2.91E-01 | 7.35E-01 | 7.90E-01 |
| **GPNMB** | 1.00E+00 | 5.99E-03 | 5.02E-03 | 2.97E-08 | 1.05E-08 | 8.86E-02 | 5.03E-01 | 4.56E-01 | 4.53E-01 | 3.28E-01 | 2.34E-03 | 1.24E-01 | 7.75E-02 | 1.92E-01 | 8.06E-01 | 6.72E-01 |
| **NTF4** | 1.00E+00 | 5.29E-02 | 3.61E-01 | 3.87E-02 | 4.94E-02 | 2.05E-02 | 5.20E-02 | 1.43E-01 | 7.90E-01 | 5.77E-01 | 9.94E-01 | 3.73E-01 | 6.73E-01 | 8.48E-01 | 1.93E-01 | 3.21E-01 |
| **RAB6B** | 1.00E+00 | 6.57E-01 | 2.27E-01 | 2.98E-03 | 8.27E-04 | 4.86E-02 | 4.75E-02 | 7.08E-02 | 2.91E-01 | 3.86E-01 | 6.20E-01 | 3.91E-01 | 3.20E-01 | 1.75E-01 | 1.90E-01 | 6.47E-01 |
| **MVK** | 1.00E+00 | 6.44E-01 | 2.74E-01 | 6.51E-07 | 1.07E-01 | 7.34E-01 | 6.31E-01 | 9.43E-01 | 7.85E-01 | 7.61E-01 | 8.55E-01 | 1.51E-01 | 7.79E-01 | 8.02E-01 | 8.14E-01 | 2.91E-01 |
| **THOP1** | 1.00E+00 | 9.78E-01 | 4.66E-01 | 1.16E-05 | 2.10E-01 | 4.71E-01 | 9.41E-01 | 8.20E-01 | 2.73E-01 | 2.39E-01 | 1.50E-01 | 1.31E-02 | 1.62E-01 | 6.41E-01 | 8.34E-01 | 3.85E-01 |
| **DDAH1** | 1.00E+00 | 8.15E-01 | 2.44E-01 | 2.61E-07 | 1.06E-01 | 8.63E-01 | 2.49E-01 | 9.67E-01 | 2.15E-01 | 1.76E-01 | 2.00E-01 | 1.69E-02 | 1.25E-01 | 6.88E-01 | 3.46E-01 | 5.43E-01 |
| **RBKS** | 1.00E+00 | 9.48E-01 | 3.94E-01 | 1.61E-03 | 4.46E-01 | 4.91E-01 | 4.13E-01 | 9.53E-01 | 5.40E-01 | 5.19E-01 | 4.23E-01 | 4.35E-02 | 5.09E-01 | 8.84E-01 | 7.33E-01 | 2.14E-01 |
| **METAP1D** | 1.00E+00 | 5.28E-01 | 2.35E-01 | 2.12E-05 | 8.20E-02 | 3.63E-01 | 9.00E-01 | 2.10E-01 | 7.94E-01 | 3.02E-01 | 2.90E-01 | 2.24E-01 | 8.02E-02 | 2.77E-01 | 4.03E-01 | 9.17E-01 |
| **CD5** | 1.00E+00 | 4.94E-01 | 6.93E-04 | 2.39E-01 | 8.72E-01 | 5.56E-01 | 7.81E-01 | 1.14E-01 | 8.52E-01 | 8.46E-01 | 4.40E-01 | 8.28E-01 | 5.38E-01 | 5.52E-01 | 4.58E-01 | 8.46E-01 |
| **CLTA** | 1.00E+00 | 5.33E-01 | 7.48E-01 | 2.44E-01 | 1.10E-01 | 1.23E-01 | 1.11E-01 | 2.66E-01 | 9.26E-02 | 7.33E-02 | 1.17E-02 | 3.01E-01 | 2.16E-01 | 4.34E-01 | 6.88E-01 | 9.36E-01 |
| **SEMA4D** | 1.00E+00 | 3.23E-02 | 8.83E-06 | 1.29E-02 | 1.13E-01 | 1.84E-03 | 7.16E-03 | 7.79E-03 | 6.87E-02 | 1.95E-02 | 3.43E-02 | 1.45E-01 | 1.41E-02 | 1.98E-01 | 2.74E-01 | 1.35E-02 |

**c.**

|  | **Non-responder (N=37)** | | | | | | | | | | | | | | |
| --- | --- | --- | --- | --- | --- | --- | --- | --- | --- | --- | --- | --- | --- | --- | --- |
| **Treatment^a^** | **Bepirovirsen** | | | | | **Peg-IFN** | | | | | | **Off-treatment** | | | |
| **Week** | **Baseline** | **Week 3** | **Week 5** | **Week 8** | **Week 12** | **Week 1** | **Week 2** | **Week 4** | **Week 6** | **Week 13** | **Week 20** | **Week 1** | **Week 4** | **Week 12** | **Week 24** |
| **C19orf12** | 1.00E+00 | 1.97E-01 | 6.72E-04 | 2.00E-07 | 1.23E-06 | 4.27E-04 | 1.63E-08 | 1.40E-13 | 7.49E-13 | 1.35E-13 | 9.39E-11 | 2.59E-11 | 2.45E-03 | 7.05E-01 | 1.64E-02 |
| **CA5A** | 1.00E+00 | 1.17E-01 | 1.16E-03 | 8.28E-05 | 3.30E-05 | 4.77E-03 | 8.64E-05 | 1.36E-07 | 8.74E-07 | 2.19E-06 | 2.26E-07 | 5.76E-07 | 2.25E-02 | 1.30E-01 | 7.64E-03 |
| **GSTA3** | 1.00E+00 | 2.97E-01 | 1.84E-03 | 6.87E-04 | 2.77E-03 | 1.00E-01 | 8.15E-04 | 6.46E-07 | 5.02E-10 | 3.05E-10 | 5.58E-10 | 6.74E-10 | 7.68E-02 | 1.31E-01 | 1.12E-02 |
| **HAO1** | 1.00E+00 | 8.41E-01 | 4.01E-01 | 7.12E-02 | 1.25E-01 | 2.45E-01 | 4.04E-03 | 4.52E-06 | 5.23E-07 | 2.17E-07 | 2.01E-06 | 2.08E-08 | 1.79E-02 | 2.18E-01 | 9.51E-03 |
| **KRT18** | 1.00E+00 | 7.57E-01 | 1.16E-02 | 2.46E-05 | 2.46E-04 | 3.61E-02 | 3.09E-05 | 8.51E-10 | 2.66E-13 | 9.38E-15 | 2.54E-15 | 3.88E-14 | 3.85E-05 | 4.82E-01 | 9.28E-03 |
| **SULT2A1** | 1.00E+00 | 8.86E-01 | 1.15E-01 | 5.68E-03 | 3.22E-02 | 1.42E-01 | 2.69E-04 | 1.61E-07 | 1.85E-10 | 3.04E-10 | 1.37E-11 | 5.34E-13 | 7.77E-05 | 8.28E-01 | 2.24E-01 |
| **TYMP** | 1.00E+00 | 7.59E-01 | 2.39E-01 | 1.77E-01 | 6.07E-02 | 8.31E-01 | 2.22E-15 | 1.90E-27 | 1.21E-34 | 2.04E-34 | 1.49E-36 | 1.48E-36 | 4.69E-13 | 2.47E-01 | 1.34E-01 |
| **PTS** | 1.00E+00 | 9.06E-02 | 8.73E-03 | 2.61E-03 | 4.43E-03 | 2.47E-01 | 8.29E-06 | 1.44E-11 | 5.84E-12 | 8.51E-09 | 9.38E-11 | 3.30E-09 | 6.96E-02 | 3.01E-01 | 1.85E-02 |
| **SORD** | 1.00E+00 | 9.88E-02 | 9.42E-01 | 6.86E-01 | 7.17E-01 | 3.32E-01 | 6.71E-04 | 5.99E-09 | 1.47E-09 | 1.25E-07 | 1.99E-10 | 4.03E-09 | 3.02E-01 | 1.18E-03 | 1.06E-04 |
| **SHMT1** | 1.00E+00 | 1.22E-01 | 8.07E-01 | 6.08E-01 | 5.32E-01 | 1.17E-03 | 1.42E-02 | 2.85E-06 | 1.29E-08 | 1.49E-07 | 3.34E-11 | 1.51E-07 | 4.81E-01 | 1.00E-03 | 9.84E-04 |
| **AIFM1** | 1.00E+00 | 1.05E-01 | 9.89E-01 | 4.06E-01 | 6.39E-01 | 8.96E-02 | 7.59E-01 | 2.59E-02 | 7.87E-03 | 2.02E-04 | 8.14E-06 | 3.46E-05 | 9.50E-01 | 2.60E-03 | 1.18E-04 |
| **ACAA1** | 1.00E+00 | 9.19E-01 | 7.79E-01 | 1.62E-01 | 8.38E-01 | 8.85E-01 | 1.05E-01 | 2.51E-03 | 6.64E-04 | 3.89E-03 | 1.48E-04 | 8.60E-07 | 1.79E-01 | 2.97E-01 | 3.47E-02 |
| **AGXT** | 1.00E+00 | 5.98E-01 | 6.07E-01 | 1.91E-01 | 4.99E-01 | 7.41E-01 | 2.48E-02 | 6.18E-05 | 5.90E-06 | 9.34E-07 | 1.35E-07 | 3.10E-10 | 5.98E-03 | 1.61E-01 | 1.38E-02 |
| **GRPEL1** | 1.00E+00 | 7.01E-01 | 8.54E-01 | 8.50E-01 | 5.34E-01 | 5.50E-01 | 6.65E-03 | 5.93E-05 | 1.24E-06 | 1.07E-06 | 4.11E-10 | 1.87E-14 | 1.12E-02 | 2.53E-02 | 1.79E-03 |
| **HNMT** | 1.00E+00 | 5.19E-01 | 2.02E-02 | 1.10E-05 | 8.22E-04 | 1.15E-01 | 2.93E-05 | 5.64E-09 | 2.15E-09 | 1.65E-07 | 1.55E-07 | 3.20E-08 | 3.28E-03 | 3.77E-01 | 1.11E-01 |
| **PBLD** | 1.00E+00 | 7.02E-01 | 1.07E-02 | 2.12E-03 | 1.60E-02 | 7.29E-01 | 1.77E-02 | 6.12E-06 | 4.39E-06 | 2.22E-06 | 4.41E-06 | 3.56E-05 | 4.06E-01 | 8.06E-02 | 6.19E-02 |
| **ALDH1A1** | 1.00E+00 | 1.89E-01 | 8.41E-01 | 6.24E-01 | 7.56E-01 | 3.29E-02 | 1.40E-01 | 1.34E-04 | 4.14E-05 | 4.28E-04 | 1.56E-09 | 2.51E-07 | 8.17E-02 | 2.22E-02 | 1.35E-03 |
| **SCLY** | 1.00E+00 | 5.09E-01 | 1.55E-02 | 7.20E-03 | 1.26E-02 | 3.59E-01 | 7.95E-04 | 3.96E-10 | 3.18E-12 | 4.48E-16 | 1.57E-14 | 3.64E-19 | 2.02E-04 | 1.08E-01 | 2.78E-03 |
| **DCXR** | 1.00E+00 | 4.92E-01 | 2.83E-01 | 1.63E-02 | 4.43E-02 | 4.74E-01 | 5.58E-04 | 1.98E-06 | 1.30E-07 | 1.73E-07 | 4.68E-07 | 5.29E-07 | 2.77E-01 | 1.67E-02 | 1.36E-04 |
| **GFER** | 1.00E+00 | 8.29E-01 | 1.13E-01 | 3.94E-03 | 2.14E-02 | 8.51E-01 | 5.30E-04 | 7.68E-07 | 1.31E-08 | 3.27E-11 | 1.26E-11 | 7.37E-13 | 6.54E-03 | 3.19E-01 | 2.46E-02 |
| **FABP1** | 1.00E+00 | 8.91E-01 | 7.24E-01 | 9.79E-01 | 8.82E-01 | 6.18E-01 | 9.29E-02 | 2.98E-04 | 4.80E-05 | 4.19E-05 | 1.85E-07 | 3.08E-07 | 8.04E-03 | 2.76E-02 | 4.40E-02 |
| **FBP1** | 1.00E+00 | 3.69E-01 | 4.09E-01 | 4.94E-01 | 5.11E-01 | 2.19E-01 | 4.24E-01 | 7.97E-04 | 2.56E-04 | 1.62E-03 | 1.05E-04 | 5.26E-07 | 2.12E-01 | 1.14E-01 | 9.71E-02 |
| **ADH4** | 1.00E+00 | 8.42E-01 | 3.34E-02 | 1.68E-02 | 1.50E-02 | 5.45E-01 | 3.21E-02 | 1.63E-03 | 6.64E-04 | 1.67E-04 | 8.09E-04 | 3.45E-04 | 4.74E-01 | 7.51E-02 | 2.55E-04 |
| **KYNU** | 1.00E+00 | 8.90E-01 | 8.16E-02 | 6.46E-03 | 6.51E-02 | 6.60E-01 | 2.34E-04 | 5.12E-09 | 4.00E-11 | 1.87E-11 | 4.69E-10 | 5.35E-13 | 5.26E-03 | 1.21E-01 | 7.78E-04 |
| **ACY1** | 1.00E+00 | 8.37E-01 | 1.13E-01 | 1.83E-02 | 1.20E-02 | 1.99E-01 | 1.57E-03 | 2.30E-07 | 2.71E-08 | 9.50E-09 | 9.14E-07 | 2.15E-09 | 2.57E-02 | 8.33E-02 | 7.94E-04 |
| **HTRA2** | 1.00E+00 | 9.83E-01 | 1.95E-01 | 7.25E-03 | 4.29E-02 | 8.74E-01 | 1.15E-03 | 5.59E-07 | 2.40E-10 | 5.14E-15 | 2.54E-14 | 3.86E-15 | 2.51E-02 | 2.57E-02 | 2.13E-03 |
| **RBP5** | 1.00E+00 | 9.41E-01 | 6.57E-02 | 9.44E-02 | 3.70E-01 | 5.00E-01 | 9.34E-01 | 2.65E-01 | 1.60E-02 | 1.33E-02 | 8.12E-05 | 4.25E-04 | 5.47E-01 | 1.29E-02 | 3.38E-04 |
| **GSTA1** | 1.00E+00 | 2.54E-01 | 4.12E-01 | 4.02E-01 | 1.19E-01 | 5.88E-04 | 2.16E-01 | 4.12E-01 | 9.08E-02 | 6.58E-02 | 1.31E-02 | 3.93E-02 | 1.67E-01 | 4.08E-06 | 6.54E-06 |
| **CXCL17** | 1.00E+00 | 3.28E-02 | 6.73E-02 | 6.24E-02 | 1.38E-01 | 9.13E-01 | 1.82E-04 | 3.42E-05 | 6.16E-06 | 2.00E-05 | 1.85E-08 | 3.82E-09 | 4.66E-04 | 4.52E-03 | 3.27E-02 |
| **VWA1** | 1.00E+00 | 9.08E-02 | 2.05E-01 | 4.64E-01 | 2.40E-01 | 6.97E-02 | 3.30E-07 | 5.45E-10 | 9.67E-12 | 9.22E-10 | 3.76E-10 | 2.79E-16 | 9.64E-10 | 6.58E-02 | 5.57E-01 |
| **GPNMB** | 1.00E+00 | 2.47E-10 | 8.01E-19 | 1.47E-22 | 7.81E-22 | 2.15E-21 | 3.06E-12 | 1.23E-17 | 8.57E-18 | 1.91E-05 | 7.75E-05 | 2.74E-05 | 4.08E-05 | 3.00E-02 | 5.30E-01 |
| **NTF4** | 1.00E+00 | 2.79E-03 | 1.54E-02 | 1.98E-03 | 6.82E-03 | 2.98E-03 | 8.25E-02 | 6.06E-01 | 7.07E-01 | 8.62E-01 | 8.90E-01 | 3.95E-01 | 4.72E-02 | 3.25E-01 | 5.81E-01 |
| **RAB6B** | 1.00E+00 | 2.32E-01 | 5.20E-02 | 2.48E-01 | 2.96E-01 | 3.32E-01 | 6.60E-01 | 6.86E-01 | 2.04E-01 | 6.62E-01 | 5.22E-01 | 7.48E-01 | 4.94E-01 | 3.37E-01 | 8.37E-02 |
| **MVK** | 1.00E+00 | 2.72E-01 | 4.13E-01 | 1.20E-01 | 1.39E-01 | 5.28E-01 | 8.69E-01 | 1.03E-01 | 1.16E-01 | 1.65E-01 | 6.80E-03 | 7.63E-02 | 9.65E-01 | 6.11E-02 | 2.83E-02 |
| **THOP1** | 1.00E+00 | 5.14E-01 | 9.00E-01 | 6.55E-01 | 9.01E-01 | 5.96E-02 | 2.35E-01 | 3.13E-03 | 7.81E-04 | 1.08E-02 | 3.83E-07 | 6.24E-06 | 2.66E-02 | 2.28E-01 | 5.90E-02 |
| **DDAH1** | 1.00E+00 | 1.77E-02 | 7.53E-01 | 7.09E-01 | 9.57E-01 | 2.37E-01 | 7.22E-01 | 5.14E-02 | 1.70E-03 | 2.15E-02 | 1.96E-05 | 5.29E-04 | 9.93E-01 | 8.02E-03 | 1.38E-02 |
| **RBKS** | 1.00E+00 | 7.46E-02 | 5.20E-01 | 5.13E-01 | 2.35E-01 | 1.13E-03 | 6.95E-01 | 7.13E-03 | 6.87E-03 | 2.63E-01 | 4.98E-05 | 1.37E-02 | 6.85E-01 | 2.85E-02 | 6.09E-03 |
| **METAP1D** | 1.00E+00 | 2.52E-01 | 1.92E-01 | 9.54E-01 | 4.34E-01 | 8.74E-02 | 1.47E-01 | 5.94E-01 | 9.08E-01 | 3.04E-01 | 2.59E-01 | 4.68E-01 | 3.51E-01 | 1.77E-04 | 5.78E-05 |
| **CD5** | 1.00E+00 | 7.07E-03 | 3.55E-03 | 4.75E-03 | 8.56E-03 | 1.78E-02 | 9.47E-01 | 9.11E-01 | 2.23E-01 | 5.79E-01 | 7.52E-01 | 8.73E-01 | 6.83E-01 | 2.17E-01 | 5.89E-01 |
| **CLTA** | 1.00E+00 | 9.70E-01 | 6.88E-01 | 1.28E-01 | 8.02E-01 | 4.29E-01 | 6.55E-02 | 1.72E-01 | 1.94E-01 | 1.83E-03 | 4.60E-01 | 6.94E-04 | 9.89E-03 | 1.97E-02 | 1.55E-01 |
| **SEMA4D** | 1.00E+00 | 5.86E-01 | 6.53E-01 | 4.47E-02 | 1.00E-01 | 4.27E-02 | 4.00E-03 | 2.41E-02 | 9.40E-02 | 1.50E-02 | 4.87E-02 | 7.46E-04 | 1.76E-03 | 3.95E-02 | 1.14E-02 |

**d.**

|  | **Responder (N=8)** | | | | | | | | | | | | | | |
| --- | --- | --- | --- | --- | --- | --- | --- | --- | --- | --- | --- | --- | --- | --- | --- |
| **Treatment^a^** | **Bepirovirsen** | | | | | **Peg-IFN** | | | | | | **Off-treatment** | | | |
| **Week** | **Baseline** | **Week 3** | **Week 5** | **Week 8** | **Week 12** | **Week 1** | **Week 2** | **Week 4** | **Week 6** | **Week 13** | **Week 20** | **Week 1** | **Week 4** | **Week 12** | **Week 24** |
| **C19orf12** | 1.00E+00 | 6.36E-01 | 9.35E-02 | 1.06E-05 | 3.01E-04 | 3.23E-03 | 2.08E-02 | 3.01E-04 | 5.09E-03 | 5.91E-04 | 1.31E-02 | 2.89E-02 | 2.49E-01 | 7.41E-01 | 5.37E-01 |
| **CA5A** | 1.00E+00 | 7.72E-01 | 5.50E-01 | 6.04E-03 | 4.31E-02 | 1.40E-01 | 5.94E-02 | 4.86E-02 | 2.91E-02 | 9.48E-03 | 1.63E-01 | 9.10E-02 | 3.68E-01 | 8.63E-01 | 4.32E-01 |
| **GSTA3** | 1.00E+00 | 9.28E-01 | 9.79E-02 | 7.65E-05 | 4.24E-02 | 6.33E-02 | 2.74E-01 | 1.12E-02 | 2.69E-03 | 1.89E-03 | 1.41E-02 | 4.22E-02 | 2.89E-01 | 6.84E-01 | 4.19E-01 |
| **HAO1** | 1.00E+00 | 1.63E-01 | 5.81E-01 | 1.82E-01 | 4.35E-01 | 1.42E-01 | 3.26E-01 | 1.21E-01 | 1.26E-01 | 2.38E-01 | 4.71E-01 | 3.58E-01 | 8.81E-01 | 4.55E-01 | 1.53E-01 |
| **KRT18** | 1.00E+00 | 4.70E-01 | 9.53E-02 | 3.57E-04 | 1.95E-02 | 1.37E-02 | 2.39E-01 | 1.56E-03 | 9.99E-04 | 3.29E-06 | 7.16E-05 | 6.13E-05 | 2.30E-02 | 3.12E-01 | 6.67E-01 |
| **SULT2A1** | 1.00E+00 | 9.92E-01 | 4.90E-01 | 3.18E-03 | 3.93E-02 | 1.10E-01 | 1.72E-01 | 2.94E-03 | 8.89E-04 | 1.27E-06 | 3.23E-04 | 1.50E-04 | 2.37E-02 | 7.49E-02 | 8.34E-01 |
| **TYMP** | 1.00E+00 | 7.24E-01 | 8.59E-01 | 1.02E-01 | 6.38E-01 | 9.21E-01 | 3.62E-04 | 1.99E-09 | 4.19E-08 | 8.91E-13 | 1.62E-10 | 2.14E-12 | 1.73E-03 | 8.62E-01 | 2.15E-01 |
| **PTS** | 1.00E+00 | 6.43E-01 | 7.96E-01 | 1.35E-02 | 4.43E-02 | 4.64E-01 | 1.18E-01 | 2.48E-02 | 2.52E-02 | 5.35E-03 | 1.51E-01 | 9.82E-02 | 6.62E-01 | 5.69E-01 | 1.11E-01 |
| **SORD** | 1.00E+00 | 1.50E-01 | 4.67E-01 | 1.66E-01 | 7.56E-01 | 7.97E-01 | 1.92E-01 | 4.07E-02 | 1.18E-01 | 3.49E-03 | 1.04E-01 | 1.22E-01 | 7.69E-01 | 3.87E-01 | 2.20E-03 |
| **SHMT1** | 1.00E+00 | 7.45E-01 | 3.37E-01 | 5.54E-03 | 1.28E-01 | 7.58E-01 | 9.25E-03 | 2.09E-03 | 2.61E-03 | 1.24E-04 | 5.20E-03 | 6.05E-03 | 1.26E-01 | 8.99E-01 | 4.41E-01 |
| **AIFM1** | 1.00E+00 | 6.54E-01 | 9.62E-01 | 8.52E-02 | 4.15E-01 | 8.31E-01 | 8.92E-01 | 5.85E-01 | 1.54E-01 | 2.74E-03 | 2.94E-02 | 1.60E-01 | 1.43E-01 | 6.98E-01 | 1.55E-01 |
| **ACAA1** | 1.00E+00 | 1.38E-01 | 2.43E-01 | 5.45E-01 | 8.41E-01 | 4.69E-01 | 4.14E-01 | 6.25E-02 | 1.25E-01 | 1.63E-01 | 1.25E-01 | 2.80E-01 | 2.25E-01 | 5.02E-01 | 3.99E-01 |
| **AGXT** | 1.00E+00 | 5.59E-02 | 4.32E-01 | 4.12E-01 | 8.01E-01 | 5.81E-01 | 6.62E-01 | 1.56E-01 | 1.15E-01 | 2.79E-01 | 3.48E-01 | 2.93E-01 | 9.77E-01 | 3.98E-01 | 5.53E-02 |
| **GRPEL1** | 1.00E+00 | 5.69E-02 | 8.98E-02 | 6.11E-01 | 3.60E-01 | 6.15E-01 | 3.80E-01 | 3.33E-01 | 2.91E-01 | 1.07E-02 | 4.25E-02 | 1.92E-02 | 4.21E-01 | 4.57E-01 | 4.98E-03 |
| **HNMT** | 1.00E+00 | 4.91E-01 | 8.97E-02 | 2.75E-05 | 4.78E-02 | 7.78E-03 | 1.80E-02 | 2.17E-04 | 8.50E-04 | 2.11E-05 | 1.09E-03 | 1.76E-04 | 6.93E-02 | 2.22E-01 | 7.89E-01 |
| **PBLD** | 1.00E+00 | 7.96E-01 | 5.22E-02 | 1.63E-06 | 2.87E-02 | 3.64E-02 | 9.87E-02 | 1.92E-02 | 1.26E-03 | 2.37E-03 | 2.43E-02 | 7.56E-03 | 1.50E-01 | 3.39E-01 | 4.95E-01 |
| **ALDH1A1** | 1.00E+00 | 7.97E-01 | 9.92E-01 | 2.07E-02 | 3.42E-01 | 4.69E-01 | 1.46E-02 | 1.01E-02 | 1.94E-02 | 5.83E-04 | 6.15E-03 | 3.73E-03 | 1.10E-01 | 3.91E-01 | 4.08E-01 |
| **SCLY** | 1.00E+00 | 6.30E-01 | 6.21E-01 | 1.27E-04 | 1.69E-02 | 3.24E-02 | 5.31E-02 | 5.22E-04 | 3.76E-03 | 5.17E-05 | 7.94E-04 | 4.38E-03 | 1.02E-01 | 7.23E-01 | 2.12E-01 |
| **DCXR** | 1.00E+00 | 8.13E-01 | 9.04E-01 | 3.99E-04 | 1.43E-02 | 7.85E-02 | 1.13E-02 | 2.45E-03 | 3.20E-02 | 5.59E-04 | 1.23E-02 | 2.60E-02 | 2.29E-01 | 3.03E-01 | 3.06E-01 |
| **GFER** | 1.00E+00 | 8.20E-01 | 6.91E-01 | 3.06E-04 | 1.31E-01 | 1.99E-01 | 6.40E-02 | 1.59E-03 | 3.80E-03 | 1.56E-06 | 4.28E-05 | 1.11E-04 | 1.46E-02 | 1.16E-01 | 8.56E-01 |
| **FABP1** | 1.00E+00 | 4.56E-01 | 7.61E-01 | 1.82E-02 | 3.13E-01 | 1.25E-01 | 3.29E-01 | 7.30E-03 | 9.36E-03 | 4.10E-05 | 1.71E-03 | 7.02E-04 | 2.35E-01 | 5.87E-01 | 6.55E-01 |
| **FBP1** | 1.00E+00 | 8.14E-01 | 5.40E-01 | 2.75E-01 | 5.74E-01 | 7.23E-01 | 8.20E-01 | 4.66E-01 | 2.04E-01 | 2.03E-02 | 1.40E-03 | 1.92E-02 | 7.13E-01 | 9.55E-01 | 2.49E-01 |
| **ADH4** | 1.00E+00 | 5.23E-01 | 5.36E-01 | 9.64E-03 | 7.43E-02 | 1.51E-01 | 3.19E-01 | 1.75E-01 | 1.79E-01 | 1.04E-01 | 2.69E-01 | 4.36E-01 | 5.46E-01 | 9.89E-01 | 1.04E-01 |
| **KYNU** | 1.00E+00 | 9.37E-01 | 5.35E-01 | 6.62E-04 | 1.72E-02 | 4.30E-02 | 4.62E-02 | 2.21E-04 | 2.15E-03 | 4.74E-05 | 8.87E-04 | 2.30E-04 | 9.67E-02 | 1.54E-01 | 4.20E-01 |
| **ACY1** | 1.00E+00 | 2.81E-01 | 5.78E-01 | 1.77E-03 | 2.38E-02 | 5.23E-02 | 1.50E-01 | 4.60E-03 | 5.58E-02 | 3.62E-03 | 1.15E-02 | 1.36E-02 | 2.53E-01 | 8.18E-01 | 7.00E-01 |
| **HTRA2** | 1.00E+00 | 7.44E-01 | 6.97E-01 | 8.34E-03 | 6.48E-01 | 9.16E-01 | 2.66E-01 | 2.29E-02 | 1.78E-01 | 8.89E-05 | 9.67E-03 | 7.88E-03 | 7.20E-01 | 3.85E-01 | 1.46E-01 |
| **RBP5** | 1.00E+00 | 9.27E-01 | 2.93E-01 | 3.35E-03 | 1.90E-01 | 7.33E-02 | 5.61E-01 | 7.45E-02 | 9.65E-02 | 1.13E-02 | 4.74E-02 | 2.23E-02 | 1.73E-01 | 6.97E-01 | 4.69E-01 |
| **GSTA1** | 1.00E+00 | 4.17E-01 | 4.97E-01 | 4.50E-03 | 2.38E-01 | 6.06E-01 | 9.05E-01 | 6.79E-02 | 3.09E-02 | 1.08E-02 | 9.38E-02 | 1.13E-01 | 9.76E-01 | 3.52E-01 | 9.11E-02 |
| **CXCL17** | 1.00E+00 | 2.16E-03 | 1.26E-04 | 1.09E-04 | 1.26E-03 | 3.98E-04 | 2.67E-05 | 1.31E-09 | 1.53E-09 | 1.11E-09 | 1.04E-09 | 3.69E-08 | 7.64E-07 | 4.19E-04 | 2.96E-03 |
| **VWA1** | 1.00E+00 | 2.59E-01 | 4.68E-02 | 5.57E-01 | 1.30E-01 | 1.99E-01 | 7.70E-01 | 4.15E-01 | 3.62E-01 | 2.23E-01 | 6.19E-01 | 8.79E-02 | 2.86E-01 | 3.83E-01 | 2.43E-01 |
| **GPNMB** | 1.00E+00 | 6.70E-02 | 1.07E-03 | 9.58E-06 | 1.18E-03 | 1.02E-04 | 8.49E-02 | 5.40E-05 | 2.12E-03 | 1.44E-03 | 2.19E-01 | 1.77E-01 | 4.75E-01 | 9.36E-01 | 5.42E-01 |
| **NTF4** | 1.00E+00 | 2.43E-01 | 5.96E-01 | 1.99E-01 | 5.14E-01 | 1.73E-01 | 4.88E-01 | 1.76E-01 | 8.62E-01 | 5.51E-01 | 8.61E-01 | 9.12E-01 | 9.25E-01 | 3.35E-01 | 7.87E-01 |
| **RAB6B** | 1.00E+00 | 4.91E-02 | 7.65E-01 | 6.01E-01 | 9.28E-01 | 9.34E-01 | 8.48E-01 | 7.80E-01 | 7.36E-01 | 4.42E-01 | 7.56E-01 | 9.98E-01 | 8.24E-01 | 9.33E-01 | 5.58E-01 |
| **MVK** | 1.00E+00 | 5.66E-01 | 7.10E-01 | 2.49E-02 | 2.58E-01 | 6.04E-01 | 9.50E-01 | 6.63E-01 | 7.74E-01 | 2.19E-01 | 9.71E-01 | 5.76E-01 | 4.66E-01 | 8.31E-01 | 4.12E-01 |
| **THOP1** | 1.00E+00 | 4.89E-01 | 5.24E-01 | 3.42E-03 | 4.04E-01 | 4.40E-01 | 7.94E-02 | 2.12E-02 | 7.98E-02 | 5.77E-04 | 1.69E-02 | 1.74E-02 | 3.16E-01 | 4.51E-01 | 8.29E-01 |
| **DDAH1** | 1.00E+00 | 4.43E-01 | 6.57E-01 | 1.22E-02 | 6.69E-01 | 7.05E-01 | 9.96E-01 | 1.10E-01 | 8.59E-02 | 2.15E-02 | 7.73E-02 | 1.31E-01 | 8.71E-01 | 7.92E-01 | 1.87E-01 |
| **RBKS** | 1.00E+00 | 5.57E-01 | 6.73E-01 | 1.55E-01 | 9.20E-01 | 8.78E-01 | 1.29E-01 | 1.85E-01 | 2.68E-01 | 9.96E-02 | 2.15E-01 | 1.11E-01 | 8.00E-01 | 8.51E-01 | 2.57E-01 |
| **METAP1D** | 1.00E+00 | 5.83E-01 | 4.66E-01 | 3.52E-01 | 8.34E-01 | 9.81E-01 | 9.73E-01 | 6.81E-01 | 5.92E-01 | 3.45E-01 | 6.62E-01 | 6.55E-01 | 6.73E-01 | 3.25E-01 | 1.48E-01 |
| **CD5** | 1.00E+00 | 9.52E-01 | 7.84E-01 | 1.83E-02 | 1.81E-01 | 5.00E-01 | 4.15E-01 | 1.06E-01 | 8.09E-01 | 9.85E-01 | 2.35E-01 | 7.61E-01 | 8.03E-01 | 8.24E-01 | 6.48E-01 |
| **CLTA** | 1.00E+00 | 2.74E-01 | 7.42E-01 | 8.15E-01 | 9.09E-01 | 8.24E-01 | 7.82E-01 | 6.85E-01 | 3.67E-01 | 3.87E-01 | 1.37E-01 | 5.38E-01 | 6.56E-01 | 5.71E-01 | 4.30E-01 |
| **SEMA4D** | 1.00E+00 | 5.72E-01 | 4.52E-01 | 2.18E-01 | 5.30E-01 | 4.11E-01 | 3.37E-01 | 6.84E-02 | 6.70E-01 | 5.15E-01 | 1.30E-01 | 5.26E-01 | 6.67E-01 | 8.00E-02 | 3.91E-01 |

Table cells color coding: purple = p<0.0001; blue = p<0.001; orange = p<0.01; green = p<0.05; no color = non-statistically significant. Red text indicates significantly increased protein expression, and blue text indicates significantly decreased protein expression (see also corresponding heatmap in **Figure 4a**). ^a^Samples taken pre-dose; Week 1 of the bepirovirsen treatment window is before the first bepirovirsen dose (baseline), Week 1 of the Peg-IFN window is before the first Peg-IFN dose, and Week 1 of the off-treatment window is after the last Peg-IFN dose.
Peg-IFN, pegylated interferon-α-2a.

## R code

### Load libraries and previously compiled functions & data

library(ggplot2)

library(tidyr)

library(dplyr)

library(ComplexHeatmap)

library(ggpubr)

library(EnvStats)

library(haven)

load("data4BiomarkerPaper.RData")

### Table 1

# 1) N

table(unique(merged[merged$FASFL=="Y", c("USUBJID", "ACTARMCD")])$ACTARMCD)

nrow(unique(merged[merged$FASFL=="Y", c("USUBJID", "ACTARMCD")]))

# 2) Sex

tmp <- unique(merged[merged$FASFL == "Y", c("USUBJID", "ACTARMCD", "SEX")])

results.sex <- as.data.frame(tmp %>% group_by(ACTARMCD) %>%

summarise(Female = sum(SEX == "F"),

Male = sum(SEX== "M"),

N=length(SEX)))

results.sex <- rbind(results.sex, data.frame(ACTARMCD ="Overall",

Female=sum(tmp$SEX == "F"),

Male=sum(tmp$SEX == "M"),

N=length(tmp$SEX)))

results.sex$Female_percent <- round(results.sex$Female / results.sex$N * 100, 0)

results.sex$Male_percent <- round(results.sex$Male / results.sex$N * 100, 0)

# 3) HBsAG

tmp <- lb[lb$LBTESTCD == "HBSAG" &

lb$PARAM == "SERUM OR PLASMA Hepatitis B Virus Surface Antigen (log10 IU/mL)" &

lb$AVISIT == "BASELINE", c("USUBJID", "AVAL")]

tmp$ACTARMCD <- sl$ACTARMCD[match(tmp$USUBJID, sl$USUBJID)]

tmp <- tmp[tmp$USUBJID %in% merged$USUBJID[merged$FASFL == "Y"], ]

tmp$HBSAGBL <- 10^tmp$AVAL

results.hbsag <- as.data.frame(tmp %>% group_by(ACTARMCD) %>%

summarise(m = round(mean(HBSAGBL, na.rm=FALSE), 1),

sd= round(sd(HBSAGBL, na.rm=FALSE), 1),

N = length(HBSAGBL),

lte1000 = sum(HBSAGBL <= 1000),

gt1000 = sum(HBSAGBL > 1000)))

results.hbsag <- rbind(results.hbsag,

data.frame(ACTARMCD = "Overall",

m=round(mean(tmp$HBSAGBL), 1),

sd=round(sd(tmp$HBSAGBL), 1),

N=length(tmp$HBSAGBL),

lte1000 = sum(tmp$HBSAGBL <= 1000),

gt1000 =sum(tmp$HBSAGBL > 1000)))

results.hbsag$lte1000_percent <- round(results.hbsag$lte1000 /

results.hbsag$N * 100, 0)

results.hbsag$gt1000_percent <- round(results.hbsag$gt1000 /

results.hbsag$N * 100, 0)

# 4) HBV DNA

tmp <- lb[lb$LBTESTCD == "HBVDNA" & lb$AVISIT == "BASELINE",

c("USUBJID", "AVAL", "LBSTRESC")]

tmp$ACTARMCD <- sl$ACTARMCD[match(tmp$USUBJID, sl$USUBJID)]

tmp <- tmp[tmp$USUBJID %in% merged$USUBJID[merged$FASFL == "Y"], ]

results.hbvdna <- as.data.frame(tmp %>% group_by(ACTARMCD) %>%

summarise(ltlloq = sum(AVAL < 20),

N=length(AVAL)))

results.hbvdna <- rbind(results.hbvdna,

data.frame(ACTARMCD="Overall",

ltlloq=sum(tmp$AVAL < 20),

N=length(tmp$AVAL)))

results.hbvdna$ltlloq_percent <- round(results.hbvdna$ltlloq /

results.hbvdna$N * 100, 0)

# 5) ALT

tmp <- lb[lb$LBTESTCD == "ALT" & lb$AVISIT == "BASELINE", c("USUBJID", "AVAL",

"LBSTRESC", "ANRIND")]

tmp$ACTARMCD <- sl$ACTARMCD[match(tmp$USUBJID, sl$USUBJID)]

tmp <- tmp[tmp$USUBJID %in% merged$USUBJID[merged$FASFL == "Y"], ]

results.alt <- as.data.frame(tmp %>% group_by(ACTARMCD) %>%

summarise(lteuln = sum(ANRIND != "HIGH"),

gtuln = sum(ANRIND == "HIGH"),

N=length(ANRIND)))

results.alt <- rbind(results.alt, data.frame(ACTARMCD ="Overall",

lteuln=sum(tmp$ANRIND != "HIGH"),

gtuln=sum(tmp$ANRIND == "HIGH"),

N=length(tmp$ANRIND)))

results.alt$lteuln_percent <- round(results.alt$lteuln / results.alt$N * 100, 0)

results.alt$gtuln_percent <- round(results.alt$gtuln / results.alt$N * 100, 0)

### Fig. 2b

library(org.Hs.egSYMBOL)

library(CLEAN)

reac <- getFunctionalCategories("reactome", "Hs")

go.bp <- getFunctionalCategories("GO", "Hs", "BP");names(go.bp) <- "GO.BP"

go.cc <- getFunctionalCategories("GO", "Hs", "CC");names(go.cc) <- "GO.CC"

go.mf <- getFunctionalCategories("GO", "Hs", "MF");names(go.mf) <- "GO.MF"

#arm 1 vs. arm 2 (all)

proteins <- c("IL2", "IL1B", "TNFAIP8", "RABGAP1L", "MYO9B", "PRDX3", "ICA1", "IRAK1", "TRAF2", "NUB1", "PSPN", "SH2D1A", "FOXO1", "METAP1D", "TBC1D5", "DAPP1", "TRIM5", "TRIM21", "SAMD9L", "SCRN1", "IKBKG", "NFATC1", "PSMG3", "GOPC", "MAP2K6", "NT5C3A", "ARHGEF12", "CASP2", "BCR", "CLIP2", "NCF2", "IRAK4", "DECR1", "AXIN1", "MGMT", "HEXIM1", "BANK1", "FKBP1B", "DFFA", "EIF4G1", "DNAJA2", "IL16", "GMPR", "PPP1R9B", "PRDX5", "CXCL9", "DBNL", "PKLR", "NCK2", "MANF", "SHMT1", "PTPN6", "FIS1", "PDLIM7", "CRKL", "DNPH1", "LHPP", "ATP5IF1", "SKAP2", "ANKRD54", "STX16", "PDP1", "RNF41", "MZT1", "RASSF2", "CA11", "KLK12", "MAVS", "ZBTB16", "STX6", "DDX58", "SUGT1", "CHAC2", "STXBP3", "AKR1B1", "ATG4A", "CRACR2A", "MAP3K5", "ACAA1", "TPMT", "FOXO3", "STAT5B", "SF3B4", "ABL1", "TACC3", "TBC1D23", "AIFM1", "LPCAT2", "LAT2", "CDKN2D", "PSMD9", "GRPEL1", "AARSD1", "INPPL1", "CDKN1A", "KIFBP", "ERBIN", "SRP14", "STX4", "SCAMP3", "DCTN2", "ELOA", "PPME1", "YES1", "IQGAP2", "DNAJB1", "USO1", "S100A12", "SIRT2", "CALCOCO1", "HAO1", "DCTN1", "PRDX6", "CASP8", "HBQ1", "APEX1", "RILP", "METAP2", "FXN", "CD5", "LTA4H", "SORD", "HDGF", "HAGH", "PPP1R12A", "ATOX1", "TJAP1", "CIAPIN1", "SNAP29", "MSRA", "SRC", "HNRNPK", "CTF1", "IRAG2", "COMT", "MPHOSPH8", "GZMH", "GYS1", "PAG1", "USP8", "ITGB1BP2", "AK1", "CNST", "SNX9", "DOK2", "AGXT", "TGM2", "PLPBP", "PRKAR1A", "LACTB2", "FADD", "MNDA", "EIF4EBP1", "GPNMB", "CD2AP", "DIABLO", "LEP", "GRAP2", "CD69", "COL4A1", "SNAP23", "CA13", "HSPB1", "KYAT1", "CORO1A", "CASP3", "THBS4", "SOD1", "CA1", "SMARCA2", "SLC27A4", "FGR", "PADI4", "CETN2", "GGA1", "TBC1D17", "PTS", "PMVK", "CASP1", "ILKAP", "BAX", "CARHSP1", "PRTFDC1", "TARBP2", "FHIT", "TDRKH", "MAX", "WASF3", "FMNL1", "AKT1S1", "MAP4K5", "DARS1", "MAD1L1", "SKAP1", "PTPN1", "EBAG9", "ABHD14B", "CRADD", "APRT", "FKBP5", "TBCC", "RHOC", "TXLNA", "STAMBP", "DNMBP", "CC2D1A", "MITD1", "NPM1", "PRDX1", "IPCEF1", "RBKS", "TBCB", "EIF4B", "RWDD1", "VTA1", "PDCD5", "NSFL1C", "PPCDC", "SULT1A1", "LBR", "ANXA3", "ENO1", "FABP5", "CPPED1", "BIN2", "MIF", "STIP1", "AHSP", "PEBP1", "SERPINB1", "MESD", "NUDT5", "FYB1", "BLVRB", "CA2", "PARK7")

geneIDs <- unlist(mget(proteins, org.Hs.egSYMBOL2EG))

allGenes <- keys(org.Hs.egSYMBOL)

gle <- rbind(data.frame(type="GO.CC", geneListEnrichment(geneIDs,

allGenes, go.cc[[1]], sigFDR=0.1)),

data.frame(type="GO.BP", geneListEnrichment(geneIDs,

allGenes, go.bp[[1]], sigFDR=0.1)),

data.frame(type="GO.MF", geneListEnrichment(geneIDs,

allGenes, go.mf[[1]], sigFDR=0.1)),

data.frame(type="Reactome", geneListEnrichment(geneIDs,

allGenes, reac[[1]], sigFDR=0.1)))

gle <- gle[order(gle[, 5]), ]

### Fig. S1c, d

d2 <- as.data.frame(merged[merged$FASFL == "Y", c("USUBJID", "ACTARMCD",

"SVRResponse2", "Visit", "OlinkID", "NPX")])

d2 <- d2[d2$Visit %in% c("BSLN"), ]

d2$Arm <- gsub("B", "Arm 2", gsub("A", "Arm 1", d2$ACTARMCD))

d2$protein <- olink_assay_data$Assay[match(d2$OlinkID, olink_assay_data$OlinkID)]

d_subset <- d2[d2$protein %in% c("IRAK4" , "FGR" , "TRIM5"), ] ## immune

p<-ggplot(d_subset, aes(x=Arm, y=NPX)) +

xlab(" ") + ylab("protein expression (NPX)") + #theme(legend.position="none") +

facet_grid( ~ protein, scale="free_x") + #scale_colour_manual(values=cbbPalette) +

#theme(axis.text.x = element_text(angle = 90, vjust = 0.5, hjust=1)) +

#labs(title=d_subset$title[1]) + #theme(plot.title=element_text(hjust=0.5, size=8, family="Albany AMT")) +

#geom_errorbar(aes(ymin=m-se, ymax=m+se), width=.02, position=position_dodge(.05)) +

#geom_line(aes(group=USUBJID)) +

geom_boxplot(outlier.shape = NA) +

geom_jitter(width = 0.1) + theme_bw()

print(p)

d_subset <- d2[d2$protein %in% c("CASP3" , "GZMH" , "CASP8"), ] ## apoptosis

p<-ggplot(d_subset, aes(x=Arm, y=NPX)) +

xlab(" ") + ylab("protein expression (NPX)") + #theme(legend.position="none") +

facet_grid( ~ protein, scale="free_x") + #scale_colour_manual(values=cbbPalette) +

#theme(axis.text.x = element_text(angle = 90, vjust = 0.5, hjust=1)) +

#labs(title=d_subset$title[1]) + #theme(plot.title=element_text(hjust=0.5, size=8, family="Albany AMT")) +

#geom_errorbar(aes(ymin=m-se, ymax=m+se), width=.02, position=position_dodge(.05)) +

#geom_line(aes(group=USUBJID)) +

geom_boxplot(outlier.shape = NA) +

geom_jitter(width = 0.1) + theme_bw()

print(p)

### Fig. 2c–e

## 4) plot by SVRResponse

proteinsToPlot <- c("TNF", "IL10", "IL12A_IL12B")

for(assay in proteinsToPlot) {

if(assay == "TNF") protein <- "OID21237" ## choose one of the four Olink IDs

else protein <- olink_assay_data$OlinkID[match(assay, olink_assay_data$Assay)]

subset <- merged[merged$FASFL == "Y", c("ACTARMCD", "SVREOS1", "Visit", "OlinkID",
 "Assay", "NPX")]

subset$NPX <- subset$NPX - merged$NPX.BSLN[merged$FASFL == "Y"]

d3 <- as.data.frame(subset %>% group_by(ACTARMCD, SVREOS1, Visit, OlinkID) %>%

summarise(m = mean(NPX, na.rm=TRUE),

sd = sd(NPX, na.rm=TRUE),

N = length(na.omit(NPX))))

d3$Visit <- factor(d3$Visit, levels=c("BSLN", "BEPI_WK03", "BEPI_WK05", "BEPI_WK08",

"BEPI_WK12", "BEPI_WK24","PEGIFN_WK01", "PEGIFN_WK02",

"PEGIFN_WK04", "PEGIFN_WK06", "PEGIFN_WK13", "PEGIFN_WK20",

"OT_WK01", "OT_WK04", "OT_WK12", "OT_WK24"))

d3$title <- paste0(d3$OlinkID, ": ", olink_assay_data$Assay[match(d3$OlinkID,

olink_assay_data$OlinkID)], " (", olink_assay_data$UniProt[match(

d3$OlinkID, olink_assay_data$OlinkID)], ")")

d3$se <- 1.98 * d3$sd/sqrt(d3$N)

d_subset <- d3[d3$OlinkID == protein, ]

d_subset$SVREOS1 <- gsub("Sustained Virologic Response", "SVR", d_subset$SVREOS1)

p<-ggplot(d_subset, aes(x=Visit, y=m, color=SVREOS1, group=SVREOS1)) +

xlab("time") + ylab("mean protein expression (cfb)") +

facet_grid( ~ ACTARMCD, scale="free_x") +

theme(axis.text.x = element_text(angle = 90, vjust = 0.5, hjust=1)) +

labs(title=d_subset$title[1]) +

geom_errorbar(aes(ymin=m-se, ymax=m+se), width=.02,

position=position_dodge(.05)) +

geom_line(aes(group=SVREOS1)) + geom_point()

print(p)

}

### Fig. 4b

### ALT

alt <- lb[lb$LBTESTCD == "ALT", c("USUBJID", "AVISIT", "AVISITN", "AVAL")]

alt <- alt[alt$AVISIT %in% visits$AVISIT, ]

alt <- alt[alt$AVISIT %in% names(table(alt$AVISIT))[table(alt$AVISIT) > 10], ]

sag <- lb[lb$LBTESTCD == "HBSAG" & lb$PARAM ==

"SERUM OR PLASMA Hepatitis B Virus Surface Antigen (log10 IU/mL)",

c("USUBJID", "AVISIT", "AVISITN", "AVAL")]

sag <- sag[sag$AVISIT %in% visits$AVISIT, ]

sag <- sag[sag$AVISIT %in% names(table(sag$AVISIT))[table(sag$AVISIT) > 10], ]

### correlation between ALT and proteins

d <- alt

d$OlinkVisit <- visits$OlinkVisit[match(d$AVISIT, visits$AVISIT)]

d$OlinkVisit[d$AVISIT == "BASELINE"] <- "BSLN"

d <- d[!is.na(d$OlinkVisit), c("USUBJID", "AVISIT", "AVAL", "OlinkVisit")]

colnames(d) <- c("USUBJID", "AVISIT", "ALT", "Visit")

d <- unique(merge(d, merged[, c("USUBJID", "OlinkID", "Assay", "Visit", "NPX")]))

alt_cor <- sapply(unique(d$Assay), function(assay)

cor(log2(d$ALT[d$Assay == assay]), d$NPX[d$Assay == assay]))

alt_cor_wk08 <- sapply(unique(d$Assay), function(assay)

cor(log2(d$ALT[d$Assay == assay & d$Visit == 'BEPI_WK08']),

d$NPX[d$Assay == assay & d$Visit == 'BEPI_WK08']))

### correlation between sag and proteins

d <- sag

d$OlinkVisit <- visits$OlinkVisit[match(d$AVISIT, visits$AVISIT)]

d$OlinkVisit[d$AVISIT == "BASELINE"] <- "BSLN"

d <- d[!is.na(d$OlinkVisit), c("USUBJID", "AVISIT", "AVAL", "OlinkVisit")]

colnames(d) <- c("USUBJID", "AVISIT", "SAG", "Visit")

d <- unique(merge(d, merged[, c("USUBJID", "OlinkID", "Assay", "Visit", "NPX",

"ACTARMCD")]))

sag_cor <- sapply(unique(d$Assay), function(assay)

cor(d$SAG[d$Assay == assay], d$NPX[d$Assay == assay]))

d <- alt

colnames(d)[colnames(d) == "AVAL"] <- "ALT"

d <- merge(d, alt[alt$AVISIT == "BASELINE", c("USUBJID", "AVAL")], all = TRUE) # add baseline values

colnames(d)[colnames(d) == "AVAL"] <- "ALT.BSLN"

d <- merge(d, sag, all = TRUE)

colnames(d)[colnames(d) == "AVAL"] <- "SAG"

d <- merge(d, sag[sag$AVISIT == "BASELINE", c("USUBJID", "AVAL")], all = TRUE) # add baseline values

colnames(d)[colnames(d) == "AVAL"] <- "SAG.BSLN"

d$OlinkVisit <- visits$OlinkVisit[match(d$AVISIT, visits$AVISIT)]

d$OlinkVisit[d$AVISIT == "BASELINE"] <- "BSLN"

d <- d[!is.na(d$OlinkVisit), c("USUBJID", "AVISIT", "ALT", "ALT.BSLN", "SAG",

"SAG.BSLN", "OlinkVisit")]

colnames(d)[colnames(d) == "OlinkVisit"] <- "Visit"

d <- unique(merge(d, merged[, c("USUBJID", "OlinkID", "Assay", "Visit", "NPX",

"NPX.BSLN")]))

sag_cor_wk08 <- sapply(unique(d$Assay), function(assay)

cor((d$SAG-d$SAG.BSLN)[d$Visit == "BEPI_WK08" & d$Assay == assay],

(d$NPX)[d$Visit == "BEPI_WK08" & d$Assay == assay], use="p"))

### put together in data.frame and make heatmap

sag_alt_assoc <- data.frame(assay=names(sag_cor),

sag_overall=sag_cor,

stringsAsFactors = FALSE)

sag_alt_assoc$sag_week8 <- sag_cor_wk08[match(sag_alt_assoc$assay,

names(sag_cor_wk08))]

sag_alt_assoc$alt_overall <- alt_cor[match(sag_alt_assoc$assay,

names(alt_cor))]

sag_alt_assoc$alt_week8 <- alt_cor_wk08[match(sag_alt_assoc$assay,

names(alt_cor_wk08))]

assays <- c("DCXR", "SCLY", "CA5A", "GSTA3", "C19orf12", "KRT18", "RBP5", "SORD",

"ACY1", "PBLD", "HNMT", "PTS", "GFER", "HTRA2", "KYNU", "SHMT1", "ADH4",

"AIFM1", "GSTA1", "HAO1", "SULT2A1", "MVK", "DDAH1", "ACAA1", "AGXT")

### make heatmap of correlation coefficients

draw(ComplexHeatmap::Heatmap(as.matrix(sag_alt_assoc[assays, -1]),

name = "Pearson Correlation",

cluster_columns = FALSE,

cluster_rows = TRUE,

show_row_names = TRUE,

show_column_names = TRUE,

column_names_gp = gpar(fontsize = 10),

row_names_gp = gpar(fontsize = 5),

column_title_gp = gpar(fontsize = 7),

heatmap_legend_param = list(

labels_gp = gpar(fontsize = 7)),

border = TRUE,

row_title_rot = 0))

### Fig. 4c

d <- alt

d$OlinkVisit <- visits$OlinkVisit[match(d$AVISIT, visits$AVISIT)]

d$OlinkVisit[d$AVISIT == "BASELINE"] <- "BSLN"

d <- d[!is.na(d$OlinkVisit), c("USUBJID", "AVISIT", "AVAL", "OlinkVisit")]

colnames(d) <- c("USUBJID", "AVISIT", "ALT", "Visit")

d <- unique(merge(d,merged[,c("USUBJID","ACTARMCD","OlinkID","Assay","Visit","NPX")]))

ggplot(d[d$Assay == "AIFM1" & d$Visit == "BEPI_WK08", ],

aes(log10(ALT), NPX, col=ACTARMCD)) + geom_point() +

geom_smooth(method = "lm") + ylab("AIFM1 expression") +

xlab("log10 ALT") + ggtitle("Bepi Week 8")

with(d[d$Assay == "AIFM1" & d$Visit == "BEPI_WK08", ], cor(log10(ALT), NPX))

### Fig. 4d, e

## 4) plot by SVRResponse

proteinsToPlot <- c("AIFM1", "KRT18")

for(assay in proteinsToPlot) {

protein <- olink_assay_data$OlinkID[match(assay, olink_assay_data$Assay)]

subset <- merged[merged$FASFL == "Y", c("ACTARMCD", "SVREOS1", "Visit", "OlinkID",

"Assay", "NPX")]

subset$NPX <- subset$NPX - merged$NPX.BSLN[merged$FASFL == "Y"]

d3 <- as.data.frame(subset %>% group_by(ACTARMCD, SVREOS1, Visit, OlinkID) %>%

summarise(m = mean(NPX, na.rm=TRUE),

sd = sd(NPX, na.rm=TRUE),

N = length(na.omit(NPX))))

d3$Visit <- factor(d3$Visit, levels=c("BSLN", "BEPI_WK03", "BEPI_WK05", "BEPI_WK08",

"BEPI_WK12", "BEPI_WK24","PEGIFN_WK01",

"PEGIFN_WK02", "PEGIFN_WK04", "PEGIFN_WK06",

"PEGIFN_WK13", "PEGIFN_WK20",

"OT_WK01", "OT_WK04", "OT_WK12", "OT_WK24"))

d3$title <- paste0(d3$OlinkID, ": ", olink_assay_data$Assay[match(d3$OlinkID,

olink_assay_data$OlinkID)], " (", olink_assay_data$UniProt[match(

d3$OlinkID, olink_assay_data$OlinkID)], ")")

d3$se <- 1.98 * d3$sd/sqrt(d3$N)

p<-ggplot(d_subset,

aes(x=Visit, y=m, color=SVREOS1, group=SVREOS1)) + xlab("time") +

ylab("mean protein expression (cfb)") +

facet_grid( ~ ACTARMCD, scale="free_x") +

theme(axis.text.x = element_text(angle = 90, vjust = 0.5, hjust=1)) +

labs(title=d_subset$title[1]) +

geom_errorbar(aes(ymin=m-se, ymax=m+se), width=.02,

position=position_dodge(.05)) +

geom_line(aes(group=SVREOS1)) + geom_point()

print(p)

}

### Fig. 5

dna <- lb[lb$LBTESTCD == "HBVDNA", c("USUBJID", "AVISIT", "AVISITN", "AVAL")]

dna <- dna[dna$AVISIT %in% visits$AVISIT, ]

dna <- dna[dna$AVISIT %in% names(table(dna$AVISIT))[table(dna$AVISIT) > 10], ]

extractedData <- NULL

for(usubjid in c(sl$USUBJID[sl$SVREOS2 == "Complete Response" & sl$ACTARMCD == "A"],

sl$USUBJID[sl$SVREOS2 == "Null Response" & sl$ACTARMCD == "A"])) {

arm <- unique(sl$ACTARMCD[sl$USUBJID == usubjid])

## ALT

d_subset <- alt[alt$USUBJID == usubjid, ]

if(arm == "A") {

d_subset$AVISIT_numeric <- visits$week_armA[match(d_subset$AVISIT, visits$AVISIT)]

vline1 <- 24.5

vline2 <- 48.5

} else {

d_subset$AVISIT_numeric <- visits$week_armB[match(d_subset$AVISIT, visits$AVISIT)]

vline1 <- 12.5

vline2 <- 36.5

}

palt <-ggplot(d_subset,

aes(x=AVISIT_numeric, y=(AVAL), group=USUBJID)) +

xlab("") + ylab("") +

geom_line(aes(group=USUBJID)) + geom_point()+

geom_line(aes(group=USUBJID)) + geom_point() +

scale_y_continuous(limits=c(0, max(c(d_subset$AVAL, 600)))) +

scale_x_continuous(limits = c(-1, 72))+

geom_vline(xintercept = vline1, col="orange", lty=2, lwd=1.3) +

geom_vline(xintercept = vline2, col="orange", lty=2, lwd=1.3)

## HBsAg

d_subset <- sag[sag$USUBJID == usubjid, ]

if(arm == "A") {

d_subset$AVISIT_numeric <- visits$week_armA[match(d_subset$AVISIT, visits$AVISIT)]

vline1 <- 24.5

vline2 <- 48.5

} else {

d_subset$AVISIT_numeric <- visits$week_armB[match(d_subset$AVISIT, visits$AVISIT)]

vline1 <- 12.5

vline2 <- 36.5

}

psag<-ggplot(d_subset,

aes(x=AVISIT_numeric, y=AVAL, group=USUBJID)) +

xlab("") + ylab("") +

geom_line(aes(group=USUBJID)) + geom_point() +

scale_y_continuous(limits = c(-1.4, 4.5))+

scale_x_continuous(limits = c(-1, 72))+

geom_vline(xintercept = vline1, col="orange", lty=2, lwd=1.3) +

geom_vline(xintercept = vline2, col="orange", lty=2, lwd=1.3)

## DNA

d_subset <- dna[dna$USUBJID == usubjid, ]

if(arm == "A") {

d_subset$AVISIT_numeric <- visits$week_armA[match(d_subset$AVISIT, visits$AVISIT)]

vline1 <- 24.5

vline2 <- 48.5

} else {

d_subset$AVISIT_numeric <- visits$week_armB[match(d_subset$AVISIT, visits$AVISIT)]

vline1 <- 12.5

vline2 <- 36.5

}

pdna<-ggplot(d_subset,

aes(x=AVISIT_numeric, y=AVAL, group=USUBJID)) + xlab("") + ylab("") +

geom_line(aes(group=USUBJID)) + geom_point() +

scale_y_continuous(limits=c(0, max(c(d_subset$AVAL, 180)))) +

scale_x_continuous(limits = c(-1, 72))+

geom_hline(yintercept = 20, lty=2, col="blue") +

geom_vline(xintercept = vline1, col="orange", lty=2, lwd=1.3) +

geom_vline(xintercept = vline2, col="orange", lty=2, lwd=1.3)

figure <- ggarrange(pdna + rremove("x.text"), psag + rremove("x.text"), palt,

ncol = 1, nrow = 3, align = "v", labels=NULL,

heights = c(1, 1, 1), common.legend = TRUE)

print(figure)

}

### Fig. S4

d_subset <- alt

d_subset$AVISIT <- factor(d_subset$AVISIT, levels=visits$AVISIT)

d_subset$ACTARMCD <- sl$ACTARMCD[match(d_subset$USUBJID, sl$USUBJID)]

d_subset$SVREOS2 <- sl$SVREOS2[match(d_subset$USUBJID, sl$USUBJID)]

d_subset <- d_subset[d_subset$USUBJID %in% intersect(olink_data$USUBJID,

sl$USUBJID[sl$FASFL == "Y"]), ]

p<-ggplot(d_subset[d_subset$SVREOS2 %in% c("Complete Response", "Null Response"), ],

aes(x=AVISIT, y=log(AVAL, 10), color=SVREOS2, group=USUBJID)) +

xlab("time") + ylab("log10 ALT") +

facet_grid(SVREOS2 ~ ACTARMCD, scale="free_x") +

theme(axis.text.x = element_text(angle = 90, vjust = 0.5, hjust=1)) +

labs(title='ALT') +

geom_line(aes(group=USUBJID), alpha=0.25) + #+ geom_point()

stat_summary(fun=mean, geom="line", lwd= 1.25, aes(group=1))

print(p)

### Fig. S5

flow_sas <- as.data.frame(read_sas("f_bio_flow_eot_2.sas7bdat"))

flow_sas$SVREOS1 <- sl$SVREOS1[match(flow_sas$USUBJID, sl$USUBJID)]

d_subset <- flow_sas[flow_sas$paramlbl == "Act T CD8+ CD38+ HLA-DR+ %" &

flow_sas$trtseqat == "Treatment: Overall (n*=26)" &

!is.na(flow_sas$AVAL), ]

d <- as.data.frame(d_subset %>% group_by(AVISIT) %>%

summarise(m = mean(AVAL, na.rm=TRUE),

sd = sd(AVAL, na.rm=TRUE),

N = length(na.omit(AVAL))))

d$AVAL <- d$m

d$AVISIT <- factor(d$AVISIT, levels=levels(flow_sas$AVISIT))

d$se <- 1.98 * d$sd/sqrt(d$N)

d$ymi <- d$AVAL - d$se

d$yma <- d$AVAL + d$se

d_subset <- merge(d_subset, d[, c("AVISIT", "ymi", "yma")])

p<-ggplot(d_subset, aes(x=AVISIT, y=AVAL, ymin=ymi, ymax=yma)) +

xlab("Visit") + ylab(d_subset$paramlbl[1]) + #ylab("Result") +

facet_grid( ~ trtseqat, scale="free_x") +

labs(title=paste("Marker:", d_subset$paramlbl[1])) +

geom_line(aes(group=USUBJID), alpha=0.4, col="blue", lty=3, linewidth=1) +

stat_summary(aes(group=trtseqat), fun=mean, geom="line", colour="blue",

linewidth=1.2) +

geom_ribbon(aes(x = as.numeric(AVISIT)), alpha=0.25, fill = "blue") +

theme(text = element_text(size = 20), axis.text.x =

element_text(angle = 90, hjust = 1, vjust=0.5)) + stat_n_text()

print(p+theme(panel.background = element_rect(fill = "white", color="black", size=1)))

d_subset <- flow_sas[flow_sas$paramlbl == "Prolif B %" &

flow_sas$trtseqat == "Treatment: Overall (n*=22)" &

!is.na(flow_sas$AVAL), ]

d <- as.data.frame(d_subset %>% group_by(AVISIT) %>%

summarise(m = mean(AVAL, na.rm=TRUE),

sd = sd(AVAL, na.rm=TRUE),

N = length(na.omit(AVAL))))

d$AVAL <- d$m

d$AVISIT <- factor(d$AVISIT, levels=levels(flow_sas$AVISIT))

d$se <- 1.98 * d$sd/sqrt(d$N)

d$ymi <- d$AVAL - d$se

d$yma <- d$AVAL + d$se

d_subset <- merge(d_subset, d[, c("AVISIT", "ymi", "yma")])

p<-ggplot(d_subset, aes(x=AVISIT, y=AVAL, ymin=ymi, ymax=yma)) +

xlab("Visit") + ylab("Result") +

facet_grid( ~ trtseqat, scale="free_x") +

labs(title=paste("Marker:", d_subset$paramlbl[1])) +

geom_line(aes(group=USUBJID), alpha=0.4, col="blue", lty=3) +

stat_summary(aes(group=trtseqat), fun=mean, geom="line",

colour="blue", linewidth=1.2) +

geom_ribbon(aes(x = as.numeric(AVISIT)), alpha=0.25, fill = "blue") + stat_n_text()

print(p+theme(panel.background = element_rect(fill = "white", color="black", size=1)))

### Fig. S8

proteinsToPlot <- c("ADH4", "CA5A")

for(assay in proteinsToPlot) {

protein <- olink_assay_data$OlinkID[match(assay, olink_assay_data$Assay)]

subset <- merged[merged$FASFL == "Y", c("ACTARMCD", "SVREOS1", "Visit", "OlinkID",

"Assay", "NPX")]

subset$NPX <- subset$NPX - merged$NPX.BSLN[merged$FASFL == "Y"]

d3 <- as.data.frame(subset %>% group_by(ACTARMCD, SVREOS1, Visit, OlinkID) %>%

summarise(m = mean(NPX, na.rm=TRUE),

sd = sd(NPX, na.rm=TRUE),

N = length(na.omit(NPX))))

d3$Visit <- factor(d3$Visit, levels=c("BSLN", "BEPI_WK03", "BEPI_WK05", "BEPI_WK08",

"BEPI_WK12", "BEPI_WK24","PEGIFN_WK01",

"PEGIFN_WK02", "PEGIFN_WK04", "PEGIFN_WK06",

"PEGIFN_WK13", "PEGIFN_WK20",

"OT_WK01", "OT_WK04", "OT_WK12", "OT_WK24"))

d3$title <- paste0(d3$OlinkID, ": ",

olink_assay_data$Assay[match(d3$OlinkID, olink_assay_data$OlinkID)],

" (", olink_assay_data$UniProt[match(d3$OlinkID,

olink_assay_data$OlinkID)], ")")

d3$se <- 1.98 * d3$sd/sqrt(d3$N)

d_subset <- d3[d3$OlinkID == protein, ]

p<-ggplot(d_subset,

aes(x=Visit, y=m, color=SVREOS1, group=SVREOS1)) +

xlab("time") + ylab("mean protein expression (cfb)") +

facet_grid( ~ ACTARMCD, scale="free_x") +

theme(axis.text.x = element_text(angle = 90, vjust = 0.5, hjust=1)) +

labs(title=d_subset$title[1]) +

geom_errorbar(aes(ymin=m-se, ymax=m+se),width=.02, position=position_dodge(.05)) +

geom_line(aes(group=SVREOS1)) + geom_point()

print(p)}

### Fig. S10

proteinsToPlot <- c("AIFM1", "KRT18", "ADH4", "CA5A")

for(assay in proteinsToPlot) {

protein <- olink_assay_data$OlinkID[match(assay, olink_assay_data$Assay)]

subset <- merged[merged$FASFL == "Y", c("ACTARMCD", "SVREOS2", "Visit", "OlinkID",

"Assay", "NPX")]

d <- as.data.frame(subset %>% group_by(ACTARMCD, SVREOS2, Visit, OlinkID) %>%

summarise(m = mean(NPX, na.rm=TRUE),

sd = sd(NPX, na.rm=TRUE),

N = length(na.omit(NPX))))

d$Visit <- factor(d$Visit, levels=c("BSLN", "BEPI_WK03", "BEPI_WK05", "BEPI_WK08",

"BEPI_WK12", "BEPI_WK24","PEGIFN_WK04",

"PEGIFN_WK06", "PEGIFN_WK13", "PEGIFN_WK20",

"OT_WK01", "OT_WK04", "OT_WK12", "OT_WK24"))

d$title <- paste0(d$OlinkID, ": ", olink_assay_data$Assay[match(d$OlinkID,

olink_assay_data$OlinkID)], " (", olink_assay_data$UniProt[

match(d$OlinkID, olink_assay_data$OlinkID)], ")")

d$se <- 1.98 * d$sd/sqrt(d$N)

d_subset <- d[d$OlinkID == protein & d$SVREOS2 %in%

c("Sustained Virologic Response", "Null Response"), ]

p<-ggplot(d_subset,

aes(x=Visit, y=m, color=SVREOS2, group=SVREOS2)) +

xlab("time") + ylab("mean protein expression (cfb)") +

facet_grid( ~ ACTARMCD, scale="free_x") +

theme(axis.text.x = element_text(angle = 90, vjust = 0.5, hjust=1)) +

labs(title=d_subset$title[1]) +

geom_errorbar(aes(ymin=m-se,ymax=m+se), width=.02, position=position_dodge(.05)) +

geom_line(aes(group=SVREOS2)) + geom_point()

print(p)}

### Load microarray data

load("data4BiomarkerPaper_microarray.RData")

bsln_data <- data[, pdata$Visit == "BSLN"]

tmp <- pdata$USUBJID[pdata$Visit == "BSLN"]

if(length(tmp) < length(unique(pdata$USUBJID))) {

tmp <- c(tmp, setdiff(pdata$USUBJID, tmp))

bsln_data <- cbind(bsln_data, NA)

}

bsln_data <- bsln_data[, match(pdata$USUBJID, tmp)]

### Figure 3A

d <- sapply(lvls, function(x) rowMeans((data-bsln_data)[,

paste(pdata$arm, pdata$SVREOS, pdata$Visit, sep=".") == x], na.rm=TRUE))

rownames(d) <- fdata$geneID

index <- fdata$geneID %in% genesToPlot

col_annot1 <- ComplexHeatmap::columnAnnotation(

ACTARM=sapply(lvls, function(str) strsplit(str, split="\\.")[[1]][1]),

SVRResponse = sapply(lvls, function(str) strsplit(str, split="\\.")[[1]][2]),

AVISIT=sapply(lvls, function(str) strsplit(str, split="\\.")[[1]][3]),

col = list( SVRResponse = c("Sustained Virologic Response" = "red",

"No Sustained Virologic Response" = "blue",

"Partial Response" = "white",

"Relapse" = "grey"),

AVISIT = c("BSLN" = "lightblue",

"BEPI_WK05"= "darkolivegreen3",

"BEPI_WK12" = "forestgreen",

"PEGIFN_WK01" = "orange",

"PEGIFN_WK02" = "darkorange",

"PEGIFN_WK04" = "darkorange1",

"PEGIFN_WK13" = "darkorange3",

"OT_WK01" = "darkslategray1",

"OT_WK12" = "darkslategrey",

"OT_WK24" = "black"),

ACTARM = c("A" = "purple","B" = "yellow")),

annotation_legend_param = list(labels_gp = gpar(fontsize = 4)),

gp = gpar(col = "black")

)

draw(ComplexHeatmap::Heatmap(d[index, ],

name = "mean expression (log2)",

col = circlize::colorRamp2(c(-1., 0, 1.),

c("blue", "white", "red")),

top_annotation = col_annot1,

column_split = gsub(" Response.*", "Response", lvls),

cluster_columns = FALSE,

cluster_rows = TRUE,

show_row_names = TRUE,

show_column_names = FALSE,

column_names_gp = gpar(fontsize = 4),

row_names_gp = gpar(fontsize = 10),

column_title = "SVR at EoS", #paste(arm, "-", msvr),

column_title_gp = gpar(fontsize = 7),

heatmap_legend_param = list(

labels_gp = gpar(fontsize = 4)),

border = TRUE,

row_title_rot = 0 ))

### Fig. 3b, c

genes <- c("TYMS", "MS4A1")

for(gene in genes) {

usp18 <- unlist((data-bsln_data)[which(fdata$geneID == gene), ])

d <- data.frame(USP18=usp18,

USUBJID=pdata$USUBJID,

AVISIT=pdata$AVISIT,

ACTARM=pdata$ACTARM,

SVREOS2=pdata$SVREOS2,

stringsAsFactors = FALSE)

d <- d[-grep("ELIGIBILITY", d$AVISIT), ]

d$SVREOS1 <- sl$SVREOS1[match(d$USUBJID, sl$USUBJID)]

subset <- d[, c("ACTARM", "SVREOS1", "AVISIT", "USP18")]

d2 <- as.data.frame(subset %>% group_by(ACTARM, SVREOS1, AVISIT) %>%

summarise(m = mean(USP18, na.rm=TRUE),

sd = sd(USP18, na.rm=TRUE),

N = length(na.omit(USP18))))

d2$AVISIT <- case_match(d2$AVISIT,

"BASELINE" ~ "BSLN",

"WK 5 DAY 29" ~ "BEPI_WK05",

"WK 12 DAY 78" ~ "BEPI_WK12",

"PEGIFN DOSING WK 1 DAY 1" ~ "PEGIFN_WK01",

"PEGIFN DOSING WK 4 DAY 1" ~ "PEGIFN_WK04",

"PEGIFN DOSING WK 13 DAY 1"~ "PEGIFN_WK13",

"OT-W1 OT-DAY 1" ~ "OT_WK01",

"OT-W12 OT-DAY 78" ~ "OT_WK12",

"OT-W24 OT-DAY 162" ~ "OT_WK24",

.default = d2$AVISIT)

d2$AVISIT <- factor(d2$AVISIT, levels=c("BSLN", "BEPI_WK05", "BEPI_WK12",

"PEGIFN_WK01", "PEGIFN_WK04", "PEGIFN_WK13",

"OT_WK01", "OT_WK12", "OT_WK24"))

d2$se <- 1.98 * d2$sd/sqrt(d2$N)

p<-ggplot(d2, aes(x=AVISIT, y=m, color=SVREOS1, group=SVREOS1)) +

xlab("time") + ylab("mean mRNA expression") +

facet_grid( ~ ACTARM, scales = "free_x") +

theme(axis.text.x = element_text(angle = 90, vjust = 0.5, hjust=1)) +

labs(title=gene) +

geom_errorbar(aes(ymin=m-se, ymax=m+se), width=.02,position=position_dodge(.05)) +

geom_line(aes(group=SVREOS1)) + geom_point()

print(p)

}

### Load data

load("data4BiomarkerPaper_Heatmaps.RData")

### Fig. 2a

proteinsToPlot <- unique(c(f5(comparison = "cfb_pooled", timepoint = "BEPI_WK03"),

f5(comparison="cfb_bySVRResponse",timepoint="BEPI_WK03"),

f5(comparison = "cfb_byArm", timepoint = "BEPI_WK03")))

subset <- merged[merged$FASFL == "Y", c("ACTARMCD", "SVREOS1", "Visit",

"OlinkID", "Assay", "NPX")]

subset$NPX <- subset$NPX - merged$NPX.BSLN[merged$FASFL == "Y"]

d <- as.data.frame(subset %>% group_by(ACTARMCD, SVREOS1, Visit, OlinkID) %>%

summarise(m = mean(NPX, na.rm=TRUE)))

d <- pivot_wider(d, names_from = "OlinkID", values_from = "m")

d <- d[order(as.numeric(d$Visit)), ]

d_mean_npx <- t(as.matrix(d[, -(1:3)]))

index <- TRUE

col_annot1 <- ComplexHeatmap::columnAnnotation(

SVRResponse = d$SVREOS1[index],

ACTARM=d$ACTARMCD[index],

AVISIT=d$Visit[index],

col = list( SVRResponse = c("Sustained Virologic Response" = "red",

"No Sustained Virologic Response" = "blue",

"Partial Response" = "white",

"Relapse" = "grey"),

AVISIT = c("BSLN" = "lightblue",

"BEPI_WK03" ="darkolivegreen1",

"BEPI_WK05"= "darkolivegreen3",

"BEPI_WK08" = "darkolivegreen",

"BEPI_WK12" = "forestgreen",

"BEPI_WK24"="darkgreen",

"PEGIFN_WK01" = "orange",

"PEGIFN_WK02" = "darkorange",

"PEGIFN_WK04" = "darkorange1",

"PEGIFN_WK06" = "darkorange2",

"PEGIFN_WK13" = "darkorange3",

"PEGIFN_WK20"= "darkorange4",

"OT_WK01" = "darkslategray1",

"OT_WK04" = "darkslategray3",

"OT_WK12" = "darkslategrey",

"OT_WK24" = "black"),

ACTARM = c("A" = "purple","B" = "yellow")),

annotation_legend_param = list(labels_gp = gpar(fontsize = 4)),

gp = gpar(col = "black")

)

draw(ComplexHeatmap::Heatmap(as.matrix(d_mean_npx[rownames(d_mean_npx) %in%

proteinsToPlot, index]),

name = "Change from \nBsln (log2)",

col = circlize::colorRamp2(c(-1.5, 0, 1.5),

c("blue", "white", "red")),

top_annotation = col_annot1,

column_split = paste0(d$ACTARMCD[index],

d$SVREOS1[index]),

cluster_columns = FALSE,

cluster_rows = TRUE,

show_row_names = TRUE,

show_column_names = FALSE,

column_names_gp = gpar(fontsize = fsize),

row_names_gp = gpar(fontsize = fsize),

column_title = "DEP at week 3", #paste(arm, "-", msvr),

column_title_gp = gpar(fontsize = 7),

heatmap_legend_param = list(

labels_gp = gpar(fontsize = 4)),

border = TRUE,

row_title_rot = 0))

### Fig. 4a

subset <- merged[merged$FASFL == "Y", c("ACTARMCD", "SVREOS1", "Visit",

"OlinkID", "Assay", "NPX")]

subset$NPX <- subset$NPX - merged$NPX.BSLN[merged$FASFL == "Y"]

d <- as.data.frame(subset %>% group_by(ACTARMCD, SVREOS1, Visit, OlinkID) %>%

summarise(m = mean(NPX, na.rm=TRUE)))

d <- pivot_wider(d, names_from = "OlinkID", values_from = "m")

d <- d[order(as.numeric(d$Visit)), ]

d_mean_npx <- t(as.matrix(d[, -(1:3)]))

rownames(d_mean_npx) <- olink_assay_data$Assay[match(rownames(d_mean_npx),

olink_assay_data$OlinkID)]

d_annot <- d[, 1:3]

index <- TRUE

col_annot1 <- ComplexHeatmap::columnAnnotation(

SVRResponse = d$SVREOS1[index],

ACTARM=d$ACTARMCD[index],

AVISIT=d$Visit[index],

col = list( SVRResponse = c("Sustained Virologic Response" = "red",

"No Sustained Virologic Response" = "blue",

"Partial Response" = "white",

"Relapse" = "grey"),

AVISIT = c("BSLN" = "lightblue",

"BEPI_WK03" ="darkolivegreen1",

"BEPI_WK05"= "darkolivegreen3",

"BEPI_WK08" = "darkolivegreen",

"BEPI_WK12" = "forestgreen",

"BEPI_WK24"="darkgreen",

"PEGIFN_WK01" = "orange",

"PEGIFN_WK02" = "darkorange",

"PEGIFN_WK04" = "darkorange1",

"PEGIFN_WK06" = "darkorange2",

"PEGIFN_WK13" = "darkorange3",

"PEGIFN_WK20"= "darkorange4",

"OT_WK01" = "darkslategray1",

"OT_WK04" = "darkslategray3",

"OT_WK12" = "darkslategrey",

"OT_WK24" = "black"),

ACTARM = c("A" = "purple","B" = "yellow")),

annotation_legend_param = list(labels_gp = gpar(fontsize = 4)),

gp = gpar(col = "black") )

draw(ComplexHeatmap::Heatmap(as.matrix(d_mean_npx[rownames(d_mean_npx) %in%

proteinsToPlot, index]),

name = "Change from \nBsln (log2)",

col = circlize::colorRamp2(c(-1.5, 0, 1.5),

c("blue", "white", "red")),

top_annotation = col_annot1,

column_split = paste0(d$ACTARMCD[index],

d$SVREOS1[index]),

cluster_columns = FALSE,

cluster_rows = TRUE,

show_row_names = TRUE,

show_column_names = FALSE,

column_names_gp = gpar(fontsize = fsize),

row_names_gp = gpar(fontsize = 10),

column_title = "SVR at EoS (cfb)",

column_title_gp = gpar(fontsize = 7),

heatmap_legend_param = list(

labels_gp = gpar(fontsize = 4)),

border = TRUE,

row_title_rot = 0) )

## References

1. Buti M, Heo J, Tanaka Y, Andreone P, Atsukawa M, Cabezas J et al. Sequential Peg-IFN after bepirovirsen may reduce post-treatment relapse in chronic hepatitis B. J Hepatol. 2025;82(2):222-34.

2. Yuen MF, Lim SG, Plesniak R, Tsuji K, Janssen HLA, Pojoga C et al. Efficacy and Safety of Bepirovirsen in Chronic Hepatitis B Infection. N Engl J Med. 2022;387(21):1957-68.

3. Cremer J, Elston R, Campbell FM, Kendrick S, Paff M, Quinn G et al. B-Clear Phase 2b Study Design: Establishing the Efficacy and Safety of Bepirovirsen in Patients with Chronic Hepatitis B Virus Infection. Adv Ther. 2023;40(9):4101-10.

4. CLSI. Validation of Assays Performed by Flow Cytometry. 1st Edition. CLSI guideline H62. Clinical and Laboratory Standards Institute; 2021. 2021. <https://clsi.org/standards/products/hematology/documents/h62/>.

5. Gautier L, Cope L, Bolstad BM, Irizarry RA. affy--analysis of Affymetrix GeneChip data at the probe level. Bioinformatics. 2004;20(3):307-15.

6. Dai M, Wang P, Boyd AD, Kostov G, Athey B, Jones EG et al. Evolving gene/transcript definitions significantly alter the interpretation of GeneChip data. Nucleic Acids Res. 2005;33(20):e175.

7. Johnson WE, Li C, Rabinovic A. Adjusting batch effects in microarray expression data using empirical Bayes methods. Biostatistics. 2007;8(1):118-27.

8. Filbin MR, Mehta A, Schneider AM, Kays KR, Guess JR, Gentili M et al. Longitudinal proteomic analysis of severe COVID-19 reveals survival-associated signatures, tissue-specific cell death, and cell-cell interactions. Cell Rep Med. 2021;2(5):100287.

9. Freudenberg JM, Joshi VK, Hu Z, Medvedovic M. CLEAN: CLustering Enrichment ANalysis. BMC Bioinformatics. 2009;10:234.

10. Carlson M. GO.db: A set of annotation maps describing the entire Gene Ontology. R package version 3.8.2. 2019. <https://bioconductor.org/packages/release/data/annotation/html/GO.db.html>.

11. Ligtenberg W. reactome.db: A set of annotation maps for reactome. R package version 1.68.0. 2019. <https://bioconductor.org/packages/devel/data/annotation/html/reactome.db.html>.

12. Szklarczyk D, Kirsch R, Koutrouli M, Nastou K, Mehryary F, Hachilif R et al. The STRING database in 2023: protein-protein association networks and functional enrichment analyses for any sequenced genome of interest. Nucleic Acids Res. 2023;51(D1):D638-D46.

13. Benjamini Y, Hochberg Y. Controlling the False Discovery Rate: A Practical and Powerful Approach to Multiple Testing. J R Stat Soc Series B Stat Methodol. 1995;57(1):289–300.
